# Expanding rural access to chronic pain care through nurse care management: A hybrid type I effectiveness-implementation trial protocol

**DOI:** 10.1371/journal.pone.0349526

**Published:** 2026-05-27

**Authors:** Brennan Keiser, Karina Cortez, Elise Hoffman, Jin Wang, Kelly Reeves, Andrew Humbert, Robert W. Bailey, Stacy Shaw Welch, Sophia Jawort, Laura-Mae Baldwin, Caleb Holtzer, Hazel Tapp, Thomas Ludden, Basia Belza, Kushang V. Patel, Sebastian T. Tong

**Affiliations:** 1 Department of Family Medicine, University of Washington, Seattle, Washington, United States of America; 2 Department of Anesthesiology & Pain Medicine, University of Washington, Seattle, Washington, United States of America; 3 Department of Family Medicine, Atrium Health Wake Forest Baptist, Charlotte, North Carolina, United States of America; 4 Department of Rehabilitation Medicine, University of Washington, Seattle, Washington, United States of America; 5 Department of Psychology, University of Washington, Seattle, Washington, United States of America; 6 Providence Northeast Washington Medical Group, Colville, Washington, United States of America; 7 Department of Biobehavioral Nursing and Health Informatics, University of Washington, Seattle, Washington, United States of America; PLOS: Public Library of Science, UNITED KINGDOM OF GREAT BRITAIN AND NORTHERN IRELAND

## Abstract

**Background:**

Chronic pain affects more than 20% of U.S. adults, with prevalence approaching 30% in rural communities. It can substantially impair physical functioning, mental health, and quality of life, and rural residents experience poorer pain-related outcomes. Effective care requires a comprehensive, multidisciplinary approach; however, rural primary care clinicians often rely on medications—particularly prescription opioids—as the predominant treatment despite limited evidence and substantial risks. Innovative strategies are needed to expand access to evidence-based, non-pharmacologic pain management in rural settings.

**Objective:**

To determine the effectiveness of a nurse care management (NCM) model for chronic pain compared with usual care and to evaluate its implementation.

**Methods:**

A pragmatic, hybrid type I effectiveness–implementation randomized controlled trial will be conducted from April 2026 to July 2028. A total of 450 adults with chronic pain and a Pain, Enjoyment of Life, and General Activity (PEG) scale total score ≥12 will be recruited from rural health systems. Rural eligibility will be verified using the Health Resources and Services Administration (HRSA) Rural Health Grants Eligibility Analyzer. Participants will be randomly assigned to the NCM intervention or to usual care using permuted block randomization with variable block sizes (4 or 6). The 6-month virtually delivered NCM intervention includes (1) cognitive behavioral therapy, (2) care coordination, and (3) referral to *Tele-Enhance®Fitness*, an instructor-led virtual group exercise program. The primary outcome is pain interference and intensity measured by the PEG scale. Secondary outcomes include pain catastrophizing, depression, anxiety, physical functioning, sleep disturbance, satisfaction with health, and opioid use. Implementation outcomes—reach, fidelity, feasibility, and sustainment—will be evaluated with the Practical, Robust Implementation and Sustainability Model (PRISM) framework.

**Discussion:**

This pragmatic trial will assess the effectiveness of the NCM model for chronic pain care and collect implementation data to guide potential scale-up in rural primary care.

**Trial registration:**

ClinicalTrials.gov identifier: NCT06407115.

## Introduction

Chronic pain affects more than 20% of U.S. adults, with prevalence approaching 30% in rural regions [1,2]. The annual economic burden of chronic pain in the United States is estimated at $722.8 billion [3], encompassing both medical care costs and lost work productivity [2]. Chronic pain exerts profound effects on physical functioning, mental health, and overall quality of life [4]—outcomes that are consistently poorer among rural residents [2,5]. This disparity is partly driven by the higher concentration of older adults and manual laborers in rural communities [6], but it also reflects systemic barriers like healthcare workforce shortages and limited availability of multidisciplinary services [2]. Patients in rural primary care settings are less likely to receive referrals to pain specialists and more likely to rely on medications—particularly prescription opioids—as their primary treatment [7]. Yet evidence shows that opioids have limited long-term efficacy for chronic pain and carry substantial risks [8]. Interventions that expand access to evidence-based, non-pharmacologic pain management in rural settings are needed to improve equity and health outcomes.

Drawing from effective models for other chronic conditions such as substance use [9,10], diabetes [11] and depression [12], our team adapted a virtual nurse care management (NCM) model for chronic pain (S1 File) that demonstrated feasibility and acceptability during the pilot [13]. Delivered over six months, the intervention integrates three evidence-based components: (1) cognitive-behavioral therapy (CBT) [14], (2) care planning and coordination [15], and (3) referral to *Tele-Enhance®Fitness* (Tele-EF), an instructor-led virtual group exercise program [16]. Rooted in the biopsychosocial framework [17], this bundled approach aims to strengthen self-management skills, expand access to multimodal, non-pharmacologic therapies, and promote tailored exercise participation.

The primary aim of this pragmatic, hybrid type I effectiveness–implementation trial is to evaluate the effectiveness of the adapted NCM model in reducing pain interference and intensity compared to usual care. The secondary aim is to assess the feasibility and potential for sustainment by exploring how contextual determinants influence implementation outcomes and documenting implementation strategies across sites. We hypothesize that this approach will demonstrate that the NCM model is effective in addressing chronic pain among rural residents and will inform scalable implementation models, if successful.

## Methods

### Ethical approval and data monitoring

This trial was approved by the University of Washington Institutional Review Board (IRB) (STUDY00019595) and registered on ClinicalTrials.gov (NCT06407115) on May 9^th^, 2024. The study is considered minimal risk, and this protocol was prepared in accordance with the Standard Protocol Items: Recommendations for Intervention Trials (SPIRIT) reporting guideline (S3 File) [18]. Participant safety, adverse events, and study conduct will be monitored by an independent Data Safety Monitoring Board (DSMB), which is composed of experts in clinical care, ethics, biostatistics, exercise and psychology. The DSMB reviewed and approved the protocol and analysis plan (S2 File) on August 25, 2025. It will convene again after the first 30 participants complete the study and every 6 months thereafter. The DSMB may recommend continuation, modification, or termination of the study based on safety or operational concerns. Additional details are provided in the DSMB Charter (S4 File).

### Trial status

The trial opened recruitment on April 1^st^, 2026 and currently follows protocol version 1.4, April 1st, 2026. Further amendments to the protocol will be approved by the IRB of record and then communicated to sites through the reliance infrastructure and routine study meetings. Trial recruitment is expected to finish within 16 months and within the original timeline (by July 31^st^, 2027). Data collection will be complete by August 2028 with results published shortly thereafter.

### Study design and frameworks

**Fig 1:**
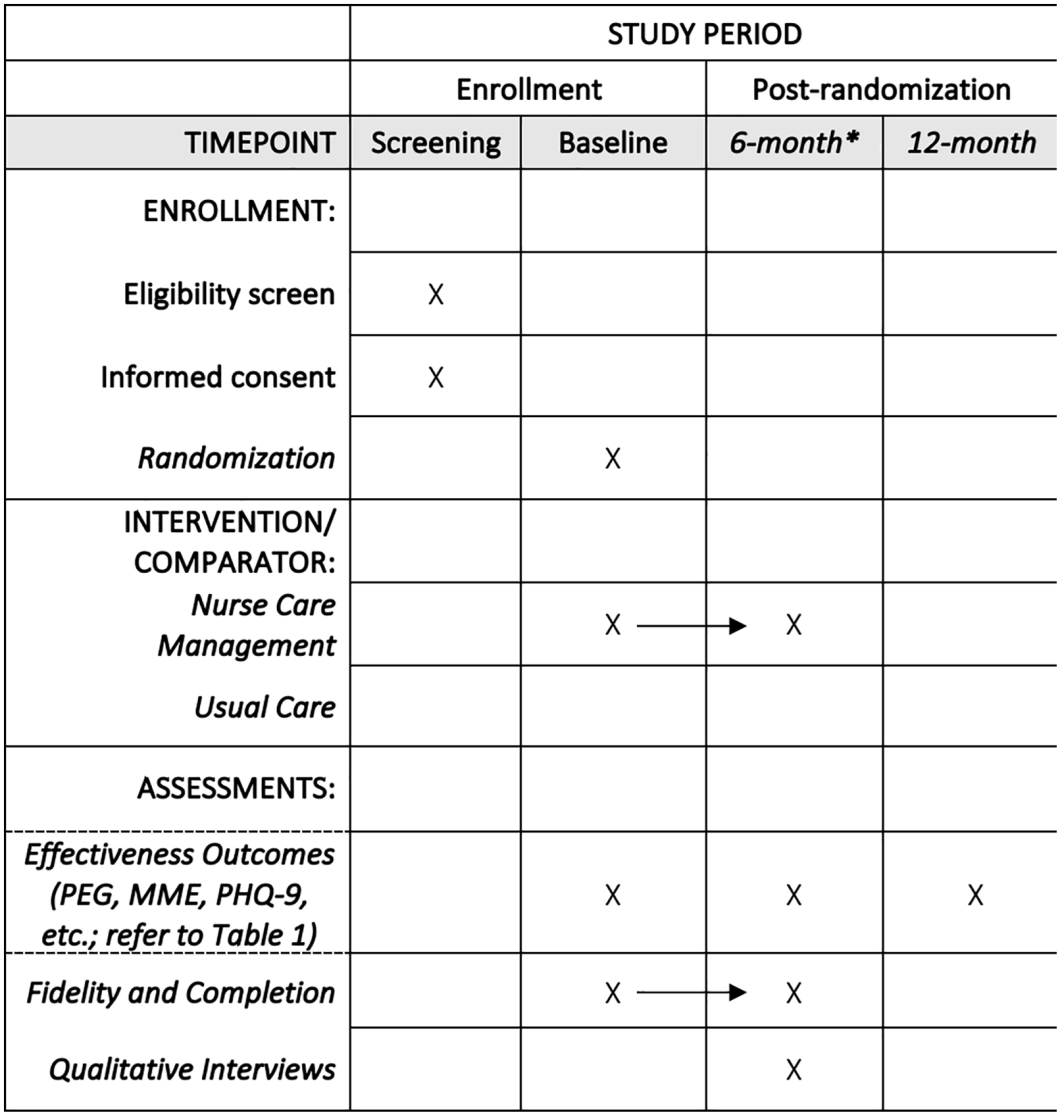
Participant timeline: Schedule of enrollment, interventions, and assessments. *Represents the immediate post-intervention assessment. NCM intervention participants’ first visit will be scheduled within 2 weeks of randomization; the intervention is delivered continuously for 6 months.

This pragmatic, hybrid type I effectiveness–implementation partially nested, individually randomized group-treatment trial will be conducted from April 2026 to July 2028. Participants will be recruited over a 16-month period across rural-serving health systems and randomly assigned to either the NCM intervention or usual care to evaluate effectiveness. Analysis of the implementation data will be guided by the Practical, Robust Implementation and Sustainability Model (PRISM) framework [19]. Fig 1 provides a detailed schedule.

### Participants

Adults with chronic pain will be recruited through two practice-based research networks: the WWAMI region Practice and Research Network (Washington, Wyoming, Alaska, Montana, and Idaho) and the Mecklenburg Area Partnership for Primary Care Research in North Carolina. Sites will recruit participants through methods aligned with standard practice, including clinician referral, electronic health record (EHR)-based outreach, and distributing flyers for patient self-referral. Clinicians may opt out individual patients if participation would pose a safety concern or conflict to their current treatment plan. Eligible participants are adults aged ≥18 years who (1) live in a rural area, (2) are a current primary care patient in a participating health system with at least one visit in the last year, (3) speak English or Spanish proficiently, (4) have a documented chronic pain diagnosis [20,21] in the EHR, and (5) score ≥12 in total on the Pain, Enjoyment of Life, and General Activity (PEG) scale [22]. Exclusion criteria include moderate to severe cognitive impairment; residence in a nursing home or inpatient facility; receipt of palliative care; ongoing radiation treatment or chemotherapy; planned surgery requiring hospitalization within the intervention period; and participation in pain-related psychoeducation or skills training within the past six months.

### Screening, consent, and randomization

Prospective participants may complete the screening survey online or over the phone to determine eligibility. Prior to enrollment, a research coordinator (RC) will verify rural eligibility using the Health Resources and Services Administration (HRSA) Rural Health Grants Eligibility Analyzer [23]. If a respondent self-reports cognitive impairment, the RC will also assess moderate to severe impairment using the telephone Montreal Cognitive Assessment [24]. For participants who are eligible and interested, the RC will review the study details and document informed consent using a REDCap [25] e-consent module or paper forms sent by mail. Consented participants will also provide authorization under the Health Insurance Portability and Accountability Act (HIPAA) for access to and use of the minimum necessary personally identifiable health information to achieve the aims of the study. Randomization assignments will be generated by the UW Data Coordinating Center using permuted block randomization with random block sizes of 4 and 6. Enrolled participants will be assigned in a 1:1 ratio to either usual care or the NCM intervention. Given the nature of the study arms, it is not feasible to blind participants or site personnel; however, RCs that enroll participants will not have access to the randomization sequence.

### Usual care

Participants assigned to the usual care arm will be instructed to continue seeking care from their existing care team. Usual care will not be standardized and thus may vary across sites. It may include pharmacologic management, referrals to specialty services, or other pain-related treatments at the discretion of the treating clinician.

### Nurse care management (NCM) intervention

While not a requirement to join the study, clinical sites will be encouraged to identify a minimum of two care managers. This role redundancy is intended to provide coverage during periods of leave and mitigate any attrition. Aligned with real-world practice, each clinical site will decide how to distribute patients among the care managers. Sites will receive funding to support care manager time, including training and intervention delivery, as well as RC effort, EHR data extraction, and study administrative costs. The three intervention components have been manualized (Supplement 1) and are described briefly below.

#### Care planning and coordination.

Participants assigned to the NCM intervention will complete an initial 1-hour intake appointment with a care manager to develop a personalized chronic pain care plan. Before this session, participants will complete a social needs survey and pain and mood assessments using PainTracker [26], a digital self-report tool that supports measurement-based care. During the intake appointment, care managers will elicit the participant’s pain history, review PainTracker assessments, and support the participant in setting realistic pain management goals. In addition to clinical needs, care managers will address social determinants of health, including housing, nutrition, mental health, financial needs, employment, safety, and transportation. Based on identified priorities, care managers will connect participants with relevant resources, which may include referrals to a social worker, physical therapist, integrative health provider**,** or community organizations such as volunteer programs, community centers, libraries, churches, health departments, or educational institutions.

After intake, participants will meet with care managers for up to two sessions per month over six months for continued care coordination and goal support. Every 6–8 weeks, patients will complete follow-up assessments in PainTracker so care managers can monitor progress and adjust the care plan as needed. All sessions will be delivered via a HIPAA-compliant videoconferencing platform already in use at each health system.

#### CBT.

During follow-up sessions, care managers will deliver modular, CBT-informed pain self-management coaching to address behavioral and emotional barriers to physical function recovery, including depression, anxiety, sleep disturbance, trauma, and unhealthy substance use. Care managers will engage in shared decision-making with patients and leverage PainTracker assessments to select CBT skills most relevant to individual needs.

#### *Tele-EF*.

*Enhance®Fitness* is an evidence-based, community-based exercise program endorsed by the Centers for Disease Control and Prevention (CDC) for arthritis management [27]. This instructor-led program involves aerobic, balance, and strength training. Care managers will refer participants to Tele–EF and help them enroll in the remotely delivered version of the program [28,29]. Upon registration, patients will receive cuffed weights for exercise and, if needed, a cellular-enabled tablet to access classes. Before the first class, instructors will conduct a “zero session” to assess and support technology readiness. Participants may attend one-hour virtual exercise classes up to three times per week.

### Implementation strategies

#### Training and ongoing consultation.

Care managers will receive an intervention manual and video training modules covering all intervention procedures and CBT skills. Two clinical psychologists will conduct additional synchronous training before the trial. Once recruitment begins, these psychologists will provide biweekly group consultations. A primary care physician and physical activity expert will join monthly to discuss issues related to medical care coordination and Tele–EF.

#### Fidelity and quality monitoring.

Intervention fidelity will be monitored using a pragmatic fidelity checklist completed by care managers after each session. An implementation scientist will review these data and flag sessions scoring below the prespecified benchmark for review with the clinical psychologists. A fidelity score of 80% or greater, a commonly used threshold, will be considered acceptable [30–32]. Corrective action will focus on modifiable patterns addressed through targeted performance coaching or retraining as needed.

#### Learning collaborative and site tailoring.

Based on lessons from the pilot [13], a learning collaborative will be established to convene each participating health system’s implementation team, which will consist of a practice champion (e.g., a primary care physician or nurse leader), care manager(s), and RC at minimum. The collaborative will meet virtually for one hour up to six times annually. A primary care physician and implementation scientist will co-facilitate these meetings with three purposes: (1) to specify and document protocol-defined standard operating procedures; (2) to pragmatically tailor context-dependent implementation activities (e.g., community-based referrals, safety planning, and communication with primary care teams); and (3) to identify and address emerging barriers through collaborative problem-solving and continuous quality monitoring and improvement.

### Effectiveness outcome measures

All effectiveness outcomes are presented in Table 1. The primary outcome is pain interference as measured by the PEG scale. Secondary outcomes will include the National Institutes of Health (NIH) HEAL Initiative Common Data Elements for chronic pain including select measures from the Patient Reported Outcomes Measurement Information System (PROMIS) battery [33]. Patients will report most of these clinical outcomes in REDCap surveys online or through the mail at baseline, six months (immediately post-intervention), and 12 months. Patients will receive up to five reminders and be compensated $25 for baseline, $50 for post-intervention, and $75 for the 12-month follow-up. Opioid use data will be extracted from each participant’s EHR to calculate a morphine milligram equivalent (MME) dose at each time point.

**Table 1:** Effectiveness and implementation outcomes.

Outcome	Measure	Data Source
*Effectiveness*		
Pain Interference	PEG Scale	Patient Report
Physical Function	PROMIS Physical Function, short form 6b	Patient Report
Sleep	PROMIS Sleep Disturbance, short form 6a	Patient Report
Pain Catastrophizing	Pain Catastrophizing Scale (6-item)	Patient Report
Depression	Patient Health Questionnaire (9-item)	Patient Report
Anxiety	Generalized Anxiety Disorder (7-item)	Patient Report
Loneliness	UCLA Loneliness Scale (3-item)	Patient Report
Quality of Life	WHO Quality of Life (2-item)	Patient Report
Substance Use	Tobacco, Alcohol, Prescription medication and other Substance use tool (TAPS-1)	Patient Report
Healthcare Utilization	3-month history of primary care, emergency, integrative medicine, and pain-related procedures	Patient Report
Opioid Use	Morphine milligram equivalent	EHR Extraction
*Implementation*		
Reach	Rate of eligible participants who enroll	Screening data
Fidelity	Care manager checklist of core and modular intervention components	Self-report
Engagement (patient)	Care manager rating	Self-report
Completion	Care manager checklist	Self-report
Feasibility	Workflow indicators: time lag, referral rates, etc.; qualitative interviews	Mixed methods
Maintenance	12-month patient outcomes; qualitative interviews	Mixed methods

### Implementation outcome measures

Implementation outcomes will be collected using mixed methods across multiple data sources and are summarized in Table 1. Outcomes selection was guided by the iPRISM webtool [34] to characterize real-world feasibility, fidelity, and sustainability across rural primary care settings.

#### Reach.

We will operationalize reach as the proportion of eligible patients who enroll in the study at each site. All recruitment strategies will rely on the same proxy index event for expressing interest: responding to a brief pre-screening interest survey. Reasons for excluding participants during screening will also be reported. Given the heterogeneity typical in pragmatic primary care settings, we will assess whether demographic subgroups defined by age, sex assigned at birth, race/ethnicity, health insurance status, and employment status differ in their likelihood of enrolling.

#### Fidelity.

We will measure fidelity using a pragmatic checklist that care managers will fill out after each patient meeting. The checklist enumerates the intervention’s core components (i.e., active ingredients) and assesses coverage, frequency, and duration [35]. In addition, the checklist is designed to document co-occurring conditions (e.g., depression, anxiety, etc.) the patients are experiencing and assess, as indicated, the delivery of modular CBT content and skills.

#### Intervention completion and behavioral enactment.

Participant completion will be monitored through attendance logs and care managers will rate each participant’s engagement in appointments and care plan action items on a Likert scale. These data will be collected in REDCap using templates refined during the pilot. We will also survey participants at baseline, six months, and 12 months to capture the healthcare and integrated health services they utilized to manage their pain. This will help to illuminate how the NCM intervention may promote behavioral enactment outside of sessions and will also help to characterize usual care, highlighting differences across health systems.

#### Feasibility.

Feasibility will be assessed using quantitative indicators of intervention delivery and workflow efficiency: care manager training completion and case consultation attendance, median time lag from randomization to first visit, and rates of referral to Tele-EF that result in attending a first class. These data will be complemented by brief, qualitative interviews with patients and site personnel to characterize any intervention barriers or facilitators.

#### Maintenance.

For participants, maintenance will be measured as primary and secondary outcomes and healthcare utilization at the 12-month follow-up to explore whether changes in clinical outcomes and care-seeking behavior are sustained beyond the intervention period. At the provider level, qualitative interviews will explore the perceived potential to sustain implementation of the NCM intervention and future scale-up within primary care settings. This will include their views on clinic readiness, the feasibility of expanding the intervention across the health system, and the institutional infrastructure necessary for long-term integration.

### Qualitative interviews

In-depth interviews lasting approximately one hour will be conducted with participants assigned to the intervention arm and with health system representatives, purposefully sampling to stratify by site. Interview participants will be compensated $100 for their time. An interview guide will be adapted using templates from our pilot work and supplemented by the PRISM interview questions. Personnel across multiple roles and a diverse group of patients with differing levels of intervention completion will be invited to participate in interviews until saturation is achieved. The goals of the interviews are to explore implementation determinants that affect feasibility and examine the potential for sustainment. They also will help generate hypotheses about any differences in outcomes observed across the different sites or sub-populations.

### Implementation strategy tracking

It is well understood that implementation is a dynamic process that will evolve according to contextual changes, and natural variability across sites should be anticipated in pragmatic trials. For this reason, implementation strategies will be tracked using the Longitudinal Implementation Strategy Tracking System, which enables systematic capture of strategies at multiple points in the implementation process [36]. Research coordinators will meet with each site’s implementation team quarterly to fill out a REDCap tool designed to document these strategies and modifications over time.

### Sample size and power

A total of 450 adults with chronic pain will be recruited and randomized in a 1:1 ratio: 225 to each study arm. This sample size accounts for clustering within the intervention arm at the level of the care manager (intraclass correlation coefficient [ICC] = 0.05) and the Tele-EF instructor (ICC = 0.03). We assume 10 care managers with similar caseloads (approximately 22–23 participants each) and no more than five participants per Tele-EF instructor. Assuming a two-sided type I error rate of 0.05, a conservative 80% follow-up rate at 6 months, and a correlation of 0.30 between baseline and 6-month PEG scores, the study will have greater than 90% power to detect a moderate standardized effect size (Cohen’s d = 0.48) on the primary outcome. Under conservative assumptions, the study will also retain greater than 90% power to detect standardized effect sizes of d ≥ 0.40 for secondary outcomes.

### Data analysis

Baseline demographic characteristics will be summarized by treatment arm using descriptive statistics.

#### Effectiveness.

Primary effectiveness analyses will follow the intention-to-treat principle, regardless of intervention completion. Participants who disengage from the study activities will still be contacted via their preferred mode of communication to collect effectiveness outcomes. Treatment effects will be evaluated using outcome data collected at six months (post-intervention) and at 12-month follow-up. Linear mixed-effects models will include treatment assignment (NCM intervention vs. usual care), follow-up time, and their interaction as fixed effects. Participant and health system will be included as random effects to account for within-participant correlation over time and clustering within health systems. Care manager and Tele-EF instructor will be included as random effects in the NCM intervention arm only to account for provider-level correlation. In this parameterization, the coefficient corresponding to treatment assignment will provide an estimate of treatment effect at six months while the time-by-treatment interaction term will be used to estimate differential treatment effects between six and 12 months.

Maximum likelihood estimation with assumed unstructured covariance will be used to estimate parameters. If meaningful baseline imbalances are observed between treatment groups, corresponding covariates will be included in adjusted models. The primary outcome will be evaluated at a two-sided α of 0.05. Secondary outcomes will be adjusted for multiple comparisons using the Hochberg sequential procedure.

Missing data will be addressed using mixed-model estimation under a Missing at Random (MAR) assumption. Sensitivity analyses will include complete-case analyses and multiple imputation approaches if non-random missingness is observed. Exploratory subgroup analyses will examine whether treatment effects vary by age, sex, or delivery language (i.e., English or Spanish); however, the trial is not powered to detect subgroup effects, and such analyses will be considered hypothesis-generating.

#### Implementation.

Quantitative and qualitative findings will be integrated using a convergent mixed methods approach [37]. Implementation outcomes—fidelity, feasibility, completion, and reach—will be summarized using descriptive statistics overall and by site. Quantitative indicators will include measures of intervention delivery (e.g., training completion and core and modular component fidelity), participant engagement (e.g., attendance and participation in recommended activities), and intervention reach and retention. These data will be used to characterize implementation feasibility and inform interpretation of effectiveness findings.

Qualitative data from interviews with participants, care managers, consulting clinicians, and health system representatives will be analyzed using thematic analysis. Two members of the research team will independently code transcripts using an inductive coding framework based on the PRISM domains, with discrepancies resolved through discussion and consensus. Emerging themes will be used to identify barriers, facilitators, and adaptations relevant to real-world implementation and sustainment.

### Data management plan

Sensitive data extracted from the EHR will be stored securely on HIPAA-compliant servers at the coordinating site for subsequent analysis. At the time of consent, patients will sign a HIPAA authorization to release the minimum PHI necessary to achieve the aims of the study. In addition, all participating health systems will execute a Data Transfer and Use Agreement according to institutional guidelines.

### Safety considerations

Potential safety concerns include a breach of private and confidential data, psychological distress from the discussion of sensitive issues during the intervention, and muscle soreness and pain flares resulting from exercise. All adverse events will be documented and reported to the IRB in accordance with regulatory requirements. The risk for breach of privacy and confidentiality is minimized by our data management plan as described above. The care manager will attempt to address any psychological distress in the context of their sessions with participants and offer resources and referrals. Given the elevated risk of suicidal ideation among individuals with chronic pain [38], trained study personnel will monitor for suicidal thoughts at each survey time point, assess risk using the Columbia–Suicide Severity Rating Scale [39], and facilitate timely clinical evaluation or referral as appropriate. Muscle soreness and pain flares may be expected after exercise, especially for those who are sedentary. These are expected to be temporary and not require medical care.

### Dissemination of trial findings

Upon trial completion, the results will be reported via peer-reviewed publications and ClinicalTrials.gov. Authorship will follow the standards set by the International Committee of Medical Journal Editors. Statistical code and the full protocol for the study will be disseminated with a peer-reviewed publication, as appropriate. Plain language summaries will be created and shared with clinical partners through the practice-based research networks.

### Data sharing plan

To meet the requirements of the NIH HEAL Initiative Public Access and Data Sharing policy [40], primary data generated through this study will be shared in a publicly accessible data repository, the National Institute of Mental Health Data Archive. The data will be de-identified to the extent possible to maintain the privacy and confidentiality of participants.

## Discussion

Drawing on successful interventions for other chronic conditions, nurse care management for chronic pain has the potential to transform pain management in rural communities across the United States. This innovative model focuses on increasing access to and enhancing the delivery of evidence-based, non-pharmacologic treatments for chronic pain that frequently are unavailable for rural-dwelling individuals.

The primary limitation of our study is potential selection bias, because both participants and practices that agree to participate in the study may not represent the general rural population or the typical rural-serving primary care practice. We mitigated this by offering our study broadly to practices in two practice-based research networks and by embedding care managers within health systems to normalize this NCM model.

The intervention tested and implemented in this study uses the biopsychosocial model to manage chronic pain. If effective, our current trial will prepare rural-serving primary care practices to implement and sustain a nurse-delivered intervention that could substantially reduce pain intensity and interference while improving overall well-being and function in rural patients with chronic pain. Bolstered by current efforts to move payment away from the fee-for-service model and toward incentive structures that support team-based primary care, this NCM model has the potential to reshape the way care is delivered in rural primary care practices.

## Supporting information

S1 File Nurse care manager implementation manual. (DOCX)

S2 File Research protocol v. 1.4. (DOCX)

S3 File Spirit 2025 completed checklist. (DOCX)

S4 File Data safety and monitoring board charter. (DOCX)
