# Supplementary material for: Expanding rural access to chronic pain care through nurse care management: A hybrid type I effectiveness-implementation trial protocol
Source: PLoS One. 2026 May 27;21(5):e0349526. doi: 10.1371/journal.pone.0349526 (PMC13215515; doi:10.1371/journal.pone.0349526)
Supplement: S1 File — (DOCX) [file pone.0349526.s001.docx]

**AIM-CP Study Manual: Nurse Care Management for Chronic Pain**

**Expanding rural access to chronic pain care through nurse care management: a hybrid type I effectiveness-implementation trial protocol**

**Developed and led by investigators at the University of Washington**

**Funded by the National Institute on Nursing Research (UH3NR020930)**

Contents

[General Orientation 3](#_Toc226977098)

[Pre-meeting Activities 9](#_Toc226977099)

[Session 1 (Step 1: CARES) 12](#_Toc226977100)

[Session 2 (Step 1: CARES) 15](#_Toc226977101)

[Session 3 (Step 2: Grow) 19](#_Toc226977102)

[Session 4 (Step 3: Bend, Don’t Break OR Step 2: Grow) 22](#_Toc226977103)

[Session 5 and onward (Deliver CBT 1-2-3 and Care Management) 25](#_Toc226977104)

[Final Session (Relapse Prevention and Terminate Care Management and CBT 1-2-3 Care) 27](#_Toc226977105)

[Asynchronous Training Component: Description of Modules 28](#_Toc226977106)

[Appendix A: PainTracker Measures 31](#_Toc226977107)

[Appendix B: EHR Documentation Template 34](#_Toc226977108)

[Appendix C: Relapse Prevention Plan Worksheet 35](#_Toc226977109)

[Appendix D: Scale Worksheet 36](#_Toc226977110)

[Appendix E: 3B’s for Pain Worksheet 37](#_Toc226977111)

[Appendix F: Behavior Skills for Chronic Pain 38](#_Toc226977112)

[Appendix G: Activity Pacing 39](#_Toc226977113)

[Appendix H: Blank Pacing Worksheet 41](#_Toc226977114)

[Appendix I: Values 42](#_Toc226977115)

[Appendix J: Goal Setting and Creating Action Plans 43](#_Toc226977116)

[Appendix K: Body Skills 44](#_Toc226977117)

[Appendix L: Sleep Hygiene 47](#_Toc226977118)

[Appendix M: Cognitive Change 48](#_Toc226977119)

[Appendix N: Re-thinking the Pain Story 49](#_Toc226977120)

[Appendix O: Safety Protocol for Suicidality 51](#_Toc226977121)

[Appendix P: Breathing to Relax the Body 56](#_Toc226977122)

# General Orientation

What is Chronic Pain?

Chronic pain is common, costly, and often has a significant impact on quality of life, impacting work and social relationships. It is typically defined as pain that lasts three months or longer and may persist for years or even decades. Understandably, patients with chronic pain often spend a lot of time managing their pain in attempts to reduce their suffering, including pain reduction regimens, medical workups to find a cure, and surgical interventions. These efforts can be financially debilitating and are often ineffective. We often see patients caught in a vicious cycle where their lives are dominated by efforts to reduce pain that do not work and interfere with the parts of their lives that matter to them most. The more they focus exclusively on their pain, the more pain they find they are in from both a physical and emotional perspective. As the efforts to take the pain away fail and other parts of life that make it worth living recede, the pain becomes more dominant, and patients become more desperate to reduce it. This intervention is designed to help reduce this devastating vicious cycle. We introduce patients to strategies backed by research and focus on helping patients reclaim their lives even *with* pain. Our aim is to help them create new, healthier thoughts and habits to improving the quality of their lives while they manage their pain.

Research on how chronic pain works and what we can do to help typically emphasizes a biopsychosocial model, which means that a person’s biology/body, their psychology/mind, and their social world/environment are all important to consider. Because there are so many important factors that interact to make chronic pain worse, we have chosen an intervention based on Cognitive Behavioral Therapy (CBT), in the context of a collaborative care model. CBT is often only available in specialty mental health care settings, which is typically unavailable to many chronic pain patients, especially in rural areas. CBT is an evidence-based approach to help treat chronic pain which teaches patients how to cope with their pain by learning 1) new ways of calming and relaxing their *bodies* to manage pain, 2) new ways to *think* about pain and challenge thought patterns that are unhelpful, and 3) new *behavior patterns.* Our CBT-123 intervention, which we’ve developed to fit into a collaborative primary care delivery model, will help patients access these coping skills to set new goals and begin to reclaim their lives. A major emphasis in the CBT-123 treatment is to help patients reduce something called pain avoidance. Pain avoidance is a way many chronic pain patients have learned to cope, which research suggests makes things much worse. People sometimes reduce their activity levels to the point where they are less engaged with what gives their life meaning and vitality, and their freedom of movement is vastly reduced. The result might be reduced pain, but potentially at a terrible cost. For others, the reduction in activity may contribute to deconditioning, which can maintain pain. As such, one of the goals of CBT-123 is to help patients learn new coping skills to enable them to reduce their pain avoidance and increase activity in areas that matter to promote quality of life. In other words, the goal is to increase functioning, perhaps even with pain, with less emphasis on pain reduction strategies that have not contributed to long-term pain control. We hope that many patients who receive the CBT-123 intervention will be able to access the Tele- Enhance®Fitness protocols which are designed for people with chronic pain but can be intimidating and seem out of reach without new coping skills and education about chronic pain.

What follows in this manual is the CBT-123 cognitive-behavioral intervention for people living with chronic pain, which includes a variety of evidence-based tools and strategies and emphasizes flexibility such that the provider can individualize it for their patients. The research support for cognitive-behavioral interventions is strong and includes many different tools. Since each patient is different, we have designed the manual to be flexible, such that the tools can be emphasized or de-emphasized according to each patient’s needs and prior experiences. Patients may engage for different lengths of time according to their own pace and needs. We know that not all strategies will work for all people, and chronic pain patients are used to being given strategies that do not work. In CBT-123, we empower patients to be experts on their own experience, with the providers using the treatment to help guide options for change that might result in functional and meaningful improvement. While every strategy might not work for all people, a willingness to try out new practices and strategies, that is to “test it out yourself”, can be gently encouraged throughout. Over time, you and the patient will select the modules of treatment that are most important for them.

Another difficult experience most chronic pain patients have had is that of feeling invalidated by providers and the health care system in general. We have embedded time for the health care provider to listen and care for these patients in ways that are scientifically supported to enhance their willingness to try new tools for change, and the initial stages of this manual will guide providers in how to set this kind of supportive stage for change. The treatment outcomes targeted by this intervention focus on behavior change, anchored on what improvement would look like (i.e., changes that could be observed by you, or someone else and that are meaningful to the patient) if treatment were successful. This theme of observable change in the direction of values or what matters most will be reiterated throughout the treatment. With that treatment approach in mind, you’ll be using the cognitive-behavioral model to help people better understand their experience, work more effectively with barriers to change that are identified, and what particularly motivates patients themselves to make changes. You’ll be eliciting patients’ stories and helping them recognize ways to make changes in the areas of greatest importance that will improve quality of life, even with pain. Over time, you’ll work with them to set goals, track important behaviors, calm and relax their bodies, think in helpful ways, and re-engage in activities that make their lives worth living, pain or not.

*How* treatment is delivered is as important as *what* is delivered. The wider psychotherapy literature is clear that what accounts for significant change includes common factors like a sense of shared goals and a therapeutic alliance with an empathic therapist. The treatment provider is encouraged to take things slowly, as there is a lot of material to cover and if you rush, you risk losing people early on. Remember to encourage experimentation, especially outside of session. You are likely only seeing the patients biweekly; contact with the provider is limited, and learning will primarily happen between visits as patients try out new tools. On a final note, people with pain commonly seek out multiple workups in hopes of finding a diagnosis and ultimately a cure. As that comes up, you’ll want to validate that and continually reorient them to the potential benefits of making some changes right now, while they also seek diagnoses and a cure for chronic pain, that can improve quality of life, even when pain is present.

1. **Overview - Nurse Care Management in Primary Care for Chronic Pain**

Most rural-dwelling individuals with chronic pain receive treatment in primary care, given that demand has outstripped supply of specialty pain care services. Managing chronic pain in primary care is complex and is further challenged by a limited primary care workforce. Both patients and primary care clinicians would benefit from interventions and guidance to improve access to evidence-based, non-pharmacologic treatments.

Nurse care management (NCM) has been used effectively in other chronic conditions that require coordination between multiple specialties and services to prevent unnecessary hospital and emergency department use, to engage patients in shared decision-making and goal setting, and to alleviate burdens on primary care clinicians.

1. **Compile clinic resources for care coordination**

Review social needs survey and consider where you might refer patients who screen positive for any of the items on the survey. If you have a social worker or social services coordinator in your clinic, this may be a first step. While it is not within your scope to solve every social need that patients have, it will be important to have places or persons that patients can be referred to for more help. Resources that might be helpful to collate include the following

- Housing
- Food insecurity
- Transportation assistance
- Exposure to domestic or intimate partner violence
- Substance use treatment resources

Nurse care managers may need to look into local resources for stepping up mental health care, as well, since some patients will have needs outside the scope of this project (e.g., counseling and psychiatric care options).

1. **Overview of patient visits**

Patients will meet every two to four weeks with the nurse care manager for the duration of the six-month intervention. The CBT component of the intervention is focused on behavioral activation and addressing barriers to engaging in activities to help with pain (e.g., activity pacing). Care coordination includes referring patients for help with social needs, coordinating pain care between different providers, and ongoing monitoring of pain-related outcomes and functioning with PainTracker to help refine the care plan. Patients who are ready to engage in exercise can be referred to Tele-EF, a virtual, instructor-led group exercise class held three days a week for one hour.

When working with patients, make sure to leave time for doing notes; sessions can be 45 minutes. It can take multiple sessions to establish the therapeutic relationship. There is a six-month window for the NCM treatment; aim for one session per month unless the patient prefers more and time allows.

1. **Compile clinical resources for stepped mental health treatment options**

Clinic and specialty resources for mental health related to chronic pain will vary significantly based on the region, with rural locations typically having fewer resources. However, with a little creativity and an openness to trying different approaches, you can help patients connect to meaningful support. Here is a set of general recommendations for exploring options:

- Primary care providers with behavioral health or social work integration.
  - Encourage patients to ask their PCP about treatment options. Sometimes primary care clinics have embedded mental health resources. Social workers may provide mental health treatment and help patients connect with social services, like vocational rehab or financial assistance.
- Virtual mental health treatment.
  - Virtual care is especially valuable in rural areas. Mental health providers can see patients virtually from anywhere, as long as they are licensed in the state in which the patient is located. National platforms include Lyra, Talkspace, and BetterHelp.
- Federally Qualified Health Care (FQHC) centers
  - Community mental health centers are located in rural and urban areas. A quick web search can help find one that is nearby, offers telehealth, or both.
- Crisis and short-term support:
  - The national suicide helpline is 988 and supports calls or texts. Patients should be aware that the crisis line is a great 24/7 resource for support in any kind of crisis (not necessarily a suicide related crisis).
  - SAMHSA National Helpline’s phone number is 1-800-622-HELP and offers confidential, free support 24/7, with information for people experiencing mental health or substance abuse related disorders.
- Mobile apps and web support:
  - There are various apps that can support mental health and chronic pain needs, such as:
    - Curable – a chronic pain specific app
    - Mindfulness Coach – a general mindfulness app developed by the VA that is free and easy to navigate
    - Headspace, Calm, Insight Timer – additional apps for mindfulness and guided meditation
- General resources
  - Encourage patients to look for support groups and other online forums, such as Facebook groups
  - Many people who live with chronic pain report feeling better when they are connected to communities and stay active. The YMCA and senior centers, for example, are in many areas, are low priced, and offer a chance to stay active and meet new people.

1. **Measurement-based Care**

Patients will complete surveys on PainTracker, a measurement-based care tool. PainTracker will help track quantitative, patient-reported measures of pain, depression, anxiety and sleep disturbance. This can help you understand a patient’s current symptom and observe how they change over time through the course of treatment.

1. **How to introduce the Tele-Enhance®Fitness (Tele-EF) program**

In the first meeting, you will discuss Tele-EF during an overview of the intervention and review a readiness for change measure. When discussing the patient’s goals, if they identify exercise as a goal, consider discussing Tele-EF in more detail at that time. In following meetings, you’ll continue to check in on the patient’s readiness for physical movement as you work with them on the CBT-123 intervention and address barriers to pain management. You might consider exploring the patient’s preferred type of exercise, such as walking, swimming or yoga. When the patient feels comfortable that they can address barriers, and they are interested in Tele-EF, discuss and refer. The process is as follows: 1) they pick a class and send a referral to an instructor 2) the instructor contacts patients in one to three days via phone or email 3) the patient responds to the instructor to complete enrollment paperwork 4) the study team will send weights and tech, **after** the participant enrolls in the class.

1. **Asynchronous communications and lost to follow-up protocol**

Coordinate with the practice champion and study support staff in your clinic about reminder contact for appointments and what action to take when a patient does not show up for their scheduled NCM appointment.

For example, it may be helpful for the study support staff to text, email or call patients a few days before their appointment to remind them of their appointment with the care manager.

If patients do not show up to appointments, arrange for the study support staff to contact them to reschedule their appointment and troubleshoot ways to avoid future missed appointments.

1. **Communication with the Tele-EF team**

After submitting a referral, a Tele-EF instructor will contact the participant within three business days to complete the enrollment process. The instructor will continue to reach out until they have contacted the participant four times. At this point, they will send a message via the website to request that the NCM discuss the class with the patient again.

The NCM will then explore barriers to enrollment with the participant and address them. Some typical concerns and potential solutions include: 1) *Tech concerns* – remind the patient of available resources (study team provides internet-connected tablet, support to use tablet) 2) *Ability to participate in class* – Tele-EF is modifiable to all levels of functioning. It can be done seated and the instructor will work with the patient to adjust the class, so it works for them. If needed, the patient can be shown a video demonstration of class. 3) *Class scheduling* – review class offerings with patient and refer patient to different class as needed 4) *Challenges connecting with Tele-EF provider* – ensure patient’s preferred method of contact is correctly noted in the referral. Please leave a note to the Tele-EF provider with any additional information including best times of day to contact the patient and concerns to address when speaking with the patient.

After the participant completes the enrollment process, the UW study team will mail any technology that the patient needs along with the cuff weights to use in class. These materials will arrive within 10 days of the participants’ enrollment in Tele-EF.

1. **Communication with the primary care provider**

Communicating with the PCP can be an essential part of providing effective care. Before your first meeting with the patient, consider sending a message to the PCP informing them that the patient is participating in this study and will be getting six months of care management for their chronic pain. An example message template can be found in Appendix B. During the pain intervention, you may find yourself in a position where you are discussing a separate course of treatment, such as mental health care. Remember that the PCP may see the patient infrequently and your visits with the patient may be an important data point in the patient’s overall plan of care. It is generally recommended to keep the PCP informed of any changes in the treatment plan or outside treatments your patient is exploring. Situations that may come up that would be reasonable to share with the PCP include:

- The patient is interested in a specialty referral, such as physical therapy or a pain specialist.
- The patient would benefit from a medication discussion, e.g., to manage pain, mental health difficulties, or both.
- You are exploring an entirely new treatment direction, such as recommending patient focus on a course of care for a more pressing need, e.g., trauma-focused care for un- or under-treated PTSD.
- You’d like to share goals of care or a treatment success.
- You have noticed a setback and believe the PCP could benefit from the information.

Discuss with the practice champion and potentially other providers how these messages should be sent and the frequency with which messages should be sent to the PCP.

At the end of the intervention period, send a message to the PCP summarizing the intervention and key lessons/changes that have occurred in the intervention period highlighting any actions the PCP should take or items they might need to follow up on.

# Pre-meeting Activities

**1. Contact patient to schedule first meeting (in-person or virtual)**

Patients are identified for this study through either referrals from providers or electronic health record data pulls by research staff. The patients have consented to be part of the study and were told to expect contact from the care manager within two weeks after consenting to be in the study. Once a participant has consented, they will be invited to fill out the baseline survey. The baseline survey must be completed to finalize enrollment and meet with the care manager. Research staff will manage baseline surveys and add participants into Acuity Scheduling software. This resource has information about [using your client list](https://help.acuityscheduling.com/hc/en-us/articles/16676896712589-Managing-your-client-list-in-Acuity-Scheduling#gref).

As part of preparing for patients to be in the study, ensure that you have a call back number at work that patients can reach you at. If you are unable to reach the patient with the first call, leave a message to have them call you back and try again in a day (for up to three attempts). In addition, you’ll need to [set your availability](https://help.acuityscheduling.com/hc/en-us/articles/16676883635725-Managing-availability-and-calendars) in Acuity. If you choose, you may also use this resource to [send your Acuity appointments to your calendar](https://help.acuityscheduling.com/hc/en-us/articles/16676868807181-Syncing-appointments-with-third-party-calendars#gref). A one-way sync is recommended so that information is shared in one direction from Acuity to your calendar.

Using Acuity, schedule an hour to meet with the patient for the first meeting. This resource has information about [how to book, change or cancel appointments](https://help.acuityscheduling.com/hc/en-us/articles/16676870828685-Booking-appointments-for-clients-with-Acuity-Scheduling#gref) for patients. This initial booking may also be completed by research support staff on behalf of the care manager. The meeting should last about 45 minutes so that you have 15 minutes to document after meeting with the patient.

When scheduling the first meeting with the patient, you should inform them that they will be getting links to register and fill out the first PainTracker survey. You may also go over the social needs survey that will be used to inform their care (see items 2 and 3 below for details).

**2. Send PainTracker link to patients to complete**

PainTracker includes tools to evaluate patient’s pain level and how much pain interferes with their daily life, mental health measures (including depression, anxiety and PTSD), current physical activity level, sleep habits, substance use, and treatment goals. Patients will fill out the full PainTracker assessment before the first meeting so that it can be used to contextualize and understand the patient and their pain. Every month, patients will fill out an abbreviated version of PainTracker that includes pain level, pain interference, depression, anxiety and physical activity levels. This will be used to track patient progress over the course of the six months of care management. See Appendix A for more detailed description on how to use PainTracker.

*PainTracker Logistics:*

Research staff will help send the PainTracker login details to the patient after they complete the baseline survey. They will work with patients to make sure patients are registered in PainTracker and complete the first assessment before the initial appointment.

Patients must create an account/password to register. Research staff can add individual patients to PainTracker and then invite them to create an account. They will need to verify their name, email address, date of birth and set a password. After registration, links will connect patients directly to the assessment without logging in.

For subsequent appointments, the care manager will send invitations to patients to complete follow-up assessments once a month. Invite links can be sent from a patient’s summary page. Under “View Patient Records”, you will be able to see the last time a participant worked in PainTracker and if the status is “finished” or “in process.” If they haven’t completed the assessment, you can do it with them during a meeting.

**3. Complete social needs survey with participants**

The social needs survey includes questions about needs for housing, food, safety, dental, learning, transportation, social connections, work and finance. It also prompts patients to respond about whether they want help about each of these items. This will be completed once before the first visit with you with the primary purpose of determining how much each of these items is affecting the patient’s care and engagement in their pain treatment. Some items may be addressed in the CBT-123 as barriers.

*Social Needs Survey Logistics:*

Each patient has a social needs survey that is connected to the “Intake Appointment” in Acuity. When you (or a research team member) are booking the first appointment with the patient, you can complete this survey with patients. Another option is to complete the survey during the first appointment if it cannot be completed before.

You can access the Social Needs Survey while scheduling an appointment with a patient from the client list (see below) or by clicking on an appointment from the calendar and editing the appointment. The survey is nested in the “Forms and Notes” section.

**4. Review patient’s electronic health record (EHR) with a focus on their pain treatment history**

This activity will help you get a better sense of what treatments the patient has received already for their chronic pain. Spend no more than 15–20 minutes doing this for each patient and limit most review to things done in the past year.

Take a look at the following elements of the EHR:

- Patient’s last primary care visit and any discussion of pain
- Review patient’s medication list for any pain related medications
- Review any orthopedic, sports medicine, rheumatology, neurology, or pain medicine notes
- Review any physical therapy or other non-pharmacologic pain related visits
- Review recent emergency department or urgent care visits to see if they were pain related and, if yes, read in more detail

Make brief notes for yourself on this to remind yourself of the patient’s treatment history for the first meeting with the patient.

**5. Patient reminders three days before first meeting**

**6. Send message to patient’s primary care clinician to coordinate care**

If the patient was not referred directly from their primary care clinician, send a message to the primary care clinician to inform them that the patient is participating in nurse care management for chronic pain. An example message is provided in Appendix B.

# Session 1 (Step 1: CARES)

**(Introduction & CBT 1-2-3 Step 1: CARES – Collaborate, start Assessment, some Reflection)**

*There are a lot of potential activities for the first meeting. Do not rush the various elements. If you run out of time with the patient (the meeting should be no more than 45 minutes), move to scheduling a follow-up appointment with the patient in two weeks to continue with the first meeting activities.*

*Frequency of visits – aim for biweekly but can be weekly to monthly depending on patient needs and scheduling logistics across a six-month period.*

1. **Set agenda**
   - Overview of the program, care manager role, learn about pain story, review measures, start goal setting, set follow-up appointment
2. **Describe the program (5 min)**
   - Example script: “Our program involves me as a care manager, and I can meet with you regularly for up to six months. I’m here to help you coordinate your care related to your chronic pain and help you improve your life and reduce the way pain interferes in how you live. I can also help you engage a virtual exercise program as well.”
   - *Tele-EF:*
     - Example script: “The program also includes your ability to engage in Tele-EnhanceFitness, an online, instructor-led group exercise program that can accommodate patients with different levels of functioning. Group classes are held live, over Zoom, and involve aerobic, strength, and balance training. Classes are held three days a week for one hour for 16 weeks. The program is evidence-based and recommended by the CDC for arthritis and other painful conditions as well as falls prevention. And we can provide you with equipment at home to connect to Zoom if you need.”
3. **Collaborate/Assess – pain story (10 min)**
   - “Let’s take a few minutes to talk about what hurts, what you’d like to share with me about your pain?”
   - “First tell me briefly about your pain and thank you for filling out all our questionnaires. These would be good to review together as I get to know you better and work with you on goals for your treatment.”
   - “What’s worked and hasn’t worked to help you manage your pain?”
4. **Review measures (10–15 min)**
   - **PainTracker:** review results related to symptom levels (e.g., depression, anxiety), where and how severe is pain (e.g., pain intensity and pain interference levels), check if patient screens positive for PTSD on the PC-PTSD screener (positive = >3 for women and >4 for men), and any other relevant glaring issues (see Appendix A for detailed description of PainTracker measures)
   - **Review social needs:**
     - Look at the patient’s responses to see if there were any social needs identified. If the patient has not filled this out before the meeting, please go through the survey with them now. Discuss with the patient the social needs that were identified. If there are many that the patient screened positive for, focus on ones where the patient said they wanted help.
     - If it seems appropriate, connect patient with clinic resources or community-based organizations that can help patient with these social needs. Plan to revisit this at next visit to see what was done.
5. **Goal setting (10–15 min):**
   - Review PainTracker question about patient goals for participating in this study
   - Explain the goal of treatment for chronic pain is to improve quality of life and find ways to reduce the way pain interferes in our lives: “We always want and seek a cure for pain. And at the same time, we want to make sure we can have the best quality of life and reduce the way pain interferes in our lives. So, it’s not about doing one over the other but about doing both at the same time.”
   - Ask “If we succeeded together and this program helped you over the next few months, what would you be able to notice is better?”
     - If patient only talks about pain reduction, pivot to asking about what I or someone else would notice that’s different in your life – and validate that having the pain be less would be amazing.
   - “What kinds of activities in your life do you wish you could do again or do more of?”
     - Determine a list of patient-centered goals that relate to their function (i.e., what are meaningful activities they want to be able to do that align with their values and bring quality of life) and relate to the type of care they want to have happen (i.e., what type of medical care do they think they need)
6. **Set next appointment**
   - “I’d like to hear more at our next visit about what’s getting in the way of… (fill in with their words with the things they would like to be doing). Can we set up a time to meet again in the next week or two?”
   - Plan to meet in two weeks for one hour via audiovisual conferencing (i.e. Zoom) or phone. Between now and the next meeting, if needed encourage the patient to think critically about some long-term and short-term goals that they can discuss at the next meeting.
7. **Complete patient session/visit note in Acuity**
   - You can use the template in Appendix B to document in the EHR. The note in the EHR can be copy and pasted into the open answer field in Acuity. Please also check the areas that were addressed during this visit in Acuity.
   - Communicate with the primary care provider to let them know the patient is engaged in the program (e.g., copy note to the PCP in the EHR, e-message the PCP)

# Session 2 (Step 1: CARES)

**(Finish CBT 1-2-3 Step1: CARES – Collaborate, Assess, Reflect, Educate, Set goals)**

**The CARES step one of the CBT 1-2-3 intervention helps establish a collaborative, patient-centered relationship based on common goals and trust. Through the steps outlined below, you will complete the CARES step between sessions 1 and 2. We will talk with you in more detail during the synchronous training how each step of CARES is woven into the steps below.**

1. **Set agenda**

- Review pain story main points (“let’s reconnect”), deliver psychoeducation for chronic pain and do assessment for CBT 1-2-3 chronic pain goals, schedule follow-up appointment

1. **Reflect/Collaborate (2 min)**

- Give a brief summary of what you know are the primary concerns and what they’re needing (one to two sentences) (C, A, R)
- Thank the patient for telling the pain story last week and let them know that you are looking forward to learning more later in the session. Use patient-centered reflection to reflect what they told you.
- For example, “Thank you for telling me about how your back pain has affected your parenting. I really hear how much it means to you to take care of your daughter, and how much you’d like to get back to being able to take her to school every day again.”

1. **Educate (10 min)**

- Introduce the basic model of how pain cycles work. Explain that pain affects most people in this way and go through a very brief explanation with the patient. Make sure this makes sense to them and then move on to the next section.
- Show the patient this model and explain the gate theory of pain. “With chronic pain, some things can open the gate, and some can close it. We can also look at it like this, where some things will increase how much pain we feel, some things will decrease it. I’d like to ask you more about your pain and fill this out together so we can understand some of what you have learned already.” Ask about what has worked well to help with pain and what has made pain worse across the different domains in this pain interference figure using the Pain Balance Scale Worksheet (see Appendix D) together to identify their own experience across each category.

1. **Assess (15 min)**

**Pain story continued:**

- - How pain has affected their daily function (work, hobbies, family/friend interactions) – “What can you not do or do you do less because of your pain?”
  - Patient experience of and desire for treatment – “What treatment are you hoping to get that you think would help your pain?”
  - “What kind of treatment have you received so far for your pain?” Good areas of treatment to ask about include: medications, herbal medications, non-medication treatments (i.e. physical therapy, psychotherapy, yoga, diet, exercise, acupuncture, massage therapy, etc.); “Have any of them helped?”

1. **Optional: review measures (3–5 min):**

- The goal is to review PainTracker once every 6-8 weeks, but can address any significant changes (i.e., high scores on suicidal ideation, significant changes from the last time they filled it out that seem noteworthy to address)
  - **PainTracker:** review results with the patient if you notice changes from last visit that are important to address and ask about any concerns, they have that relate to the questionnaires they completed (see Appendix A)
  - Pointing out high scores can help open a conversation about recommendations to talk to their prescribing providers about psychotropic medications (i.e., antidepressants or other medications to address severe mental health related symptoms) or about a need to make a treatment plan to address severe or difficult symptoms (i.e., increase motivation to come to visits with the care manager and/or engaging treatment recommendations from providers)

1. **Set goals & care coordination (15 min):**
   - **Quality of Life Related Goals:** “If this program helped you over the next few months, what would you be able to notice is better?”
   - **Revisit what they’ve shared about what they think success of the program would look like for them.**
   - **Review answers on the worksheet and work through identifying specific targets (shape them into SMART goals) and then work with the patient to prioritize.**
     - Select a small target that they feel is doable to try to address before the next session. Make sure the goal is patient-centric and they feel it is doable. If they are feeling too overwhelmed to select something, let them know that is ok too and you will work with them more at the next visit to help figure out the best next steps. With CBT, we always want to emphasize there is no wrong answer or failure with any homework. We are here to help and support them.
   - **Common Barrier:** If the patient only talks about pain reduction as a goal (i.e., “my pain would be gone”), pivot to “Having less pain would be amazing, that makes great sense. If the physical pain didn’t change much, what do you think others could notice about you that would be different?” Or, you might say "I'm wondering if we can work on some skills now that can help you feel like you are doing more in areas that are important to you. Assuming your pain isn't going away tomorrow [or next week - offer a timeframe that is soon enough that isn't defeating by any stretch], you might learn how to live a little better with pain. And, if the pain does eventually go away, you'll be all the more prepared to live your best life."
     - The reality is that our study will most likely impact and improve function and enjoyment of life. However, studies have shown that patients are most likely to want to decrease pain intensity or to work on clarifying and getting a diagnosis. Furthermore, concern about pain intensity or pain diagnosis is generally rooted in not being able to complete daily tasks (function) and participate in hobbies/meaningful activities (enjoyment of life).
     - If the patient has indicated goals that focus on function or enjoyment of life, validate that choice and help decide if there are specific functional and/or enjoyment of life activities that the patient would like to focus on being able to do.
     - If the patient has indicated reducing pain intensity as their primary goal, ask questions of the patient to probe deeper about why pain intensity matters and what they would like to do if they were pain free or had less pain.
     - If the patient has indicated diagnosis as their primary goal, ask questions about what they are concerned about and why diagnosing the pain would be helpful. This sometimes can be a moment to help educate and/or return to the idea that we (you and their care team) will always prioritize a cure if we have one and at the same time, we also need to work on improving quality of life and function and reducing interference of pain in their life while they live without having a cure. Working might sound like “Absolutely, I understand the importance of getting some additional workups to figure out if there is something that might be explaining all of this. And, while that process is playing out, I'm wondering if we might work on some changes, you can make right now that can help you feel like you're making progress in some areas that matter, even with pain”,
     - Discussing goals of treatment is often an ongoing conversation across multiple sessions, especially if patients are less ready to change or struggling with other barriers to engaging in working on pain self-management that occur in CBT 1-2-3 Step 2.
   - **Social needs:**
     - Determine/follow up on referrals from last session and goals related to addressing social needs
   - **Care Coordination:**
     - Identify and follow up on goals of care as needed, set concrete steps/actions if needed
     - Facilitate communication between specialists, primary care, other pain treatment providers appropriate to your care team (e.g., can be through messaging to other providers electronically, talking to providers directly, or coaching the patient on plans for communication at their next provider visit)
     - If there were any missed appointments for pain related reasons, elicit the reason for missing the appointment, troubleshoot avoiding future appointments and ask if they need help facilitating rescheduling of the appointment
     - Inquire if they had urgent care or emergency room visits related to pain and see if there are ways to troubleshoot avoiding these visits in the future. One way to do this is to ask the patient what their primary goal was for the visit to the emergency room or urgent care and to see if those goals might be met through other actions.
     - Determine if care is going smoothly and determine if they need help with engaging with providers or accessing / managing their care plan
   - **Virtual Exercise Program: Tele-EF**
     - Check in and see if they want to get registered and started in Tele-EF. If you would like to refer a patient to Tele-EF, please use the link provided on [the study website](http://aim-cp.uw.edu/). Select the participants time zone and then review the list of available Tele-EF classes with them. The website will show a list of offerings, including the day of the week and the time at which the class is taught, and where the class is taught from. Participants may choose to attend a class regardless of where it is taught from. When the participant selects a class, please select "Refer". This will guide you to a contact information form which will then be sent directly to the Tele-EF instructor, or to staff that manage enrollment. The instructor or staff will follow up with the participant to complete the Tele-EF enrollment process.
   - **Summarize plan:** Give brief synopsis of next steps and any homework to be done before the next visit
2. **Set next appointment**
   - Plan to meet in two weeks for one hour via audiovisual conferencing (i.e. Zoom) or phone.
3. **Complete patient session/visit note in Acuity**
   - You can use the template in Appendix B to document in the EHR. The note in the EHR can be copy and pasted into the open answer field in Acuity. Please also check the areas that were addressed during this visit in Acuity.

# Session 3 (Step 2: Grow)

**(Begin CBT 1-2-3 Step 2: Grow)**

1. **Set agenda**
   - Review homework and updates (“let’s catchup”), address care coordination, finish goal setting if needed, start CBT 1-2-3 intervention by educating about the pain model using the 3 Bs. During this session, you will be determining if treatment can start with selecting a pain self-management treatment target OR if a barrier must be addressed first (i.e., CBT 1-2-3 Step 3 is needed, in which case jump to instructions for Step 3/Session 4)
2. **Review homework and measures (5–10 min)**
   - Review homework progress and remember to validate any effort and reiterate that they cannot fail at homework, we are always happy to hear what got in the way and help problem solve
   - **PainTracker:** review results with the patient if you notice changes from last visit that are important to address and ask about any concerns, they have that relate to the questionnaires they completed (see Appendix A)
3. **Deliver CBT 1-2-3 Intervention (30 min)**
   - **Introduce CBT 1-2-3:** “Now that we’ve had a chance to get going, I’d like to start working on some of the self-management tools with you to start helping things go a little better. Remember the model of chronic pain we reviewed, with all the things that can make pain better or worse? Now we are going to start to focus on these part by part, so you can learn more about how pain works in the brain, and how to make it better.”
   - **Educate:** 3Bs. Bring the patient’s attention to the work you did last week on the models of pain, and what has worked and has not worked for them. Now, introduce CBT using the 3B’s for Pain Worksheet (see Appendix E). “*Cognitive Behavioral Therapy, or CBT, addresses the main areas we talked about last week, that get affected by chronic pain. I’d like to go through this together and we can fill in some of the ways this works for you. First, there is the body. Now, this one isn’t just the pain itself - it is the way our body reacts to the pain, or the emotions we feel about pain. Some people feel heavy and down or might cry or feel tired a lot; some might be tense and anxious and notice their heart pounds when they feel a twinge of pain, or that they are always tense. What do you feel when you’re in pain? Second, the brain gets involved and thinks in certain ways. We think about how bad things are, and it gets hard to stay in the moment. What do you notice going through your mind? Third, we behave in certain ways. We slow down, don’t pace ourselves, and stop doing things we value a lot sometimes. Any places you relate to this? Good. Now, the CBT model intervenes in these very areas. So, we’ll teach ways to help your body, your thinking brain, and help you change behavior in ways that will help you manage your pain.” (now show the same model with tools – see figure in Appendix E)*
     - **Pain Self-Management:** The most powerful way to start is by changing behavior, so if the patient is up for it, we recommend you start there. You could start with movement (and / or refer to the exercise program), paced activity, or valued activity. Otherwise, you could start with the body and teach breathing / relaxation or move to the thinking skills and start teaching either mindfulness or calm thinking skills (see Appendix E).
   - **OR Assess and address barriers to CBT 1-2-3 Step 2**
     - Common barriers include: low readiness to change that is often due to difficulty with acceptance of chronic pain (see Figure for Stage 1 on readiness to change); if acceptance is low (i.e., patients sometimes feel they can’t set or reach goals until the pain is gone – “I can’t do the things I love until the pain goes away!” “I can’t live like this, they need to just finally figure out what’s wrong so I can get the right treatment.”), it can help to reflect back their experience and then say “My goal is to help you live the best quality of life you can, even though you haven’t figured out a way to cure your pain or make it manageable. What I mean is, let’s do both. Let’s make sure we’re doing everything we can to find a cure and at the same time, work on making your life better while you still hurt today.” Sometimes that reframe can help people both feel heard and move forward with pain self-management goals.
     - If barriers include moderate to severe depression, anxiety, posttraumatic stress, opioid use problems, or crises, jump to Session 4.
4. **Care Coordination**
   - **Social needs:**
     - Determine/follow up on referrals from last session and goals related to addressing social needs
   - **Discuss other care for pain:**
     - Have they seen any specialists or participated in any non-health care activities to improve pain? Did they miss any appointments and if so why and how can these be rescheduled?
     - Determine if care is going smoothly and determine if they need help with engaging with providers or accessing / managing their care plan
     - Facilitate communication between specialists, primary care, other pain treatment providers appropriate to your care team (e.g., can be through messaging to other providers electronically, talking to providers directly, or coaching the patient on plans for communication at their next provider visit)
     - Have they been to the Emergency Department or urgent care for a pain related reason? Discuss goal for their visit and potential ways to avoid these visits (if appropriate).
5. **Tele-EF**
   - If not yet participating in Tele-EF: check in and see if they want to get registered and started in Tele-EF. See section in Session 2 about this if they are ready to get started
   - If currently participating in Tele-EF, check to see how it is going and help address any barriers to regular attendance
6. **Summarize plan**
   - Give brief synopsis of next steps and any homework to be done before the next visit
7. **Set next appointment**
   - Plan to meet in two weeks for one hour via audiovisual conferencing (i.e. Zoom) or phone
8. **Complete patient session/visit note in Acuity**
   - You can use the template in Appendix B to document in the EHR. The note in the EHR can be copy and pasted into the open answer field in Acuity. Please also check the areas that were addressed during this visit in Acuity.

**If the patient can start the CBT basic tools, you will move through them until they have some success with the Behavior tools, the Brain thinking tools, and the Body tools. If this is successful, they may complete the CBT program having only done Step 2. If they are experiencing barriers such as depression, anxiety, trauma, or opioid use problems and it is making it difficult to engage with the CBT 1-2-3 Step 2, you can move to Step 3 until if/when they are ready to return to Step 2.**

# Session 4 (Step 3: Bend, Don’t Break OR Step 2: Grow)

**(Address Barriers with CBT 1-2-3 Step 3 / *Bend, Don’t Break*) OR continue CBT 1-2-3 Step 2 (*Grow*)**

1. **Set agenda**
   - Review homework and updates (“let’s catchup”), address care coordination, assess goal for addressing barriers to CBT 1-2-3 Step 2, start CBT 1-2-3 intervention for barrier
2. **Review homework and measures (5–10 min)**
   - Review homework progress and remember to validate any effort and reiterate that they cannot fail at homework, we are always happy to hear what got in the way and help problem solve
   - PainTracker: (if at monthly check-in) review results with the patient if you notice changes from last visit that are important to address and ask about any concerns, they have that relate to the questionnaires they completed (see Appendix A)
3. **Deliver CBT 1-2-3 interventions for barriers (30 min)**
   - **Determine barrier priority:** Work with patient and review measures to determine which barrier is primary to the patient
   - **Often it can be best to start with depression or anxiety,** whichever is worse and/or primary or top priority to the patient – if you are not sure, you can ask the patient “Do you think it would be better if we worked on your depression or your anxiety as the higher priority?” or “Do you think your depression or anxiety is getting in the way more of you using the strategies to cope with your pain?”
     - Use CBT techniques explained in the asynchronous Modules 8 & 9 for depression and anxiety, respectively
   - **Trauma:** Some patients will have trauma cues that are clearly impacting their ability to engage in the CBT 1-2-3 Step 2. If this is the case, you can move to addressing the trauma with them for one or two sessions. After that, a referral may be required to a higher level of care.
     - The following are different approaches you can take for one or two sessions to help people manage posttraumatic stress symptoms:
       - **Narrative writing:** Teaching patients about the common reactions to trauma and the importance of processing trauma memories so that they can gradually become less intense and overwhelming can be helpful. Ask the patients to write for 20 minutes over five sessions, exploring their thoughts and feelings about the event. They may write about what they would tell a friend who was going through the same thing, how the event has changed them, and include details if they wish. This practice over a few weeks can help the patient with exposure to their traumas that can lead to reducing posttraumatic stress. However, writing about trauma experiences at first can make things worse. So, make sure the patient can tolerate symptoms getting worse at first and you help with a plan to cope with symptoms first if needed.
       - **Cognitive therapy:** Teach thought challenging skills, similarly to those in CBT 1-2-3 Step 2, with an emphasis on working on challenging negative thoughts about self-blame related to the traumatic experiences and about themselves. The focus can also be on growing self-compassion.
       - **Grounding:** Patients sometimes dissociate due to past trauma, where they feel like they are out of body or on the outside looking in. Having patients focus using grounding skills that can be as simple as teaching them to name five things they see in the room or name one thing they experience from each of their five senses (you can also find more ideas here): <https://www.apa.org/topics/stress/manage-stress-tools>). Grounding often stops dissociation and if used, a patient can reduce their dissociative symptoms.
       - **Relaxation and sleep hygiene:** Use the same skills from CBT 1-2-3 Step 2
       - **Medication:** psychotropic medications, such as anti-depressants, can help reduce core symptoms of posttraumatic stress disorder and allow for more exposure-based interventions like narrative writing. If the patient is taking benzodiazepines, it can be helpful to make sure these are scheduled and not taken at the peak of anxiety, but beforehand is best.
   - **Opioid use problems:** Some patients will have opiate use problems that are clearly impacting their ability to engage in the CBT-Step 2. If this is the case, you can move to addressing the opiate use with them for one or two sessions. After that, a referral may be required to a higher level of care.
   - **Crisis:** If the patient is voicing being in crisis (e.g., experiencing an extreme moment when they have a situation that reminds them of a past trauma), use distress tolerance skills to help them move through the crisis more effectively (see Module 10).
   - **Summarize plan:** Give brief synopsis of next steps and any homework to be done before the next visit.
4. **Care coordination**
   - **Social needs:**
     - Determine/follow up on referrals from last session and goals related to addressing social needs
   - **Discuss other care for pain:**
     - Have they seen any specialists or participated in any non-health care activities to improve pain?
     - Determine if care is going smoothly and determine if they need help with engaging with providers or accessing / managing their care plan
     - Facilitate communication between specialists, primary care, other pain treatment providers appropriate to your care team (e.g., can be through messaging to other providers electronically, talking to providers directly, or coaching the patient on plans for communication at their next provider visit)
     - Have they been to the Emergency Department or urgent care for a pain related reason? Discuss goal for their visit and potential ways to avoid these visits (if appropriate).
5. **Tele-EF**
   - If not yet participating in Tele-EF: check in and see if they want to get registered and started in Tele-EF. See section in Session 2 about this if they are ready to get started
   - If currently participating in Tele-EF, check to see how it is going and help address any barriers to regular attendance
6. **Set next appointment**
   - Plan to meet in two weeks for one hour via audiovisual conferencing (i.e. Zoom) or phone.
7. **Complete patient session/visit note in Acuity**
   - You can use the template in Appendix B to document in the EHR. The note in the EHR can be copy and pasted into the open answer field in Acuity. Please also check the areas that were addressed during this visit in Acuity.

# Session 5 and onward (Deliver CBT 1-2-3 and Care Management)

1. **Set agenda**
   - Review homework and updates (“let’s catchup”), address care coordination, continue with CBT 1-2-3 intervention
2. **Review homework and measures (5–10 min)**
   - Review homework progress and remember to validate any effort and reiterate that they cannot fail at homework, we are always happy to hear what got in the way and help problem solve
   - **PainTracker:** review results with the patient if you notice changes from last visit that are important to address and ask about any concerns, they have that relate to the questionnaires they completed (see Appendix A)
3. **Deliver CBT 1-2-3 intervention (30 min)**
   - **Continue with CBT 1-2-3 Step 2 or Step 3 interventions if appropriate**
   - **Define and prioritize a new intervention target as goals are reached**
   - **Summarize plan:** Give brief synopsis of next steps and any homework to be done before the next visit
4. **Care coordination:**
   - **Social needs:**
     - Determine/follow up on referrals from last session and goals related to addressing social needs
   - **Discuss other care for pain:**
     - Have they seen any specialists or participated in any non-health care activities to improve pain?
     - Determine if care is going smoothly and determine if they need help with engaging with providers or accessing / managing their care plan
     - Facilitate communication between specialists, primary care, other pain treatment providers appropriate to your care team (e.g., can be through messaging to other providers electronically, talking to providers directly, or coaching the patient on plans for communication at their next provider visit)
     - Have they been to the Emergency Department or urgent care for a pain related reason? Discuss goal for their visit and potential ways to avoid these visits (if appropriate).
5. **Tele-EF**
   - If not yet participating in Tele-EF: check in and see if they want to get registered and started in Tele-EF. See section in Session 2 about this if they are ready to get started
   - If currently participating in Tele-EF, check to see how it is going and help address any barriers to regular attendance
6. **Set next appointment**
   - Plan to meet in two weeks for one hour via audiovisual conferencing (i.e. Zoom) or phone.
7. **Complete patient session/visit note in Acuity**
   - You can use the template in Appendix B to document in the EHR. The note in the EHR can be copy and pasted into the open answer field in Acuity. Please also check the areas that were addressed during this visit in Acuity.

# Final Session (Relapse Prevention and Terminate Care Management and CBT 1-2-3 Care)

1. **Set agenda**
   - Review homework and updates (“let’s catchup”), address care coordination, recap progress, review skills, review warning signs for when to return for care, refer out if needed
2. **Review homework and measures (5–10 min)**
   - Review homework progress and remember to validate any effort and reiterate that they cannot fail at homework, we are always happy to hear what got in the way and help problem solve
   - PainTracker: review results with the patient if you notice changes from last visit that are important to address and ask about any concerns, they have that relate to the questionnaires they completed (see Appendix A)
3. **Review treatment progress (10–15 min): use Relapse Prevention worksheet (see Appendix C)**
   - **Summarize progress on measures**
   - **Summarize skills learned**
4. **Review Relapse Prevention plan (10–15 min): see Module 7 webinar**
   - **Summarize plan:** Give brief synopsis of when to reach out for care and how
5. **Complete patient session/visit note in Acuity**
   - You can use the template in Appendix B to document in the EHR. The note in the EHR can be copy and pasted into the open answer field in Acuity. Please also check the areas that were addressed during this visit in Acuity.

# Asynchronous Training Component: Description of Modules

- **Module 1: Chronic Pain Basics**

[[Link to video here]](https://uw.hosted.panopto.com/Panopto/Pages/Viewer.aspx?id=75e00f66-f744-46eb-9f96-b13e01020ef1)

In this session, we discussed the basic principles of chronic pain. Chronic pain is often defined as pain that lasts for more than three to six months. It can be continuous or episodic and may range from mild to severe. The principles we covered are ranges of symptoms, impact on one’s life, treatment options, self-management strategies, using a multidisciplinary approach, patient education, and offering supportive care.

Understanding the basics of chronic pain is essential for healthcare professionals and individuals affected by this condition to effectively manage symptoms and improve quality of life.

- **Module 2: Chronic Pain Self-Management**

[[Link to video here]](https://uw.hosted.panopto.com/Panopto/Pages/Viewer.aspx?id=45abf15e-90b6-4057-8722-b13e0102d368)

In this session, we cover strategies used to manage chronic pain. These strategies include pain tracking, regular physical activity, pacing activities, stress management, adopting healthy lifestyles, building social support, relaxation techniques, and goal setting.

Incorporating these strategies empowers individuals to actively participate in their care, optimize treatment outcomes, and enhance their overall quality of life

- **Module 3: Working as a team: Best Practices for Communicating and Coordinating Care**

[[Link to video here]](https://uw.hosted.panopto.com/Panopto/Pages/Viewer.aspx?id=5f4cef6a-483f-4f2c-8831-b13b016bdc51)

In this session, we discuss best practices for ensuring effective communication and coordination of care with care providers and patients. These practices include sharing goals, building mutual trust with each other, looking for opportunities to build skills, and clarifying roles and how the team will operate to ensure expectations are being met.

- **Module 4: Structuring Your Visit**

[[Link to video here]](https://uw.hosted.panopto.com/Panopto/Pages/Viewer.aspx?id=c5e8ba7c-1ea8-444c-8c45-b13b016d4793)

In this session, we discuss the steps needed to complete while creating a meeting agenda for your patients. Steps to complete before meeting with the patient include reviewing the patient's medical record and any medical or behavioral health notes. During the visit, steps include creating an agenda, checking in on any symptoms patient may have, checking to see if medication is up to date, reviewing any previous discussed goals/plans, reviewing content of current session, and making plans for the next session. After meeting with a patient, always be sure to complete charting in the electronic health record and registry, follow up with care team, if needed, and review case and prepare for case consultation.

- **Module 5: Medication Management**

[[Link to video here]](https://uw.hosted.panopto.com/Panopto/Pages/Viewer.aspx?id=432524e3-39df-49a9-884a-b13e01020f11)

In this session, we discuss medication management as it plays a significant role in treating chronic pain, depression, and anxiety, often involving the use of psychotropic medications. These medications aim to alleviate symptoms and improve overall functioning, but they can also carry potential side effects that need to be carefully monitored.

Effective medication management involves close collaboration between patients and healthcare providers to monitor treatment response, adjust dosages as needed, and address any side effects or concerns. It is essential for individuals to be informed about the potential benefits and risks of psychotropic medications and to actively participate in treatment decisions to optimize their overall health and well-being.

- **Module 6: Safety Planning**

[[Link to video here]](https://uw.hosted.panopto.com/Panopto/Pages/Viewer.aspx?id=3f79bfde-7ed3-4938-b322-b13e01020f23)

In this session, safety planning is a critical tool in managing suicidal ideation and psychiatric emergencies. It involves creating a personalized plan to help individuals navigate crises and stay safe during moments of heightened distress. A safety plan includes identifying triggers and warning signs, coping strategies to manage distress, and a list of supportive contacts or resources to reach out to for help. Additionally, it may involve removing access to unhealthy means and developing a step-by-step plan to follow during a crisis. By proactively addressing risk factors and establishing coping mechanisms, safety planning empowers individuals to effectively manage suicidal thoughts and emergencies while promoting their overall well-being and safety.

- **Module 7: Relapse Prevention**

[[Link to video here]](https://uw.hosted.panopto.com/Panopto/Pages/Viewer.aspx?id=b514b80a-b812-4752-a12a-b13e01020f0e)

In this session, relapse prevention is a crucial aspect of maintaining progress in recovery from various mental health or addictive disorders. It involves identifying triggers, developing coping strategies, and creating a plan to prevent setbacks or lapses. By recognizing early warning signs and implementing effective coping skills, individuals can minimize the risk of relapse and sustain long-term recovery. Relapse prevention strategies mentioned include stress management techniques, building a strong support network, and maintaining healthy habits. Overall, relapse prevention aims to empower individuals with the tools and skills needed to navigate challenges and sustain positive changes in their lives.

*This session also provides a relapse prevention plan worksheet found in Appendix C.*

- **Module 8: CBT for Depression**

[[Link to video here]](https://uw.hosted.panopto.com/Panopto/Pages/Viewer.aspx?id=33e1b6c9-d104-48e3-b9f6-b142014a4268)

In this session, CBT for depression targets negative thought patterns and behaviors. Therapists help individuals challenge negative thoughts and engage in enjoyable activities. Techniques like cognitive restructuring and behavioral activation are used to replace negative beliefs and increase positive behaviors. Additionally, CBT for depression may incorporate other techniques such as problem-solving skills training, assertiveness training, and relaxation techniques to help individuals develop coping strategies and enhance their ability to manage stressors and depressive symptoms. It's effective in alleviating depression by empowering individuals with practical coping strategies and improving their overall well-being.

- **Module 9: CBT for Anxiety**

[[Link to video here]](https://uw.hosted.panopto.com/Panopto/Pages/Viewer.aspx?id=4cd1e07b-c9b6-4447-8064-b144004830a2)

CBT for anxiety is an effective therapeutic approach that targets the cognitive and behavioral aspects of anxiety disorders. It operates on the premise that our thoughts, feelings, and behaviors are interconnected, and by changing dysfunctional thought patterns and maladaptive behaviors, individuals can alleviate their anxiety symptoms.

In this session, we examine how therapist and client work collaboratively to identify and challenge irrational or negative thoughts that contribute to anxiety. This process often involves techniques such as cognitive restructuring, where individuals learn to replace irrational thoughts with more realistic and adaptive ones. Overall, CBT for anxiety offers a practical and evidence-based approach to managing anxiety symptoms by targeting both cognitive distortions and maladaptive behaviors, empowering individuals to regain control over their lives and effectively cope with anxiety-related challenges

- **Module 10: Distress Tolerance for Chronic Pain Flares and Suicidality**

[[Link to video here]](https://uw.hosted.panopto.com/Panopto/Pages/Viewer.aspx?id=7095fae7-99a6-4b46-82b1-b140016b020a)

This training session focused on applying Dialectical Behavior Therapy (DBT) techniques to manage crises, particularly in chronic pain patients. Distress tolerance skills are aimed at reducing intense stress and improving overall functioning during crises. A crisis is defined as a situation where a serious problem cannot be immediately solved, leading to high emotions and distress. For chronic pain patients, pain flares can constitute a crisis, necessitating the application of distress tolerance skills.

Overall, the training aimed to equip participants with the knowledge and tools to effectively integrate distress tolerance skills into their clinical practice, fostering resilience and adaptive coping in patients facing chronic pain and emotional distress.

# Appendix A: PainTracker Measures

[(click here for a printable copy)](https://depts.washington.edu/aim-cp/wordpress/wp-content/uploads/2025/08/PainTracker-Measures.pdf)

| Questionnaire / Measure | Description | Baseline | Monthly |
| --- | --- | --- | --- |
| Goal | This multiple-choice question asks patients to rank their top 3 goals for their pain. It will be used to inform targets for CBT. | X |  |
| PROMIS-29 Pain Interference | PROMIS Pain Interference measures how much pain interferes with a patient's daily physical and social activities. Scores range from 41.6–75.6 with higher values indicating greater (worse) pain interference with daily activities. On this scale, an absolute change of 2.5 or more is clinically significant.  For example, if a patient goes from 57.1 at one visit to 59.9 at the next visit, then the impact of this patient’s pain on their life is getting worse and is clinically significant. In contrast, if a patient’s score goes from 59.9–57.1, then their pain is getting better and they are experiencing significantly less pain interference with their daily activities. | X | X |
| Sleep Interference | These questions ask how much pain has interfered with sleep in the past week. | X |  |
| Activity Interference | These two questions ask about how pain has interfered with important activities for the patient. This can be used as a target in CBT. | X |  |
| Pain Location | Patients are provided diagrams of the front and back of the body and are asked to highlight areas in the body where they are experiencing pain. | X |  |
| Physical Activity Vital Sign | The Physical Activity Vital Sign consists of four questions that quantify how many days a week and how many minutes in those days patients engage in (a) moderate or vigorous exercise and (b) muscle strengthening exercises. Moderate exercise is defined as activities where patients can talk but not sing (e.g. brisk walking, slow biking, active home chores) while vigorous exercise is defined as activities where patients cannot talk easily and are somewhat out of breath (e.g. jogging, fast biking, swimming, aerobic exercise).  Patients are recommended to perform moderate or vigorous exercise for at least 150 minutes per week. Consider referring patients who are engaging in less than 150 minutes of exercise per week to Tele-EF. | X |  |
| PHQ-9 | PHQ-9 is used to assess for depressive symptoms. This can be used to decide how much depression is affecting patients’ engagement in their care. For patients with moderate or severe depression, CBT targets should include depression modules.  Question #9 in the PHQ-9 asks about suicidal thoughts. Please use the suicide ideation workflow if a patient screens positive for any number on this question.  Score interpretation is as follows:   - 0–4: absence of depression - 5–9: mild depression - 10–14: moderate depression - 15+: major depression | X | X |
| GAD-7 | GAD-7 is used to assess for anxiety symptoms. This can be used to decide how much anxiety is affecting patients’ engagement in their care. For patients with moderate or severe anxiety, CBT targets should include anxiety module.  Score interpretation is as follows:   - 0–4: absence of anxiety - 5–9: mild anxiety - 10–14: moderate anxiety - 15+: severe anxiety | X | X |
| Medication Side Effects | This question asks about any side effects that patients may have from their medications. | X |  |
| Treatment Satisfaction | Patients are asked to rank on a scale of 0–10 their satisfaction with their current pain treatment. This can be useful for care coordination to see if other targets need to be considered and for CBT to help patients redirect expectations/goals. | X |  |
| Current Treatment | This question asks about any current treatments that are non-medication-based that patients may be currently receiving for their pain. It can be used to inform their pain story and what patients have tried for pain. | X |  |
| PC-PTSD-5 | The Primary Care PTSD screener consists of 5 questions where patients are asked to answer yes/no to reactions or behaviors in the past month to previous trauma. The scale is scored from 0-5 and a score of 3 or greater is considered a positive screen. Individuals scoring high on this scale should receive PTSD module as part of their CBT treatment. | X |  |
| TAPS | TAPS asks about substance use and is broken down into TAPS-1 and TAPS-2. All patients are asked the four questions in TAPS-1 about tobacco, alcohol, prescription misuse and other substance use. Options are never, less than monthly, monthly, weekly or daily. If they score positive on any of these questions, they answer more detailed questions about that category of substance use in TAPS-2. Answers to this question can be used to determine if patients need medications for substance use disorder (refer to PCP or to specialist) or specific modules on substance use in their CBT.  For the TAPS-1, scores of monthly or more frequent for tobacco and alcohol are considered problematic. Scores of any use (less than monthly or more frequent) for prescription drugs and illicit drugs are considered problematic. | X |  |

# Appendix B: EHR Documentation Template

[(click here for a printable copy)](https://depts.washington.edu/aim-cp/wordpress/wp-content/uploads/2025/08/EHR-Documentation-Template.pdf)

**Initial Note**

*(retain elements that apply/delete elements that do not and add fill in the blank)*Patient’s pain history: <summarize patient’s pain history>

Social needs: <list any positives>

PainTracker: <list any positive or concerning elements>

Goals of care:

- Patient’s initial goals as described in PainTracker: ___
- We discussed together to clarify goals of care and patient aims to: ____

Plan:

- Introduced components of the care management program including care coordination, CBT and Tele-EF
- Patient will focus on following for next visit: ______
- Plan for discussing the following at next visit: ______
- Follow-up with patient in __ weeks

**Follow-up Notes**

*(retain elements that apply/delete elements that do not and add fill in the blank)*

Review:

- Reviewed goals of care which included: ___
- Social needs addressed: _____
- PainTracker reviewed with patient: There was improvement in ___ and lack of improvement in ___.
- The following elements of CBT were used with patient today: ____
- Most of the session was spent on ____
- I believe the following is most likely to be helpful to the patient _______
- Patient has engaged in the following treatments since the last visit: _______

Plan:

**Example communication with patient’s PCP**

*(add or delete from below communication template as desired)*

Dear ____,

Your patient, ________, is participating in a research study where they will engage in nurse care management for their chronic pain. As part of the study, they will receive CBT, care coordination and potential referrals to a virtual exercise program (if clinically indicated). I will be helping provide this care as their care manager over the next six months. Please let me know if you have any questions about this program or any clinically relevant information, I should be aware of. I will be providing occasional updates to you.

Thanks!

# Appendix C: Relapse Prevention Plan Worksheet

[(click here for a printable copy)](https://depts.washington.edu/aim-cp/wordpress/wp-content/uploads/2025/08/Relapse-Prevention-Plan-Worksheet.pdf)

Date:

Purpose: It is common for pain and stress to recur numerous times in one’s life. The purpose of a relapse prevention plan is to understand your own personal warning signs that let you know when symptoms may be returning, so that you can reach out and get help early on before symptoms get worse. It also helps to remind you of strategies and skills that work to help you feel better.

Possible signs that I am beginning to struggle / may be decreasing my skills practice:

1.

2.

3.

My ongoing personal plan – things that help me feel better:

1.

2.

3.

If symptoms return, contact: ________________________________

Maintenance Medication (if applicable)

1. __________________; tablets____ of _______mg take at least until _____________

2. __________________; tablets____ of _______mg take at least until _____________

3. __________________; tablets____ of _______mg take at least until _____________

Call your treating health care provider with any questions.

Continued Care goals

1.

2.

3.

Health Care Provider: ___________________ Phone: ____________________ Email:

Care Manager: _________________________Phone: _____________________Email:

Next appointment: Date: Time:

# Appendix D: Scale Worksheet

[(click here for a printable copy)](https://depts.washington.edu/aim-cp/wordpress/wp-content/uploads/2024/09/Scale-Worksheet.pdf)


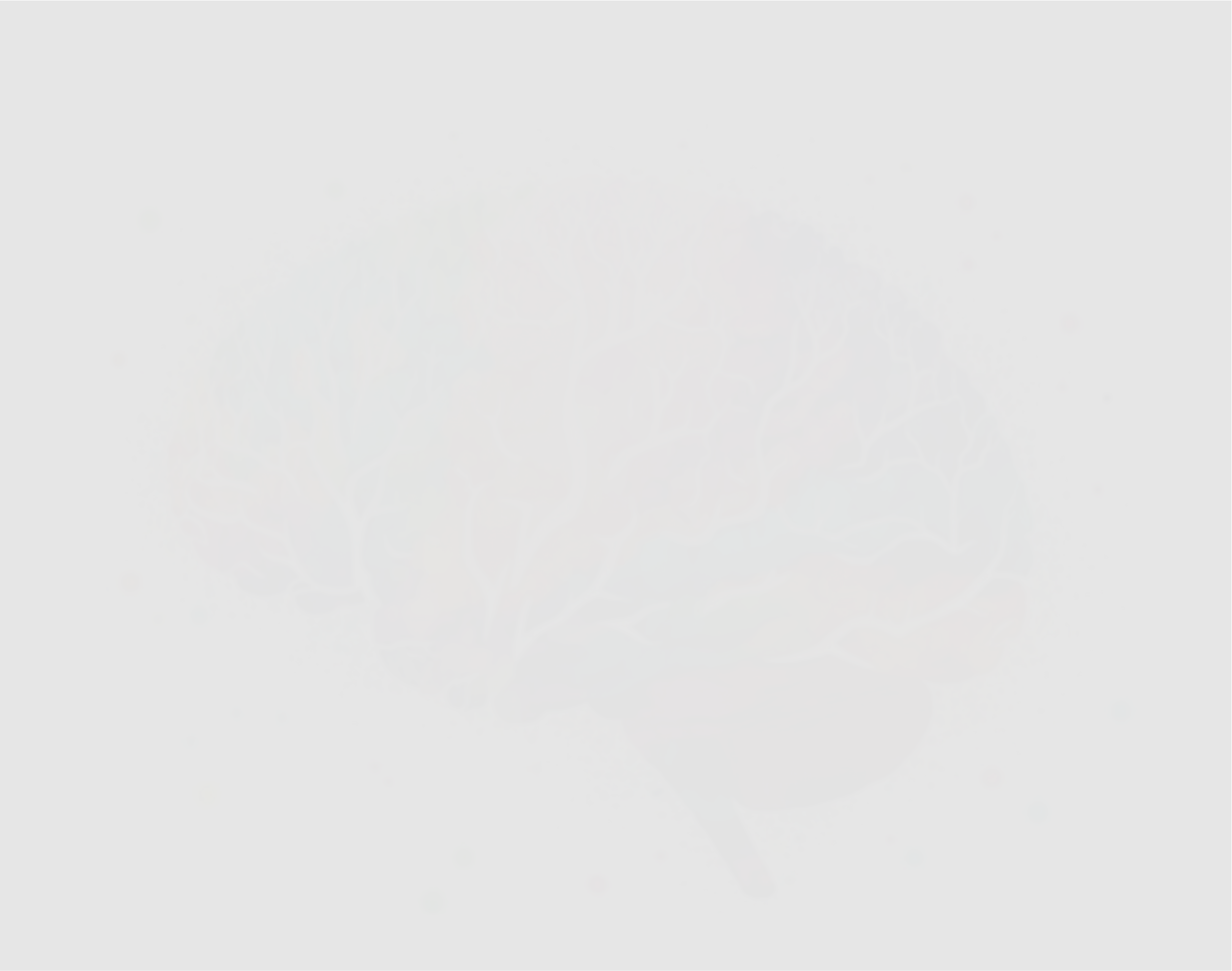


TREATING CHRONIC PAIN = QUALITY OF LIFE

**Less**

MEMORIES

EMOTIONS
BELIEFS

SOCIAL NETWORK

**More**

ENVIRONMENT

STRESS

SLEEP

EXERCISE

DIET

MEDICAL ADVICE

HEALTH PROFILE

IMMUNE SYSTEM

Let’s identify how these diﬀerent areas either help or hurt pain for you:


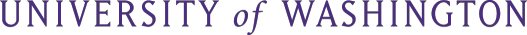

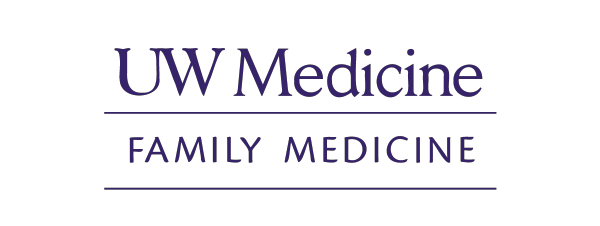


PAIN INTERFERENCE

Helps/Less Pain Hurts/More Pain

| *Emotions* |  |  |
| --- | --- | --- |
| *Beliefs Memories Social Support Environment*  *Stress Sleep Exercise*  *Diet Medical Advice Health Proﬁle*  *Immune*  *System* |  |  |
|  |  |  |
|  |  |  |
|  |  |  |
|  |  |  |
|  |  |  |
|  |  |  |
|  |  |  |
|  |  |  |
|  |  |  |
|  |  |  |

# Appendix E: 3B’s for Pain Worksheet

[(click here for a printable copy)](https://depts.washington.edu/aim-cp/wordpress/wp-content/uploads/2024/09/3Bs-for-Pain-Worksheet.pdf)

What do you notice happens in your body, brain, and with your behavior when you have chronic pain?


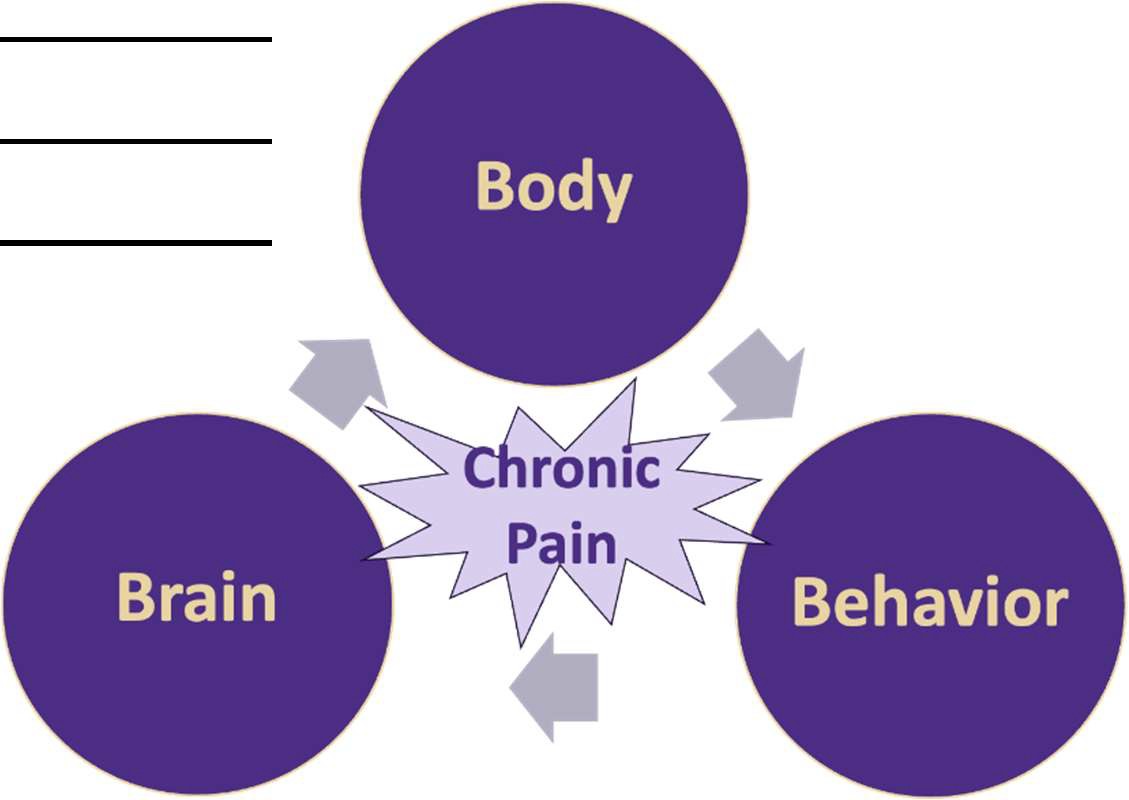


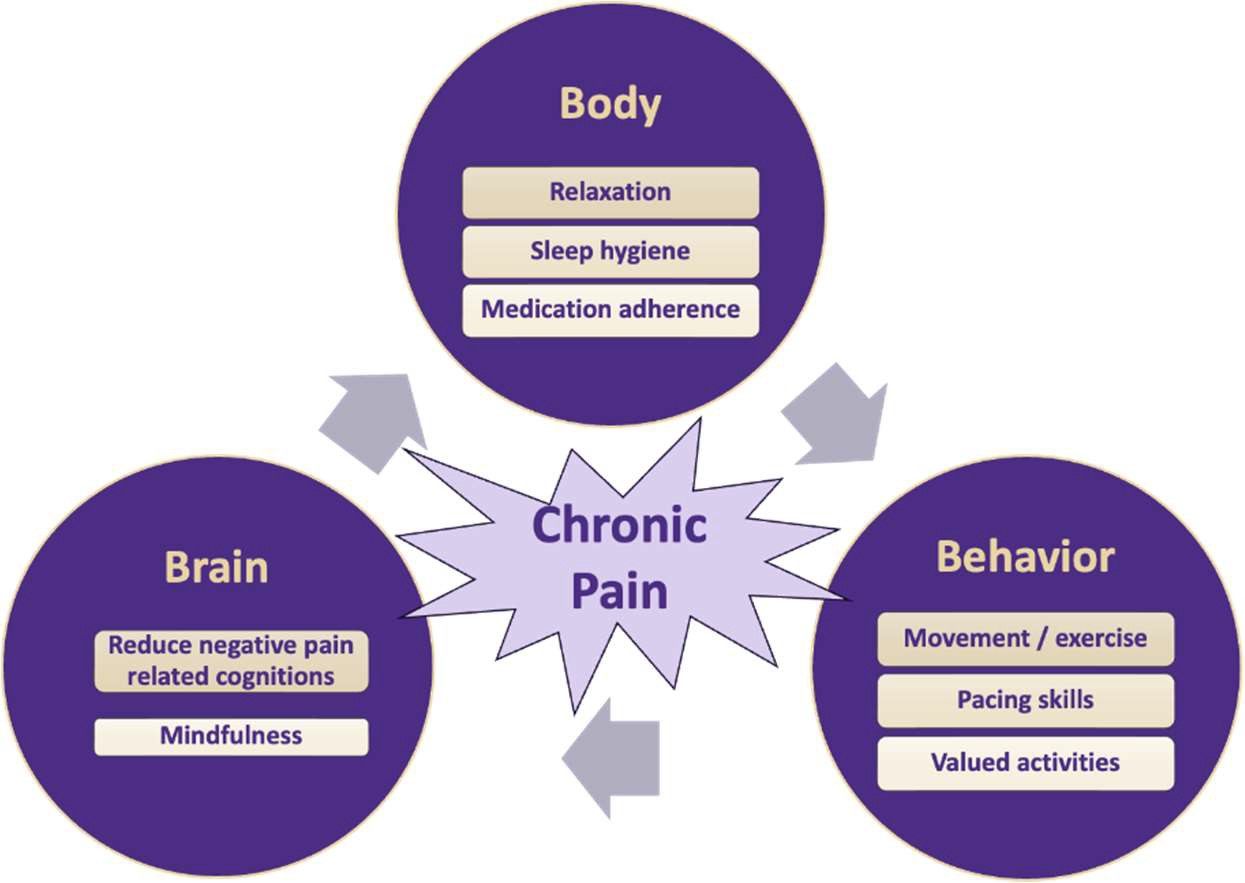


# Appendix F: Behavior Skills for Chronic Pain

**Body**

**Brain**

**Behavior**

**Chronic**

**pain**

[(click here for a printable copy)](https://depts.washington.edu/aim-cp/wordpress/wp-content/uploads/2025/10/SummaryBehaviorSkills.pdf)

**Movement**:

The first behavior to encourage patients to change is movement. Put simply, “motion is lotion.” Anything that is cleared by their care team is possible, from walking out to get the mail from the mailbox outside to walking up to the TV to turn it on and off. If the patient is ready, they can be referred to Tele-EF. Otherwise, think about small and steady steps. Some patients will be concerned about the possible pain / damage to their bodies if they begin moving. Here is a helpful video: <https://youtu.be/L6IQRo1y_L4>.

**Pacing:**

Many patients with chronic pain struggle with an all-or-nothing approach to movement and activity. One day, they might feel good, do a lot and find themselves wiped out and exhausted. After that, they might stay in bed or do very little for several days, and the cycle continues. The solution is pacing, where the patient begins to do a scheduled amount of activity, regardless of pain, and pace it moderately / increase it (if desired) in a slow and methodical way. A good rule of thumb is to take the average of three days, subtract 20%, and then increase that amount of activity by 10% as desired. Slow and steady wins the race here.

**Values**

The last and most important part of the behavior module is helping the patient identify what comprises a life worth living for them and beginning to engage in things that are important to them. Pain can be a barrier, and effective self-management of pain involves finding ways to overcome those barriers and re-engage in activities that the patient values.

# Appendix G: Activity Pacing

**Body**

**Brain**

**Behavior**

**Chronic**

**pain**

[(click here for a printable copy)](https://depts.washington.edu/aim-cp/wordpress/wp-content/uploads/2025/10/ActivityPacing.pdf)

**The Boom-and-Bust Pattern of Chronic Pain:**

- This refers to a common pattern people find themselves in. When you have chronic pain, it is tempting to push yourself to do lots of physical activity or a strenuous task when you are having a “good” day. Later, your pain becomes severe, and you have to rest for a long time to recover.
- This cycle tends to repeat over and over.
- In the long run, staying in this pattern will make pain worse and people find themselves avoiding things that are fun and make them feel good, so that they do not get knocked off their feet.

Activity Pacing:

- The key here is to engage in a moderate and safe level of activity on a regular basis (consistently), so that you can avoid being overactive or underactive.
- Pacing is about planning and balancing active vs. rest time! Try the following:

1. Make a list of some of the things that you tend to overdo.

2. Make a time limit for the activity and then STOP and REST.

3. Keep track of how you are doing and adjust accordingly.

|  | Example:  Talk with friend on phone | Activity 1 | Activity 2 |
| --- | --- | --- | --- |
| Active time goal | 15 min |  |  |
| Rest time goal | 15 min |  |  |
| Day 1 | 15:15 (2 cycles, fine) |  |  |
| Day 2 | 20:15 (1 cycle, tired) |  |  |
| Day 3 | 10:15 (1 cycle, better) |  |  |
| Day 4 (to 7) | 10:15 (2 cycles, good) |  |  |
| Result | 10:15 ratio works best |  |  |

The goal of pacing is to gradually increase your activity levels that are near normal as possible on most days. This can allow you to accomplish things that you want to do. Learning pacing techniques can be an effective tool to balance activity and rest. Below are some pacing tips which some people living with pain have found to be useful.

*Source: LeFort, Sandy, et al. Living a Healthy Life with Chronic Pain (Chapter 6). Boulder: Bull Publishing, 2015.*

1. Determine how you spend your time. Monitor your daily activities, including your rest periods, to find out how you spend your time. Keep a diary for a couple of days. Choose a typical weekday and a day on the weekend. Note what activities you do and how long you can do them before you are bothered by pain. Note your rest periods and how long you rest.

2. Make a schedule. Develop an activity schedule that includes rest breaks and stick to your schedule. For example, take 5 minutes of rest for every 20 minutes of activity. This is very individual, depending on the type of activity.

3. Be time oriented, not pain oriented. Knowing how long you can do an activity before your pain gets worse means you can schedule specific activities for a certain number of minutes before taking a rest for a specified period. This keeps you, not the pain, in control.

4. Rest before your pain starts to get worse. So many times, we want to complete activities and push through pain. Keep to your schedule. Stop. Take a rest.

5. Incorporate change into your activity routine. A change may be as good as a rest. If you are in a situation where you can't take a rest break, alternate activities frequently, change body position, stretch, or go for a short walk.

6. Use a timer to signal breaks. Then you don't have to worry about trying to remember when to take your break.

7. Break tasks into smaller, more manageable pieces. Take breaks between the smaller tasks or schedule some smaller tasks on different days.

8. Avoid rushing. Slow down. Plan ahead. Rushing can increase your stress. Planning can save you energy and reduce frustration.

9. Don't overschedule activities. Work on developing realistic expectations of yourself. Sometimes, you just have to say no.

10. Prioritize your activities. Some days, you may not be able to get everything done. Determine the most important thing you want to accomplish today and then work on that.

# Appendix H: Blank Pacing Worksheet

**Body**

**Brain**

**Behavior**

**Chronic**

**pain**

[(click here for printable copy)](https://depts.washington.edu/aim-cp/wordpress/wp-content/uploads/2025/08/Pacing-Worksheet-Instructions.pdf)

| Task | Time 1 | Time 2 | Time 3 | Start* | Times a day | Day 1 | Day 2 | Day 3 | Day 4 | Day 5 | Day 6 |
| --- | --- | --- | --- | --- | --- | --- | --- | --- | --- | --- | --- |
|  |  |  |  |  |  |  |  |  |  |  |  |
|  |  |  |  |  |  |  |  |  |  |  |  |
|  |  |  |  |  |  |  |  |  |  |  |  |
|  |  |  |  |  |  |  |  |  |  |  |  |

*A good rule of thumb is to take the average of your activity and subtract about 20% as a start. Take 1 +2 + 3 / 3 and subtract 20%! It is a good idea to do this on a calculator. Then, you can add 10% a day to increase in a paced way!

# Appendix I: Values

**Body**

**Brain**

**Behavior**

**Chronic**

**pain**

[(click here for printable copy)](https://depts.washington.edu/aim-cp/wordpress/wp-content/uploads/2025/10/Values.pdf)

One of the most important parts of managing chronic pain is to think about what makes life worth living for you. Pain can be a very tough barrier, and effective self-management of pain involves finding ways to overcome those barriers and re-engage in activities that you value. Many people find it helpful to think about things that would make their lives worth living, *even with pain.*

If you have trouble identifying values, here are some helpful prompts. Feel free to choose just one if the entire list is overwhelming!

1. What is one of your most dearly held memories? What is important about that memory, what does it say about you?
2. What do you most want to be remembered for?
3. What makes your life worth living? Sometimes it can help to discuss the idea of having a meaningful life as opposed to a happy life.
4. Imagine that you are at your 90^th^ birthday party, surrounded by loved ones. There is no limit to what could be shared. What would you want people to say about you and the kind of life you lived?
5. If it is too hard to think about positive values, you can think about the hardest about the pain. Sometimes, the pain is the flip of what is important. E.g. “I can’t take my daughter to work” might be flipped to “I want to be there for my family members”.

Once you have an idea of what your values are, you can pick one or two values to work on and begin doing things that help you move towards those values.

# Appendix J: Goal Setting and Creating Action Plans

**Body**

**Brain**

**Behavior**

**Chronic**

**pain**

[(click here for printable copy)](https://depts.washington.edu/aim-cp/wordpress/wp-content/uploads/2025/10/ActionPlan.pdf)

**These are the basics of a successful action plan:**

1. It is something you want to do.

2. It is achievable (something you can expect to be able to accomplish that week).

3. It is action specific.

4. It answers the questions What? How much? When? And How Often?

5. On a scale from 0 (not at all sure) to 10 (absolutely sure), you are confident you will complete your entire plan at a level of 7 or higher.

**In writing your action plan, be sure it includes all the following:**

1. What you are going to do (a specific action)

2. How much you are going to do (time, distance, repetitions, etc.)

3. When you are going to do it (time of the day, day of the week)

4. How often or how many days a week you are going to do it

Example: This week, I will walk (what) around the block (how much) before lunch (when) three times (how often)

My Action Plan

This week I will ______________________ (what)___________________________ (how much)

___________________________(when)____________________________________(how often)

# Appendix K: Body Skills

**Body**

**Brain**

**Behavior**

**Chronic**

**Pain**

[(click here for printable copy)](https://depts.washington.edu/aim-cp/wordpress/wp-content/uploads/2025/08/Body-Skills.pdf)

**Progressive Muscle Relaxation:** There are many [relaxation scripts available](https://sci.washington.edu/pain/). You can use progressive muscle relaxation, where the patient concentrates on tensing and relaxing each muscle group, starting from the feet and headed up to the head.

1. Start by lying or sitting down. Relax your entire body. Take five deep, slow breaths.
2. Lift your toes upward. Hold, then let go. Pull your toes downward. Hold, then let go.
3. Next, tense your calf muscles, and focus on the feeling of tension. Count 1...2...3...4...5. Then let go. Focus on the feeling of relaxation.
4. Move your knees towards each other and focus on the feeling of tension. Hold, then let go. Focus on the feeling of relaxation.
5. Squeeze your thigh muscles. Hold, then let go.
6. Clench your hands. Pause, then let go.
7. Tense your arms. Hold, then let go.
8. Squeeze your buttocks. Pause, then let go.
9. Contract your abdominal muscles. Pause, then let go.

10. Inhale and tighten your chest. Hold, then exhale and let go.

11. Raise your shoulders to your ears. Pause, then let go.

12. Purse your lips together. Hold, then release.

13. Open your mouth wide. Hold, then let go.

14. Close your eyes tightly. Pause, then release.

**A body scan script:**

Begin by making yourself comfortable. Sit in a chair and allow your back to be straight, but not stiff, with your feet on the ground. You could also do this practice standing or if you prefer, you can lie down and have your head supported. Your hands could be resting gently in your lap or at your side. Allow your eyes to close, or to remain open with a soft gaze.

Take several long, slow deep breaths. Breathing in fully and exhaling slowly. Breathe in through your nose and out through your nose or mouth. Feel your stomach expand on an inhale and relax and let go as you exhale.

Begin to let go of noises around you. Begin to shift your attention from outside to inside yourself. If you are distracted by sounds in the room, simply notice this and bring your focus back to your breathing.

Now slowly bring your attention down to your feet. Begin observing sensations in your feet. You might want to wiggle your toes a little, feeling your toes against your socks or shoes. Just notice, without judgement. You might imagine sending your breath down to your feet, as if the breath is traveling through the nose to the lungs and through the abdomen all the way down to your feet. And then back up again out through your nose and lungs. Perhaps you don’t feel anything at all. That is fine, too. Just allow yourself to feel the sensation of not feeling anything.

When you are ready, allow your feet to dissolve in your mind’s eye and move your attention up to your ankles, calves, knees and thighs. Observe the sensations you are experiencing throughout your legs. Breathe into and breathe out of the legs. If your mind begins to wander during this exercise, gently notice this without judgment and bring your mind back to noticing the sensations in your legs. If you notice any discomfort, pain or stiffness, don’t judge this. Just simply notice it. Observe how all the sensations rise and fall, shift and change moment to moment. Notice how no sensation is permanent. Just observe and allow the sensations to be in the moment, just as they are. Breathe into and out from the legs.

Then on the next out breath, allow the legs to dissolve in your mind. And move to the sensations in your lower back and pelvis. Softening and releasing as you breathe in and out. Slowly move your attention up to your mid back and upper back. Become curious about the sensations here. You may become aware of sensations in the muscle, temperature or points of contact with furniture or the bed. With each outbreath, you may let go of tension you are carrying. And then very gently shift your focus to your stomach and all the internal organs here. Perhaps you notice the feeling of clothing, the process of digestion or the belly rising or falling with each breath. If you notice opinions arising about these areas, gently let these go and return to noticing sensations. As you continue to breathe, bring your awareness to the chest and heart region and just notice your heartbeat. Observe how the chest rises during the inhale and how the chest falls during the exhale. Let go of any judgments that may arise. On the next outbreath, shift the focus to your hands and fingertips. See if you can channel your breathing into and out of this area as if you are breathing into and out from your hands. If your mind wanders, gently bring it back to the sensations in your hands.

And then, on the next outbreath, shift the focus and bring your awareness up into your arms. Observe the sensations or lack of sensations that may be occurring there. You might notice some difference between the left arm and the right arm – no need to judge this. As you exhale, you may experience the arm soften and release tensions. Continue to breathe and shift focus to the neck, shoulder and throat region. This is an area where we often have tension. Be with the sensations here. It could be tightness, rigidity or holding. You may notice the shoulders moving along with the breath. Let go of any thoughts or stories you are telling about this area. As you breathe, you may feel tension rolling off your shoulders.

**Chronic**

**Pain**

On the next outbreath, shift your focus and direct your attention to the scalp, head and face. Observe all the sensations occurring there. Notice the movement of the air as you breathe into or out of the nostrils or mouth. As you exhale, you might notice the softening of any tension you may be holding.

And now, let your attention expand out to include the entire body as a whole. Bring into your awareness the top of your head down to the bottom of your toes. Feel the gentle rhythm of the breath as it moves through the body.

As you come to the end of this practice, take a full, deep breath, taking in all the energy of this practice. Exhale fully. And when you are ready, open your eyes and return your attention to this present moment. As you become fully alert and awake, consider setting the intention that this practice of building awareness will benefit everyone you come in contact with today.

*from Shilagh Mirgain, Ph.D. for UW Cultivating Well-Being: A Neuroscientific Approach.*

# Appendix L: Sleep Hygiene

**Body**

**Brain**

**Behavior**

**Chronic**

**Pain**

[(click here for printable copy)](https://depts.washington.edu/aim-cp/wordpress/wp-content/uploads/2025/08/Sleep-Hygiene.pdf)

Recommendations

**1.** **Stick to a standard rise time.**  Use it every day regardless of the sleep you obtain on any night.

**2. Use the bed only for sleeping**. Do not read, eat, watch TV, etc. in bed. Sexual activity is the only exception.

**3. Get up when you can’t sleep.** When you are unable to sleep (i.e., sleep onset does not occur within about half an hour), get up and go to another room until you feel sleepy enough to fall asleep quickly before returning to bed. Get up again if sleep does not come on quickly.

**4.** **Don’t worry, plan, etc., in bed.** If such mental activities come on automatically in bed, get up and stay up until you can return to bed without these mental activities interfering with your sleep. If needed, you can jot down any worries or to-do items so you can take care of them in the morning.

**5. Avoid daytime napping.** Napping, particularly in the late afternoon or early evening may interfere with your night’s sleep.

**6**. **Go to bed when you are sleepy, but not before the time suggested.** Long periods of time in bed will lead to shallow, fragmented sleep. You should spend only the amount of time in bed that you need for sleep. Adherence to the bedtime and waketime suggested for you below should help you overcome your persistent sleep problem.

**7.** **Gradually establish regular activities and light exercise.**

**My** **earliest bedtime is: _______________**

**My standard rise time is: ________________**

**Other helpful practices:**

1. Limit caffeine & alcohol.

2. Avoid steady use of sleeping pills.

3. Schedule quiet time before bed.

4. Keep bedroom quiet, dark, and cool.

Above all, be patient! Your sleep problem developed over time so it will take some time to return to a more normal sleep pattern. By following the suggestions in this pamphlet, you should see gradual sleep improvements

Recommendations come from: CBT for Insomnia Treatment Manual, by Carney and Edinger

# Appendix M: Cognitive Change

**Brain**

**Behavior**

**Body**

**Chronic**

**pain**

[(click here for printable copy)](https://depts.washington.edu/aim-cp/wordpress/wp-content/uploads/2025/08/Thinking-Skills.pdf)

Catastrophizing / Cognitive Restructuring

**Cognitive Change Exercise**

Cognitive change or cognitive restructuring is a core CBT skill that helps you notice negative thinking patterns that add to your stress, which can worsen pain. Next time you notice a negative thought, see if you can take a brief pause and use the following worksheet to shift your perspective. Better yet, make time for this practice every day.

| **Catch It** | **Check It** | **Change it** |
| --- | --- | --- |
| Pay attention to negative or unhelpful thoughts or thinking. Ask yourself – *“What is my mind*  *telling me or getting caught up in?”* | Is thinking in this manner realistic? – is there evidence that supports or undermines the thought?    Are these thoughts reasonable – does they make sense?    Is thinking this way helpful? – does it fit with my values and goals?    Am I falling into any thinking traps? For example:    *Catastrophizing*: Focusing on the worst possible outcome, at the exclusion of other perspectives    *Mental filter*: Paying attention to the negative, without considering the whole picture    *All-or-nothing thinking*: Rigid thinking that doesn’t take nuance, or shades of gray into account    *Disqualifying the positive*: discounting positive changes or downplaying accomplishments    *Shoulds*: Holding oneself to an unreasonably high standard    *Personalization*: Taking on too much of the blame, without considering other factors | What is a more realistic, logical and helpful way of thinking about this?      How will thinking this way help me to act according to my values and goals?    Am I willing to let go of the struggle with the negative thought in the service of focusing on what really counts?    Is there another way to view this situation? |

Adapted from: Catch Check Change exercise from Think CBT, [www.thinkcbt.com](https://www.thinkcbt.com), and Challenging Unhelpful Thinking Styles from the Centre for Clinical Interventions, [www.cci.health.wa.gov.au](https://www.cci.health.wa.gov.au).

# Appendix N: Re-thinking the Pain Story

**Brain**

**Behavior**

**Body**

**Chronic**

**pain**

[(click here for printable copy)](https://depts.washington.edu/aim-cp/wordpress/wp-content/uploads/2025/10/ReThinking_PainStory.pdf)

**Cognitive Restructuring Worksheet**

This worksheet helps you notice unhelpful thoughts about pain—especially catastrophic ones like “I can’t handle this” or “It will never get better”—and replace them with more balanced, realistic ones. You may reproduce and adapt this worksheet freely for clinical and educational use.

**Step 1: Notice the thought**

What went through your mind when your pain got worse or you felt afraid?

Example: “This pain means I’m getting worse.”

**Step 2: Rate how much you believe that thought (0–100%)**

Example: 90%

**Step 3: What emotion(s) does that thought bring up?**

- ☐ Fear
- ☐ Hopelessness
- ☐ Anger
- ☐ Sadness
- ☐ Other: ___________

**Step 4: Look for thinking traps (check any that fit)**

- ☐ Catastrophizing (“This will ruin everything.”)
- ☐ All-or-nothing thinking (“If I can’t do it perfectly, I can’t do it at all.”)
- ☐ Fortune-telling (“It will always be this bad.”)
- ☐ Mind reading (“People will think I’m weak.”)
- ☐ Overgeneralizing (“It’s always like this.”)

**Step 5: Look at the evidence**

What facts support the thought? What facts go against it?

Example: “I’ve had pain spikes before, and they passed.”

**Step 6: Create a balanced, realistic thought**

Example: “This pain flare is uncomfortable, but it doesn’t mean I’m going backwards. I’ve handled it before.”

**Step 7: Re-rate how much you believe the original thought (0–100%)**

Example: Now 30%

**Step 8: Notice what changes**

How do you feel now?

Example: “A little calmer and more in control.”

**Optional Reflection Prompt**

“What can I remind myself next time pain feels scary?”

Example: “This is a flare, not a failure.”

# Appendix O: Safety Protocol for Suicidality

Although the study poses no serious risks to participants, participants may notify research personnel about pre-existing mental health issues that have not been previously identified. A suicide risk assessment protocol will be implemented by the nurse care manager and/or research coordinator at each site. Based on previous studies completed with this study population, we anticipate that the likelihood of suicide risk is low.

**Criteria for Suicide Risk Assessment**

A suicide risk assessment protocol will be implemented by a care manager under the following conditions:

1. *If a participant mentions or alludes to thoughts, intentions, plans or behaviors related to self-directed violence (SDV) outside of the context of formal assessment.*
2. *If a participant endorses a response > 0 on the Patient Health Questionnaire-9 (PHQ-9) item 9, meaning the participant has thoughts of being better off dead or of hurting themselves in some way “several days,” “more than half the days,” or “nearly every day.” This instrument will send an automated message to the care managers alerting them of a non-zero response, and care managers should try to follow up with participants within the next business day.*

**Online Survey Procedures**

Participants will most commonly complete the PHQ-9 online in the PainTracker software on their own once every six to eight weeks during the intervention phase of the study. To ensure subject safety in the online format, the study team will:

1. Immediately provide study participants with a PHQ-9 response > 0, with resources, including crisis lines, as they complete the survey. For instance:

- National Suicide Prevention Lifeline: 1-800-273-8255.
- Crisis Text Line: Text HOME to 741741

1. Include language in the consent statement that the study team does not check survey responses in real time but if someone chooses a response that may mean they are at risk of harming themselves, the study team will call to check in. Participants will be advised in resource messages to call 911 if they have an emergency.
2. Check PHQ-9 item 9 responses each business day and follow up with any participants via phone who endorse > 0 or otherwise appear to be at risk. Email alerts will be triggered by a score > 0 and sent to the care manager and the study team. The care manager will contact participants who endorse a 1 or 2 within three days and follow up with participants who endorse a 3 within 24 hours of checking the response. These follow-up calls will follow the procedures described below in the Suicide Risk Assessment Protocol.

**Suicide Risk Assessment Protocol: Nurse Care Manager**

The Nurse Care Manager (or other research staff if the care manager is not available) will use the Columbia-Suicide Severity Rating Scale (C-SSRS) to assess suicide risk. The C-SSRS is an evidence-based, widely used suicide risk assessment tool. The C-SSRS uses a series of simple, plain-language questions to help identify whether someone is at risk for suicide and assess the severity and immediacy of that risk.

If the participant meets criteria outlined above in the criteria for suicide risk assessment, the research staff member will use the C-SSRS to assess for severity of suicidal ideation as outlined on the C-SSRS form below. Study staff will follow the triage steps listed on the C-SSRS form (determined by each site) and will refer the patient to a clinician for further evaluation based upon risk level.

If a study staff member has reason to believe a participant is in grave danger (as would be the case extremely rarely, and only if they made explicit statements to this effect), then the staff member could contact local police and request a well-being check.

Study staff are encouraged and expected to discuss any concerns about a participant’s safety with a clinician or investigator, regardless of their scores on the C-SSRS.

**Referral to Clinicians and Clinician Protocol**

The study is led by clinicians who are credentialed and privileged providers at each site. Clinical care for the study is provided by licensed employees qualified to provide clinical care. Should there be any indication of risk for self-directed violence that arise during interactions with study staff, the study clinicians will follow the same specific procedures and policies that follow for assessing and managing risk at their respective site.

In instances where the clinician is concerned about safety/suicide risk in a study participant (i.e., if they state or allude to thoughts or plans of self-directed violence, mention recent self-directed violent behavior or behavior preparatory to self-directed violence during a study intervention session), or in instances where a clinician/investigator is contacted by a research staff member and asked to follow up with a participant, the clinicians/investigators will follow the same risk assessment and prevention protocol that is required of their respective institutions.

Study clinicians will document actions taken. An AE report will also be filed if required per the UW Human Subjects Division guidelines.

| **STUDY ID:** |  | **INTERVIEWER:** |  | **DATE:** |  |
| --- | --- | --- | --- | --- | --- |

*During your interview (or online assessment) you said that you have thoughts of being better off dead or of hurting yourself in some way. I’d like to ask you a couple more questions about those thoughts just to be sure that you’re safe.*

| **Ask questions that are in bold and underlined.** | **Past month** | |
| --- | --- | --- |
| **Ask Questions 1 and 2** | **YES** | **NO** |
| **1) Have *you wished you were dead or wished you could go to sleep and not wake up? If yes, describe:*** |  |  |
| **2) *Have you had any actual thoughts of killing yourself? If yes, describe:*** |  |  |
| **If YES to 2, ask questions 3, 4, 5, and 6. If NO to 2, go directly to question 6.** | | |
| **3) *Have you been thinking about how you might do this? If yes, describe:***  e.g. “*I thought about taking an overdose, but I never made a specific plan as to when where or how I would actually do it….and I would never go through with it.*” |  |  |
| **4) *Have you had these thoughts and had some intention of acting on them? If yes, describe:***  as opposed to “*I have the thoughts, but I definitely will not do anything about them*.” |  |  |
| **5) *Have you started to work out or worked out the details of how to kill yourself? Do you intend to carry out this plan? If yes, describe:*** |  |  |
| **6) Have you ever done anything, started to do anything, or prepared to do anything to end your life? *If yes, describe:***  Examples: Collected pills, obtained a gun, gave away valuables, wrote a will or suicide note, took out pills but didn’t swallow any, held a gun but changed your mind or it was grabbed from your hand, went to the roof but didn’t jump; or actually took pills, tried to shoot yourself, cut yourself, tried to hang yourself, etc. | **Lifetime** | |
|  |  |  |
| **If YES, ask: *Was this within the past 3 months?*** | **Past 3 Months** | |
|  |  |  |

| **RESPONSE PROTOCOL** | |
| --- | --- |
| **RISK STRATIFICATION** | **TRIAGE** |
| **HIGH RISK**  **Suicidal ideation with intent or intent with plan in past month (C-SSRS Suicidal Ideation #4 or #5)**  ***or***  **Suicidal behavior within past 3 months (C-SSRS Suicidal Behavior #6 *past 3 months*)** | - **Provide National Suicide Prevention Lifeline** (1-800-273-8255) and Crisis Text Line (text HOME to 741741). - **Call and hand off the participant to crisis counselors available at 988 for further evaluation.**  1. Verify the contact information and location of the participant and thank them for their candor and advise the participant that they will be connecting them with a mental health provider to perform a further assessment, *“Thank you for being honest with your answer(s). To ensure that you are safe and getting any help you might need I am going to ask a mental health provider to speak with you more about this.”* 2. If a clinician is not available immediately and/or if the research staff member perceives imminent risk, they will encourage the participant to seek immediate evaluation at the nearest ER and/or to contact the National Suicide Prevention Lifeline.   *If a study staff member has reason to believe a participant is in grave danger (as would be the case extremely rarely, and only if they made explicit statements to this effect), the staff member could contact local police and request a well-being check. In these rare cases, staff should make every effort to discuss the case first with a study clinician and notify the principal and co-investigators as soon as possible.* |
| **MODERATE RISK**  **Suicidal ideation WITHOUT plan, intent, or behavior in past month (C-SSRS screen #2 or #3)**  ***or***  **Suicidal behavior more than 3 months ago (C-SSRS Suicidal Behavior #6 *lifetime*)** | - **Provide National Suicide Prevention Lifeline** (1-800-273-8255) and Crisis Text Line (text HOME to 741741). - **Refer to clinician/primary care provider to contact within 48 hours for evaluation.**  1. Verify the contact information and location of the participant and thank them for their candor and advise the participant that they will be contacting a mental health provider to perform a further assessment. |
| **LOW RISK**  **Wish to die (C-SSRS Suicidal Ideation #1) no plan, intent or behavior**  ***or***  **Suicidal ideation more than 1 month ago WITHOUT plan, intent, or behavior (C-SSRS screen #2 or #3)** | - **Provide National Suicide Prevention Lifeline** (1-800-273-8255) and Crisis Text Line (text HOME to 741741). - **Review case with study clinician within 24 hours to determine if further action needs to be taken.** |

# Appendix P: Breathing to Relax the Body

**Body**

**Brain**

**Behavior**

**Chronic**

**Pain**


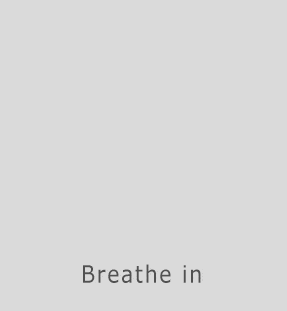


Calming the Body: Breathing and Relaxation

In CBT, we pay attention to different aspects of our experience, primarily our thoughts, feelings and behavior, to make changes that can lead to improving our well-being. Another CBT target is our physiology, which plays a crucial role in regulating much of the automatic function of the body, including breathing. A common experience of people with pain is pain-related anxiety, which can trigger a fight or flight response, which makes pain and distress even worse. Controlled breathing from the diaphragm counteracts this response by calming the body. Best of all, it can be done anytime, anywhere. This practice is known by many names, such as belly breathing, relaxed breathing and diaphragmatic breathing, but the essentials of the practice are the same.

**How to practice:**

Calm breathing involves taking steady, slow breaths from your belly (not your chest). You can practice sitting up or lying down, whichever is easier. A helpful starting point is to make sure that you are breathing from your belly – where your diaphragm is located – by placing your hand on your belly and your chest. Pay attention to the movement of your hand and ensure that only your hand on your belly moves. Step by step instructions:

1. Take a slow breath through your nose, breathing into your lower belly, for about four to five seconds. Make sure that the breath is paced, such that you are slowly breathing in throughout this step.
2. Hold your breath for a brief pause, a second or two at most.
3. Exhale through your mouth, extending your outbreath slowly, for about five to six seconds. As above, try to pace your outbreath so that you are breathing out the entire step.
4. Hold your breath for a brief pause and repeat the inbreath.

Try practicing for about five minutes every day.

Adapted from the Calm Breathing handout from Anxiety Canada

**Other resources to consider:**

-Mobile apps, like the Mindfulness Coach from the VA

-[Guided relaxation practices](https://sci.washington.edu/pain/) available from the University of Washington
