# Supplementary material for: Expanding rural access to chronic pain care through nurse care management: A hybrid type I effectiveness-implementation trial protocol
Source: PLoS One. 2026 May 27;21(5):e0349526. doi: 10.1371/journal.pone.0349526 (PMC13215515; doi:10.1371/journal.pone.0349526)
Supplement: S2 File — (DOCX) [file pone.0349526.s002.docx]

Adapting and Implementing a Nurse Care Management Model to Care for Rural Patients (AIM-CP) Study

AIM-CP Study

Protocol

**NCT06407115**

April 1^st^, 2026

**Protocol for the AIM-CP Study:**

A HYBRID TYPE I EFFECTIVENESS–IMPLEMENTATION TRIAL Adapting and Implementing a Nurse Care Management Model to Provide Comprehensive Coordinated Care for Patients with Chronic Pain in Rural Areas

**Principal Investigators:**

Sebastian Tong, MD, MPH

Associate Professor

Department of Family Medicine

University of Washington

Kushang V. Patel, PhD, MPH

Research Professor

Department of Anesthesiology and Pain Medicine

University of Washington

**Supported by:**

**National Institute of Nursing Research (NINR)**
*1UH3NR020930*

Contents

[PRÉCIS iv](#_Toc226800810)

[STUDY TEAM ROSTER 6](#_Toc226800811)

[PARTICIPATING STUDY SITES 7](#_Toc226800812)

[STUDY OBJECTIVES 8](#_Toc226800813)

[BACKGROUND AND RATIONALE 8](#_Toc226800814)

[STUDY DESIGN 10](#_Toc226800815)

[STUDY INTERVENTIONS 12](#_Toc226800816)

[STUDY PROCEDURES 14](#_Toc226800817)

[SAFETY ASSESSMENTS 20](#_Toc226800818)

[INTERVENTION DISCONTINUATION 22](#_Toc226800819)

[STATISTICAL CONSIDERATIONS 23](#_Toc226800820)

[DATA COLLECTION AND QUALITY ASSURANCE 25](#_Toc226800821)

[PARTICIPANT RIGHTS AND CONFIDENTIALITY 26](#_Toc226800822)

[PUBLICATION OF RESEARCH FINDINGS 27](#_Toc226800823)

[REFERENCES 28](#_Toc226800824)

# PRÉCIS

## Study Title

Adapting and Implementing a Nurse Care Management (NCM) Model to Care for Rural Patients with Chronic Pain: A Pragmatic Clinical Trial

## Objectives

Primary objective: To determine the effectiveness of the adapted NCM model vs. usual care for improving pain interference and intensity as measured by the Pain, Enjoyment of Life, and General Activity (PEG) scale.

Secondary objective 1: To evaluate the implementation of the adapted NCM intervention.

Secondary objective 2: To explore if there are disparities in response to the NCM intervention by examining heterogeneity in treatment effect in the primary (PEG scale) and secondary outcomes.

## Design and Outcomes

This is a pragmatic, hybrid type I effectiveness–implementation partially nested, individually randomized group-treatment trial of the adapted NCM model versus usual care in rural-dwelling patients with chronic pain.

Our outcome measures are modeled on the HEAL Initiative’s Common Data Elements^1^ and reflect our study’s focus on patient-reported outcomes. The primary outcome is pain interference and intensity as measured by the PEG scale. Secondary outcomes include physical function, sleep, pain catastrophizing, depression, anxiety, treatment satisfaction, substance use disorder, pain medication use/dosage including opioids, and healthcare utilization. Outcomes will be assessed at baseline, immediately after the 6-month intervention, and 6 months post-intervention.

*Diagram of the overall study design*


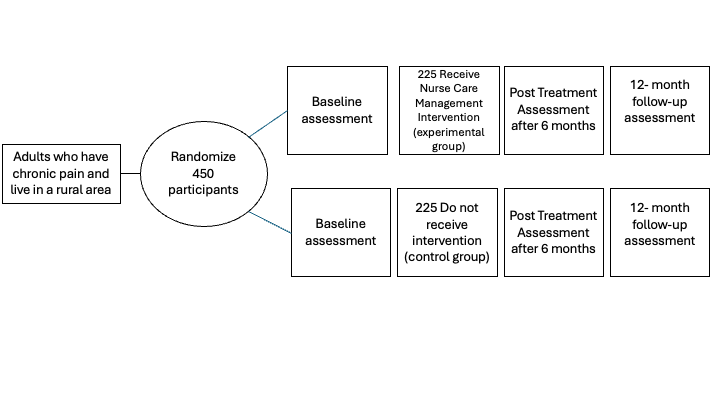


## Interventions and Duration

All patients in the intervention arm will receive care coordination. Care managers will schedule an initial 1-hour virtual appointment with each patient in the intervention arm of the study. In the intake appointment, care managers will assess the patient and collaboratively create a personalized care plan. During each subsequent visit, care managers will assess patient progress with their chronic pain treatment goals and make any needed adjustments. At three time points, care managers will invite participants to complete an additional assessment in a measurement-based care tool called PainTracker and use the results to inform the care plan. If sites opt to do so, care managers will write a brief note in the EHR communicating the care plan with the patient’s primary care clinician after the initial assessment and at the end of the intervention. Patients will participate in a series of 6 to 10, 30-45 minute, modular cognitive behavioral therapy (CBT) sessions delivered biweekly or monthly to address pain self-management, barriers to self-management, and common comorbidities (e.g., depression, anxiety, substance use, etc.). The aim of CBT is to develop strategies to change maladaptive cognition and behaviors around pain.

Additionally, care managers will refer patients to Tele-Enhance®Fitness (Tele-EF), help them enroll in classes on the website and monitor/encourage their engagement in Tele-EF. Once registered, patients will receive cuffed weights (necessary to participate in the program) and, if needed, a cellular-enabled tablet. Prior to starting Tele-EF classes, the community EF instructor will hold a “zero session” in which the patient will have a chance to log in to the virtual class, troubleshoot any technology challenges, and undergo a basic functional assessment (e.g., sit-to-stand test) so that the instructor can provide tailored exercise instruction. Patients will exercise for up to an hour 3 days per week for 16 weeks.

The total length of time each participant will be in the study intervention is 6 months

## Sample Size and Population

The target population is rural-dwelling patients with chronic pain. We will recruit a total of 450 adults with chronic pain, randomizing 225 to each treatment arm. Randomization assignments will be generated by the UW Data Coordinating Center. Patients will be randomly assigned to either the intervention or usual care using permuted block randomization with random block sizes of 4 and 6. Using block randomization ensures that equal numbers of participants are randomized to each arm and that the two groups are balanced at enrollment intervals.

# STUDY TEAM ROSTER

## Multiple Principal Investigators:

## Sebastian Tong, MD, MPH,

4311 11th Ave NE, Suite 210

Seattle, WA 98105

Phone: 206-616-1053

Email: setong@uw.edu

Key roles: Responsible for all aspects of the study, from implementation and supervision to scientific direction.

## Kushang V. Patel, PhD, MPH

1959 NE Pacific Street, Box 356540

Seattle, WA 98195-6540

Phone: 206-616-8052

Email: kvpatel@uw.edu

Key roles: Responsible for all aspects of the study. Oversee the

Tele-EF component of the intervention.

## Co-Investigators:

**Basia Belza, PhD, RN, FAAN, FGSA** Email: basiab@uw.edu

Key roles: Nursing expertise and help guide the adaptation and implementation of Nurse Care Management Model.

**Laura-Mae Baldwin, MD**

Email: lmb@uw.edu

Key roles: Provide assistance with care manager training.

**Bill Lober, MD, MS**

Email: lober@uw.edu

Key roles: Responsible for overseeing the adaptation of PainTracker.

**Andrew Humbert, PhD**

Email: ahumbert@uw.edu

Key roles: Provide biostatistics and data expertise for the study.

**Erika Steinbacher, MD**

Email: erika.steinbacher@atriumhealth.org

Key roles: Lead clinical contact for rural primary care clinics.

**Tom Ludden, PhD**

Email: Tom.Ludden@atriumhealth.org

Key roles: Responsible for identifying rural chronic pain patients from the Atrium Health System.

# PARTICIPATING STUDY SITES

The AIM-CP Study is a multi-site trial. The study is led by the University of Washington which also serves as a participating site and collaborates with Atrium Health Wake Forest Baptist in central North Carolina, Klamath Health Partnership in southern Oregon the, Washington Association for Community Health, and Providence Northeast Washington Medical Group.

**Atrium Health Wake Forest Baptist**

**Site PI: Hazel Tapp, PhD**

Email: Hazel.Tapp@atriumhealth.org

**Providence Northeast Washington Medical Group**

**Site PI: Caleb Holtzer, MD, MPH**

Providence Northeast Washington Medical Group – Family Medicine

Email: Caleb.Holtzer@providence.org

**Klamath Health Partnership**

**Site PI:**  **Tatum Foster, RN**

Email: tfoster@kodfp.org

**Washington Association for Community Health**

**Site PI: Kristina Alnajjar, MPH, RD**
 Email: kalnajjar@wacommunityhealth.org

# STUDY OBJECTIVES

## Primary Objective

**Aim 1. To determine the effectiveness of the adapted NCM model vs. Usual care for improving pain interference and intensity as measured by the PEG scale.**

*Hypothesis 1:* We hypothesize that the NCM model will be more effective in reducing pain interference and intensity than usual care.

## Secondary Objectives

**Aim 2. To evaluate the implementation of the adapted NCM intervention.**

**Aim 3. To explore if there are disparities in response to the NCM intervention by examining heterogeneity in treatment effect in the primary (PEG scale) and secondary outcomes.**

# BACKGROUND AND RATIONALE

## Background on Condition, Disease, or Other Primary Study Focus

**Chronic pain –** Approximately 20% of the U.S. adult population has chronic pain with 8% experiencing frequent limits on daily function and work life^1^. Chronic pain reduces quality of life, functioning and productivity and is often associated with higher rates of disturbed sleep, anxiety, depression and substance use disorder.^2, 3^ Rural residents suffer from higher rates of chronic pain, are more likely to receive an opioid prescription and experience more comorbidities from chronic pain compared to nonrural-dwelling individuals.^4-7^ They are also less likely to receive physical therapy and to be taught pain self-management techniques.^8^ Approximately 14% of Americans (or 46 million individuals) live in rural communities and are more likely to face disparities in their chronic pain care and in their health outcomes.

Rural patients with chronic pain present in primary care: Over half of all people with chronic pain receive treatment in primary care,^9, 10^ given that demand has outstripped supply of specialty pain care services. This proportion is higher in rural communities. One in five visits in primary care is for chronic pain and has resulted in the over reliance on prescription opioids, despite evidence-based guidelines for care that do not rely on opioids.^11^ Managing chronic pain in primary care is complex and has been challenged by a limited primary care workforce.^12^ Both patients and primary care clinicians would benefit from interventions and guidance to improve access to evidence-based, non-pharmacologic treatments.

## Study Rationale

Nurse care management (NCM) has been implemented successfully to manage patients with other chronic conditions but not widely for patients with chronic pain. Care managers can provide a variety of functions including care coordination, linkages to community resources, and some counseling services. In AIM-CP, care managers will not only provide care coordination but also (a) be trained in CBT to address maladaptive thought patterns and behaviors around chronic pain and (b) facilitate participation in remotely delivered Tele-EF, an evidence-based exercise program. Our rationale is that both Tele-EF and CBT have been independently shown to improve pain, functioning, and quality of life and that care managers could facilitate patients in initiating and maintaining participation in such evidence-based services. Our long-term goal is to reduce the high burden of pain in rural areas by expanding access to this comprehensive, non-pharmacologic approach to chronic pain management. We propose a randomized controlled trial to test our adapted NCM model in rural patients who have chronic pain.

**Care coordination:** Meta-analyses have shown that care coordination for chronic diseases can improve functional status and mental health outcomes.^13, 14^ Specifically, care coordination for patients with chronic pain increases pain disability-free days and reduces pain intensity and interference.^15-17^ In AIM-CP, the care coordination component will allow for coordination between available specialties and services for chronic pain, and be a means for patients to have their pain assessed and to develop goals of care. In addition, a social needs screening will be integrated so that care managers can address social needs that may affect chronic pain management. This responds to calls from national organizations to consider and address social needs in healthcare and to enable patients to participate in the psychological and exercise components of our intervention.

**CBT:** The majority of chronic pain guidelines include psychological involvement as a core part of primary care treatment of chronic pain.^18^ Prior studies have demonstrated that behavioral health services improve patient-reported outcomes.^19, 20^ Chronic pain often co-occurs with mental and behavioral health conditions.^21, 22^ Psychological risk factors are associated with poor outcomes in primary care patients with ongoing chronic pain.^23-26^ These include distress, presence of depression or anxiety, passive coping strategies, and fear/avoidance beliefs. Their presence results in greater pain disability and decreased health related quality of life.^27^ The presence of depression and anxiety and perception of risk of persistence of lower back pain are most consistently linked with negative outcomes.^28^ Over 50% of patients with chronic pain have comorbid depression or anxiety related disorders.^24, 29, 30^ Up to 25% of patients with chronic pain have substance use disorders. ^24^ CBT can help with pain catastrophizing, pain coping, fear avoidance and self-efficacy. In addition, it can be behaviorally activating so that patients are motivated to participate in outside activities including but not limited to exercise programs. Overall, CBT has been shown to have sustained effects on reducing pain intensity and interference.^19, 30, 31^ Prior studies have shown that non-behavioral health professionals can be successfully trained in providing CBT.^32-36^ Given the limited access to behavioral health professionals in rural areas, we propose in AIM-CP to train care managers in the provision of modular CBT to patients with chronic pain.

**Tele-EF**: Physical exercise is recommended for many common painful conditions (e.g., knee osteoarthritis, low back pain).^37-44^ Indeed, several meta-analyses and systematic reviews have established that aerobic and strength training improves pain, physical function and other health outcomes among adults with different types of pain-related conditions.^45-51^ Despite clinical recommendations, studies have shown that exercise participation is low among adults with chronic pain. ^52-56^ Recognizing the benefits of exercise for chronic pain management, the CDC and other agencies have promoted evidence-based exercise programs that are group-based and led by instructors in the community.^57-59^ These programs improve pain and physical function and help address some obstacles to exercise participation, including lack of professional guidance and social support. However, many adults continue to face barriers to engagement in exercise programs.^60-65^ Barriers include inclement weather, access to facilities, and transportation. Environmental barriers are particularly challenging for rural residents who are unable to participate in community-based exercise programs because of limited or no access to transportation and exercise facilities.^66, 67^ In addition, a major environmental barrier to walking in rural areas is limited pedestrian infrastructure, including long distances between destinations and lack of sidewalks.^68^ The COVID-19 pandemic introduced an opportunity to engage rural residents in a virtual manner given the growth in telehealth and virtual offerings for activities.^69^ In AIM-CP, we propose to overcome barriers by providing patients access to a virtual group exercise program, Tele-EF. Tele-EF will not only overcome many of the barriers previously identified but also be behaviorally activating with group support and motivation

# STUDY DESIGN

**Overview:** A pragmatic, hybrid type I effectiveness–implementation partially nested, individually randomized group-treatment trial that will adapt and test the effectiveness of implementing the NCM model that includes care coordination, remotely delivered CBT and Tele-EF for rural patients with chronic pain. Patients who agree to participate in the study will be randomized to either the intervention group or the usual care group.

450 participants will be recruited from 2 WWAMI-region Practice-based Research Network (WPRN) sites: Providence Northeast Washington Medical Group and University of Washington, as well as three additional sites: the Atrium Health System in North Carolina, Klamath Health Partnership in southern Oregon, and Washington Association for Community Health.

Patients randomized to the intervention group will receive the three-pronged intervention package through the NCM model: 1) care coordination 2) CBT and 3) the Tele-EF program. Depending on care plan progress and retention, participants may be involved in intervention activities for up to 6 months. Patients randomized to the usual care group will receive their usual care for their chronic pain from their care team. Outcomes will be assessed for both groups at baseline, 6-month follow-up (post-intervention), and 12-month follow-up. The data variables are listed in Table 1. Patients will be able to complete surveys independently online in REDCap, via paper surveys in the mail, or with the assistance of a study coordinator over the phone.

## Selection and Enrollment of Participants

### **Inclusion Criteria**

Participants must meet all the following criteria to participate in this study:

1. Age 18 years or older
2. Current primary care patient of the healthcare system with a primary care visit in the last year
3. A chronic pain diagnosis in the EHR, experienced for at least 3 months
4. 3-item PEG score of ≥ 12^70^ (moderate or severe pain score)
5. Able to communicate in English and/or Spanish.
6. Living in a rural area as defined by the RFA^71^

### **Exclusion Criteria**

All candidates meeting any of the following criteria at baseline will be excluded from the study:

1. Unable or unwilling to give informed consent or accept randomization to either study group
2. Ongoing radiation or chemotherapy
3. Moderate or severe cognitive impairment, (self-report and t-MOCA assessment)
4. On palliative care or live in a nursing home or inpatient treatment facility
5. Scheduled surgical procedures in the next 6 months
6. Has received, in the past 6 months, pain-management related psychoeducation or skill training

## Study Enrollment Procedures

**Recruitment:** The eligibility criteria outlined above with recruitment techniques refined from the feasibility phase will be used during the recruitment process. Both the EHR query and provider referrals will be used to recruit potential participants. Providers at each health system will be trained by the study team and will be given the opportunity to opt out all or specific patients. Flyers will be distributed to providers to refer their patients to the study. Providers will be able to hand out these flyers to patients who will then be instructed by the flyer to complete a preliminary eligibility screener, or if they have questions, to reach out to the appropriate study contact by phone and/or email. Depending on the site, the appropriate study contact (a research coordinator or a local clinic staff member) will confirm patients meet the eligibility criteria and go through consent information. For the EHR query, patients will be identified with chronic pain from health systems with the methodology as described by Mayhew.^72^ The health systems will send eligible patients an introductory letter or email on their letterhead with an opt-out option. If patients do not opt out, they will be contacted by phone by the research team (up to 5 contact attempts per eligible patient). We seek to recruit 270 patients from the 4 WPRN-affiliated healthcare systems and 180 patients from Atrium Health.

**Enrollment:** Those who are eligible and agree to participate will be consented over the phone. Informed consent and HIPAA Authorization forms will be completed online in REDCap or on paper through the mail. After consent, required demographic information as per the HEAL Initiative Common Data Elements Program will be collected.^73^ Baseline data collection will occur via email/text (online REDCap surveys), mail or phone depending on patient preference.

**Randomization:** After patients complete the baseline assessment, research staff will randomize eligible patients using a centralized web-based portal that provides the next available assignment once an eligible patient consents for study participation. Randomization assignments will be generated by the UW Data Coordinating Center. Patients will be randomly assigned to either the intervention or usual care using permuted block randomization with random block sizes of 4 and 6. Using block randomization ensures that equal numbers of participants are randomized to each arm and that the two groups are balanced at enrollment intervals.

# STUDY INTERVENTIONS

## Interventions, Administration, and Duration

Patients assigned to the intervention group will be contacted by identified care managers from each health system. Once enrolled, patients will receive a three-pronged intervention package through the NCM model: 1) care coordination 2) CBT and 3) the Tele-EF program. Depending on care plan progress and retention, participants may be involved in intervention activities for up to 6 months. Outcomes will be assessed at baseline, post-intervention, and 6-month follow-up. Patients will be able to complete surveys independently online in REDCap, via paper surveys in the mail, or with the assistance of a study coordinator over the phone.

**Care Coordination:** Care managers will schedule an initial 1-hour virtual appointment with each patient. Prior to the meeting, care managers will review notes from each patient’s primary care doctor and available related notes from specialists/consultants. Care managers will initially meet with each patient to develop or review (if one already exists) their care plan for chronic pain. Care managers will ask patients to review the progression of their chronic pain, list previously tried treatments for chronic pain (including pharmacologic and nonpharmacologic treatments) and categorize each modality by their level of effectiveness. Patients will also be asked to identify their treatment goals for their chronic pain and develop realistic goals/expectations for their pain management. Patients will work with the care manager to complete a social needs survey that includes questions about housing, nutrition, exercise, social connections, mental health, financial needs, work/education, safety and transportation. The care manager will use the results to inform the context of care for the patient’s chronic pain and to connect patients with appropriate resources for their social needs. This will vary by the participating health system but may include connection to a practice and/or health system social worker and/or outside community resources found in local volunteer organizations, community centers, libraries, churches, health departments and/or educational centers. Care managers will also contact the primary care doctor to inform them that the patient will be participating in the intervention, summarize the care plan and obtain any relevant information from the clinician that could inform the care plan.

**PainTracker:** At the beginning, midpoint and end of the intervention period, patients will also report data about their pain and their mood using PainTracker, an online tool that has been utilized at the UW Center for Pain Relief for many years to assist patients in self-managing their pain. It has been shown to improve patient engagement in pain management and can also be used by the nurse care managers to monitor a patient’s pain and functioning. Each time the patient completes it, the care manager will review results with the patient and make recommendations for the care plan.

**Tele-EF:** EF is a widely disseminated, community-based program recommended by the CDC for arthritis. Nurse care managers will work with patients to make referrals to Tele-EF. Referrals will be made on the study website where patients and NCMs will be presented with a list of class times. The EF instructors will then contact the patient to enroll them in classes. Once registered, patients will receive weights (necessary to participate in the program) and, if needed, a cellular-enabled tablet. If a tablet is provided, a member of our study team will work with the patients to address any technology questions. Prior to starting Tele-EF classes, the community EF instructor will hold a “zero session” in which the patient will have a chance to log in to the virtual class, troubleshoot any technology challenges, and undergo a basic functional assessment (e.g., sit-to-stand test) so that the instructor can provide tailored exercise instruction. Patients will exercise for up to an hour up to 3 days per week for up to 16 weeks.

**CBT:** Patients will be offered CBT to address pain self-management, barriers to self-management, and common comorbidities (e.g., depression, anxiety, substance use, etc.). We will use an adapted CBT for pain treatment instructor manual based on content developed and tested in other RCTs. This program and the accompanying materials were adapted from group-based CBT to one-on-one, remote delivery. The CBT intervention will include activities to address pain self-management skills in a patient-centric way. The intervention will require care managers to identify the patient’s goals and flex the modular CBT intervention targets and skills to align with those goals. Patients will participate in a series of 6-10, 30-45-minute bi-weekly or monthly individual sessions delivered by the care manager using a HIPAA-compliant video conferencing platform.

**Usual Care:** Those randomized to usual care will continue to follow-up with their usual care team for chronic pain management.

**Handling of Study Interventions and Adherence Assessment**

**Tele-EF:** Sound Generations is a key community partner in AIM-CP and is a non-profit organization that (a) provides services to older adults in Seattle/King County and (b) disseminates the EF program nationally. Sound Generations closely monitors their certified instructors in EF, ensuring treatment fidelity to the established exercise protocol through regular class audits.  Sound Generations maintains an online attendance and data tracking system which individual sites may use. All sites will track individual patient attendance to Tele-EF classes over 4 months. EF instructors record attendance data either directly through the Sound Generations online portal or securely transfer attendance data to Sound Generations to be uploaded. Sounds Generations will provide regular attendance data transfers to our team.

**Care Coordination, Cognitive Behavioral Therapy, and PainTracker:** For the care coordination component, we will track and evaluate adherence with completing PainTracker assessments prior to the care coordination meetings, number of care coordination visits, whether a pain management plan was developed, whether the social needs survey was administered, how identified needs were addressed and substantial variations between patients in the intervention (i.e., visit durations, types of plans, etc.). For the CBT component, we will track the consistency of the delivery of the CBT intervention between individuals, the level of adherence to the study manual, patients’ adherence to CBT visits and any necessary adaptations based on patients’ needs or expressed desires during visits.

**Concomitant Interventions**

**Allowed Interventions:** There are no study-specific restrictions on medication use. A medications inventory will be conducted at baseline and at post-intervention (month 6) from patient’s electronic health records to monitor pain-related medication use.

**Prohibited Interventions:** The study will exclude adults who are planning any major surgeries (including joint replacements) in the next six months. Additionally, adults who received in the past 6 months pain-management-related psychoeducation or skill training will be excluded.

# STUDY PROCEDURES

**Schedule of Evaluations** (see Table 1 on next page)

| **Assessment** | **Pre-screening** | **Screening, Enrollment, and Randomization** | **Baseline** | **Visit 0 with NCM** | **Routinely with Care Manager** | **Post Intervention** | **12 month follow-up** |
| --- | --- | --- | --- | --- | --- | --- | --- |
| **Research Assessments** | | | | | | | |
| Chronic Pain Diagnosis | **X** |  |  |  |  |  |  |
| Current Primary Care Patient | **X** |  |  |  |  |  |  |
| Demographic Data | **X** |  |  |  |  |  |  |
| Inclusion/Exclusion Criteria | **X** | **X** |  |  |  |  |  |
| PEG (3-item) |  | **X** |  |  |  |  |  |
| Rural Residence |  | **X** |  |  |  |  |  |
| t-MOCA, as indicated by self-reported cognitive impairment |  | **X** |  |  |  |  |  |
| PEG Pain interference and Pain Intensity |  |  | **X** |  |  | **X** | **X** |
| Informed Consent Form |  | **X** |  |  |  |  |  |
| Demographics |  | **X** |  |  |  |  |  |
| Medical History |  | **X** |  |  |  |  |  |
| PROMIS Physical Functioning Short Form 6b |  |  | **X** |  |  | **X** | **X** |
| PROMIS Sleep Disturbance 6a + Sleep Duration Question |  |  | **X** |  |  | **X** | **X** |
| Pain Catastrophizing Scale 6-item |  |  | **X** |  |  | **X** | **X** |
| PHQ-9 |  |  | **X** |  |  | **X** | **X** |
| GAD-7 |  |  | **X** |  |  | **X** | **X** |
| Patient's Global Impression of Change scale |  |  |  |  |  | **X** | **X** |
| TAPS-1 |  |  | **X** |  |  | **X** | **X** |
| Healthcare Utilization |  |  | **X** |  |  | **X** | **X** |
| Medications Inventory |  |  | **X** |  |  | **X** | **X** |
| Diagnoses codes |  |  | **X** |  |  |  |  |
| Social Needs |  |  |  | **X** |  | **X** | **X** |
| NCM Fidelity Checklist |  |  |  | **X** | **X** |  |  |
| UCLA Loneliness - 3-item |  |  | **X** |  |  | **X** | **X** |
| Falls |  |  | **X** |  |  | **X** | **X** |
| Physical Activity |  |  | **X** |  |  | **X** | **X** |
| Readiness for Change |  |  | **X** |  |  | **X** | **X** |
| **PainTracker to Guide Clinical Care** | | | | | | | |
| PROMIS-29 Pain interference sub scale |  |  |  | **X** | **X** |  |  |
| PHQ-9 |  |  |  | **X** | **X** |  |  |
| Physical Activity Screener (2-question) |  |  |  | **X** | **X** |  |  |
| GAD-7 |  |  |  | **X** | **X** |  |  |
| PROMIS Sleep Disturbance 6a + Sleep Duration Question |  |  |  | **X** |  |  |  |
| TAPS 1 and 2 |  |  |  | **X** |  |  |  |
| PC-PTSD-5 |  |  |  | **X** |  |  |  |
| Treatment history, goals/expectations of treatment, side effects, and interference with important activities |  |  |  | **X** |  |  |  |
|  | | | | | | |  |

## Description of Evaluations

**Screening:** Patients screened for eligibility must meet the following inclusion and exclusion criteria to participate. This screening will be completed via telephone with a study team member or self-guided in an online survey.

Screening inclusion criteria: (1) age 18 or older; (2) proficiently speaks English (or Spanish if site has Spanish-speaking NCM), (3) current health system patient with a visit in the last year (4) a documented chronic pain diagnosis in the EHR, experienced for more than 3 months (4) score of ≥ 12 on the 3-item Pain, Enjoyment of Life and General Activity (PEG) scale [range 0–30)], and (5) live in a rural area as verified with the HRSA Rural Health Grant Eligibility Analyzer.

Screening exclusion criteria: (1) Lives in a nursing home or in-patient treatment facility (2) receipt of palliative care (3) has received in past 6 months: pain management psychoeducation or behavioral skills training, (4) plans for surgery that required hospitalization within the next 6 months, (4) ongoing radiation treatment or chemotherapy (5) reports being told by a provider they have Alzheimer’s Disease or dementia AND/OR score 12 or below on the tMOCA for those that self-report any cognitive impairment.

Before consenting the patient, research coordinators will complete part one of the Eligibility Verification form and reference the data from EHR and the screening survey to verify 1) documented EHR diagnoses, 2) rurality with the HRSA tool, and 3) enter corresponding RUCA codes based on participant zip code with a HEAL initiative tool. The rest of the form can be completed during the consent call with the patients where the research coordinator may be prompted to verify 1) whether they have received care coordination services at your site, 2) whether they need an internet or device, or 3) whether the care coordinator/research coordinator needs to administer the tMOCA.

**Consenting Procedure:** Participants will be consented over the phone or videoconferencing software by the nurse care manager or a research coordinator. During the informed consent process, research staff will review the consent document and explain the study procedures, risks, benefits, and participation rights, as well as answer any questions the eligible participant may have. Participants will also fill out a HIPAA Authorization form to allow the study team to obtain medications, diagnoses codes, and healthcare utilization from their electronic health record. Importantly, study participants will be informed that they can withdraw from the study at any time. Participants will not be coerced or pressured to participate in the study. Participants will be provided a link to complete an electronic consent form in REDCap. Another option is mailing consent forms to the participants where they can sign and then take a photo of their signature and email signature back to research staff. Participants will be asked if they prefer baseline data collection to occur via email, mail or phone. The phone option allows patients with limited literacy or internet access to participate. For those who prefer the phone option, baseline instruments will be administered at the time of consent. For those who prefer email or mail, participants will be emailed or mailed the tools to them after consent.

**Enrollment:** Once eligible candidates and study staff complete the consenting process and sign the informed consent form, the study participant is considered enrolled into the study. Thus, the date of written informed consent is the date of study enrollment.

The 6-month period of the intervention will begin on the day they complete the baseline survey. The study team will attempt to have this appointment scheduled within 2 weeks of the date of study enrollment.

**Baseline Assessments:** Baseline assessments will be used to determine the efficacy of the study intervention. In addition, these assessments will also be used to characterize the study sample, determine whether the intervention groups are balanced with respect to important characteristics, and explore whether treatments vary across specific subgroups. The outcome measures were selected and modeled on the HEAL Initiative’s Common Data Elements1 and reflect our study’s focus on patient-reported outcomes. The primary outcome is pain interference and intensity as measured by the PEG scale. The PEG is a validated 3-item, 0-10 numerical rating scale that measures pain intensity and interference with enjoyment of life and general activity.^75^ We have chosen to use the PEG to measure our primary outcome since it is easy to administer and is the NIH HEAL Initiative’s preferred instrument for pain interference and intensity.^74^ Secondary outcomes including the remaining NIH HEAL Initiative core pain domains: pain intensity, physical functioning/QOL, sleep disturbance, pain catastrophizing, depression, anxiety, global satisfaction with treatment and substance use disorder. In addition, we will track pharmacologic treatments (medication names, doses and, if opioid, morphine milligram equivalents/day) from the EHR and patient-reported healthcare utilization over the course of the intervention period (number of hospital admissions, emergency department visits, urgent care visits and primary care visits). Baseline data collection will occur via email/text (online REDCap surveys), mail or phone depending on patient preference and will be carried out by trained research staff. Most of the self-reported questionnaire data will be collected during the baseline while pain assessment will be collected during the participants' meetings with the care manager.

- **Demographics and medical history** – age, sex, ethnicity, and socioeconomic data will be collected through a questionnaire in alignment with the NIH Heal Initiative Common Data Elements.
- **PainTracker** – Monthly ratings of pain intensity, functioning, mood, and sleep quality will be collected. Screeners for post-traumatic stress disorder (PTSD) and problem substance use will also be collected in the first assessment.
- **(PROMIS-57 Profile v2.0)** **PROMIS**– Patient reported health status short forms will be used to measure 3 domains, including Physical Function, Pain Interference, and Sleep Disturbance.
- **Well-being questionnaires:** we will collect anxiety, depression and subjective feelings of loneliness scores. (GAD-7/PHQ-9/ UCLA Loneliness - 3-item)
- **Pain questionnaires** – cognitive-related processes will be measured with the Pain Catastrophizing Scale. Pain interference and intensity will also be measured. (PEG (3-item) PEG Pain interference and Pain Intensity, Pain Catastrophizing Scale 6-item)
- **Substance Use Disorder** – Participants’ tobacco use, alcohol use, prescription medication misuse, and illicit substance use as measured by TAPS-1.
- **Medications inventory** – the name, dose, and frequency of use of medications will be collected from the electronic health record to monitor changes over time of analgesic medication use.
- **Diagnoses Codes –** the ICD-10 codes of each patient’s current problem list in the electronic health record will be collected at baseline to calculate a Charlson Comorbidity Index.
- **Health care Utilization-** Hospital admissions and Emergency Department, urgent care and primary care visits.
- **Falls questionnaire-** risk factor for falling
- **Readiness for change**

**Randomization:** Randomization will occur after completing the baseline assessment. The NCM intervention will begin **within 2 weeks of randomization.**

### **Follow-up Visits:** During the intervention/treatment phase, adverse events (AEs) will be collected. Regular visits with the Care Manager will occur using a video conferencing platform for those assigned to the intervention arm.

- **During treatment (0-6 months)**
- Adverse Events – participants will record daily whether they fell or had any exercise-related injury or health event in an online survey. Any report of an event or injury will be investigated further by the study staff and PI.

- **Visits with Care Manager (Visit 0)**
  - Social Needs Survey
  - Self-efficacy questionnaires
  - Routine PainTracker
    - PROMIS-29 v2. Pain interference sub scale
    - PHQ-9
    - Physical activity Screener
    - GAD-7
    - PROMIS Sleep Disturbance +Duration
    - TAPS 1 and 2
    - PC-PTSD-5
    - Treatment history, goals, expectations, side effects and interference
- **Subsequent visits with Care manager**
- Routine PainTracker every 6-8 weeks
- PROMIS-29 v2. Pain interference sub scale
- PHQ-9
- Physical activity Screener
- GAD-7

**6-month and 12-month follow-up**: The post-intervention assessment will be completed at 6 months, and a final follow-up will occur at 12 months to understand if the intervention effects are sustained. For participants who withdraw from the study early, study staff and PI will make every effort to work with the participant and collect the primary and secondary outcomes.

- **Pain Questionnaire**
- **PROMIS**
- **PHQ-9**
- **GAD-7**
- **TAPS-1**
- **Healthcare Utilization**
- **Medications inventory**
- **Patient Global Impression of Change (PGIC) and treatment satisfaction – overall change in health and satisfaction with the treatment and outcome will be assessed with the PGIC and treatment satisfaction questionnaire**
- **Falls Questionnaire**
- **Physical Activity**
- **Barriers to Care**
- **Readiness for Change**

# SAFETY ASSESSMENTS

Participant safety will be monitored once an individual is enrolled in the study. The expected adverse experiences for participating in the study are breach of confidentiality, psychological distress, and muscle soreness, joint pain, or musculoskeletal injury (e.g., ankle sprain) associated with the EF exercise program.

## Specification of Safety Parameters

## Methods and Timing for Assessing, Recording, and Analyzing Safety Parameters

During the study intervention period (6 months), participants will be asked to report any adverse events that they believe to be related to the study to a member of the research team or their Nurse Care Manager. If an adverse event is reported to a member of the research team, they will be instructed to inform the senior research coordinator who will discuss the AE with the MPIs and complete an AE form. Participants will complete a health status form at study month 6 and 12 when completing outcomes assessment that asks them to report any health issues that they think may be related to the study. Research staff will review responses and discuss the AE with the MPIs and complete an AE form. All AE forms will be saved on a secure UW server, and data will be entered into REDCap.

If a patient reports any level of suicidal ideation in the PHQ-9 in PainTracker, an automatic message will be sent to patients referring them to mental health resources, including national text chat lines and telephone hotlines. The NCM will also be alerted, and they will reach out to the patient to assess risk and offer resources according to the protocol in place at their local clinical site. The NCM will consult as needed with one of the clinician investigators. If the assessed level of risk is high (i.e., the patient is actively suicidal and has a plan), the NCM will instruct the patient to arrange for transport to an emergency department for immediate care. Otherwise, depending on frequency and persistence of the suicidal thoughts, the NCM will connect the participant with available resources in the community with close follow-up.

**Adverse Events and Serious Adverse Events**

An **adverse event (AE)** is generally defined as any unfavorable and unintended diagnosis, symptom, sign (including an abnormal laboratory finding), syndrome or disease which either occurs during the study, having been absent at baseline, or if present at baseline, appears to worsen. Adverse events deemed to be definitely or possibly related to the study will be recorded.

A **serious adverse event (SAE)** is generally defined as any untoward medical occurrence that results in death, is life threatening, requires inpatient hospitalization or prolongation of existing hospitalization, results in persistent or significant disability/incapacity, or is a congenital anomaly.

## Reporting Procedures

All AEs deemed possibly or definitely related to the study will be recorded as described in Section 8.2. The MPIs or study staff will contact participants who report an AE to study staff or through a health status form within 72 hours.

The MPIs have primary responsibility for the safety of participants as it relates to the study protocol. The MPIs will be responsible for reviewing AEs and assuring accurate and timely reporting of the AEs. The co-investigators will review, evaluate and classify AEs and provide follow-up for events until they are resolved. Any SAE or any event that results in injury to the participant while the participant is under the supervision of study-related personnel will be classified as a reportable adverse event (RAE). In accordance with the guidelines described in the DSMP, RAEs will be classified as to severity, expectedness, and potential relatedness to the study intervention using AE/SAE forms.

The MPIs will be responsible for reporting AEs and SAEs to the National Institute on Nursing Research (NINR), DSMB, and the University of Washington institutional review board (IRB) using their respective report formats. Since AEs are expected to be mild and SAEs are not anticipated, the AE/SAE reports will be submitted as required to NINR, DSMB, and IRB. If a SAE occurs that is related to the study intervention, the PI will notify NINR program officer and DSMB Chair within 48 hours of the study’s knowledge of the SAE. All deaths will be reported to the NINR program officer and DSMB Chair within 24 hours of the study’s knowledge of the death.

## Safety Monitoring

Although this is a minimal risk study, it is a Phase III clinical trial and therefore a DSMB is required according to NIH guidelines. The DSMB will act in an advisory capacity to the NINR Director to monitor participant safety, evaluate the progress of the study, and review procedures for maintaining the confidentiality of data. The DSMB will meet every 6 to 9 months via teleconference. Details about DSMB’s governance are described in the DSMB Charter and DSMP. The following are responsibilities of the DSMB:

- Review the research protocol, informed consent documents and plans for data safety and monitoring
- Recommend subject recruitment be initiated after receipt of a satisfactory protocol
- Evaluate the progress of the trial, including periodic assessments of data quality and timeliness, recruitment, accrual and retention, participant risk versus benefit, and other factors that can affect study outcome
- Consider factors external to the study when relevant information becomes available, such as scientific or therapeutic developments that may have an impact on the safety of the participants or the ethics of the trial
- Review study performance, make recommendations and assist in the resolution of problems reported by the PI
- Protect the safety of the study participants
- Report to NINR on the safety and progress of the trial
- Make recommendations to the NINR and the PI concerning continuation, termination or other modifications of the trial based on the observed beneficial or adverse effects of the treatment under study
- Ensure the confidentiality of the study data and the results of monitoring; and,
- Assist the NINR by commenting on any problems with study conduct, enrollment, sample size, and/or data collection

# INTERVENTION DISCONTINUATION

**Medical suspension (temporary discontinuation):** Participants who consecutively miss 3 or more NCM visits due to medical illness will be placed on medical suspension. If a participant informs the NCM that they have missed 3 consecutive visits due to illness, the NCM will inform the PIs and the participant will be placed on medical suspension.

Dr. Tong or other trained study staff will speak with the participant and/or their healthcare provider to determine when the participant can return to the program safely and be removed from medical suspension. Any participant on medical suspension for 60 or more days will be withdrawn from the study (because a total of 2 [33%] NCM visits would have been missed by that point).

**Intervention discontinuation:** If the study team (including the NCM) is concerned about the participant’s safety to continue in the study, then the participant will be withdrawn from the study. The study staff will make every effort to collect the primary and secondary outcomes as soon as possible (versus following protocol schedule).

**Voluntary withdrawal:** Study participants have the right to withdraw their consent from the study at any time. To formally withdraw, the participant should provide a written and dated notice of this decision to the PI. If the participant withdraws, study staff will ask permission to continue to follow the participant for outcomes assessment as originally scheduled.

**Replacement:** We do not plan to replace participants who discontinue participation in the study. The sample size calculation allows for up to a 20% loss of participants, which is a more liberal estimate based on the experience of previous trials with participants with chronic pain.

# STATISTICAL CONSIDERATIONS

## General Design Issues

**Primary Hypothesis:** The nurse care management model that includes care coordination, cognitive behavioral therapy (CBT), and referrals to a remotely delivered Enhanced Fitness exercise program (Tele-EF) will significantly reduce pain interference and intensity among rural primary care patients with chronic pain versus those randomized to usual care.

## Sample Size and Randomization

We plan to recruit 450 patients with chronic pain with the eligibility criteria listed in the patient eligibility section above. This sample size accounts for clustering within the intervention arm at the level of the care manager (intraclass correlation coefficient [ICC] = 0.05) and the Tele-EF instructor (ICC = 0.03). We assume 10 care managers with similar caseloads (approximately 22-23 participants each) and no more than five participants per Tele-EF instructor. Assuming a two-sided type 1 error rate of 0.05, conservatively estimated follow-up rate of 80% at 6 months, and a correlation between baseline and 6-month PEG scores of 0.3, this study can detect a moderate standardized effect size of d=0.48 on the PEG with >90% power.^76^ Under conservative assumptions, the study also has >90% power to detect standardized effect sizes of d = 0.40 on the 7 secondary outcome measures of Aim 1. We conservatively anticipate being able to recruit 5% of patients we contact and as such estimate that we will need to reach out to approximately 9000 patients to recruit for this trial. We seek to recruit 270 patients from the WPRN-affliated healthcare systems and 180 patients from Atrium Health. We will intentionally oversample from racial/ethnic minorities, those who identify as female and those who live in highly rural areas.

### **Treatment Assignment Procedures:** Eligible participants will be randomly allocated to intervention or usual care. A blocked randomization scheme stratified on health center will be used to approximately balance treatment group across health centers during recruitment. Random block sizes of 4 or 6 will be used. The randomization schedule will be developed by Dr. Wang (Co-I, Biostatistician) using Stata SE software.

### Dr. Wang will import the randomization allocation table into REDCap as a database separate from all other study data. Once a study participant has provided written informed consent and completed the baseline assignments, then research staff will access the randomization module in REDCap that will identify the next unassigned record in the allocation table and assign the participant to the intervention or usual care group designated in that record. The participant identification code and date are written to that record. Only Dr. Wang will have access to the randomization allocation table in REDCap.

## Interim analyses and Stopping Rules

Serious adverse events/unanticipated problems are expected to occur rarely and likely be unrelated to the interventions in this study. Any AEs that do occur are anticipated to be minimal and not severe. Therefore, a planned, formal interim analysis will not be conducted unless requested by the DSMB.

## Outcomes

### Primary outcome

Our primary outcome is pain interference and intensity as measured by the Pain, Enjoyment of Life and General Activity (PEG) scale.

### Secondary outcomes

Our secondary outcomes include physical function, sleep, pain catastrophizing, depression, anxiety, treatment satisfaction, substance use disorder, pain medication use/dosage including opioids, and healthcare utilization. All outcomes and the assessment timeline were previously described in Table 1.

## Data Analyses

Prior to analyzing data by study group, we will screen, clean, and transform data as needed. Baseline characteristics will be examined according to treatment group (Intervention vs. Usual care) using Student’s t and Fisher’s exact statistics. Statistical significance is defined as *P*<0.05. Statistical analyses will be completed using SAS version 19 and Stata SE version 16.

Statistical analyses will be performed using the intention-to-treat method, regardless of intervention adherence. The effects of the intervention will be assessed using data collected immediately post-treatment (study month 6) and at 6-month post-treatment follow-up (study month 12). We will apply linear mixed models specifying treatment assignment (Intervention vs. Usual care) as a fixed effect and each participant and health center as random effects to control for the correlation among repeated measurements on the same person and correlation among measurements of participants in the same health center. Importantly, we will specify a contrast statement to the mixed model to determine treatment efficacy at 6-month follow-up (study month 12), testing the time by intervention interaction. Similarly, the short-term effect will be examined by using a contrast statement comparing post-treatment (study month 6) means from primary and secondary outcome models. Maximum-likelihood techniques will be used to estimate parameters under the assumption of an unstructured covariance structure. If there is any statistically significant imbalance in the baseline characteristics of study participants between treatment groups (despite randomization), then we will adjust for those baseline values in the mixed models. The primary outcome will be analyzed at α = 0.05 since we compare a single treatment intervention (vs. control). For secondary outcomes, we will correct for multiple comparisons using the Hochberg sequential test procedure. As experts have noted, this approach for controlling the false discovery rate uses progressively more stringent thresholds and can achieve greater power to detect a true effect than the rigid Bonferroni procedure.

The results will be biased if missing data are related to the study outcomes. The longitudinal, mixed models include data from prior visits that would predict loss to follow-up and satisfy conditions for data considered Missing at Random (MAR). However, if there is informed censoring, then we will compare analyses using participants with complete data, multiple imputations, or explicit modeling of the censoring mechanism. As analytic approaches to missing data evolve, we will review the most recent literature prior to analysis and will apply the most appropriate method, such as marginal structural models.

In accordance with NIH policy, we plan to describe the study sample by sex/gender and race/ethnicity (e.g., the number and proportion who are women), and we will evaluate whether there are any between treatment group differences in the proportions of women and racial/ethnic minorities. After completing the pre-planned, primary efficacy analyses, we will conduct exploratory analyses to examine whether the effect of the intervention varied by major demographic characteristics, including age, sex/gender, and race/ethnicity; however, this trial is not powered statistically to detect subgroup differences in treatment effects. To our knowledge, there are no data from prior studies that strongly support the existence of significant subgroup differences in the effects of exercise or cognitive-behavioral skills training in adults with chronic pain.

# DATA COLLECTION AND QUALITY ASSURANCE

## Data Collection Forms

All telephone screening will be completed by trained staff at each site who will enter data directly into the study’s REDCap database, which is password protected and stored on secured servers at the University of Washington that are backed up daily. A unique screening identification code will be automatically generated in REDCap once the study staff save the telephone screen data entry form. Among candidates who are screened eligible and enrolled, they will receive a unique subject identification code that will be used for the remainder of the study. Study staff will send questionnaires via an online REDCap survey. Online surveys are directly linked to a subject identification code and data entered into surveys is stored directly in the REDCap database. If completing an online survey is not feasible, study staff will mail paper copies of the surveys with a return envelope. Upon receipt of completed surveys, study staff will enter responses into REDCap within 2 business days.

Electronic medical record data pulls will be completed by a data manager at each site. These records will be temporarily stored on secure servers maintained by each site. They will immediately be transferred to the UW REDCap via the file repository and deleted at the end of the study.

## Data Management

Dr. Jing Wang will serve as the data manager for the project and the MPIs will oversee all aspects of data management. The MPIs will consult with Co-Is on any issues that might occur. The MPIs will use the tracking function in REDCap to check the completeness of all online surveys and entry of surveys completed on paper.

The data entry forms in REDCap have branching logic and text restrictions built-in to reduce errors in data collection.

## Quality Assurance

### Training

All study staff will receive appropriate human subjects trainings and will be trained by the MPIs on procedures related to screening, consent, and data collection. New study staff hired throughout the project period will receive appropriate trainings. All study staff will attend monthly meetings to address questions related to the protocol and to provide additional trainings as necessary.

### Protocol Violations

Protocol violations are any unapproved changes, deviations or departures from the study design or procedures of a research project that are under the research team’s control and that have not been reviewed and approved by the IRB. Protocol violations are divided into two categories: major or minor. These violations may occur in the randomization process, intervention protocols, and in the timing or completion of data collection.

**Major protocol violations** are any unapproved changes in the research study design and/or procedures that are within the investigator’s control and not in accordance with the IRB-approved protocol that may affect the participant's rights, safety or well-being, or the completeness, accuracy and reliability of the study data.

Minor protocol violations (also known as protocol deviations) are any unapproved changes in the research study design and/or procedures that are within the investigator’s control and not in accordance with the IRB-approved protocol that do not have a major impact on either the participant’s rights, safety or well-being, or the completeness, accuracy and reliability of the study data.

The NIH Protocol Deviation Log/Form will be completed, dated, and signed for each protocol deviation/violation that may occur for each subject. This log will be stored in the participant’s file along with their consent form. The participant’s ID, description, category, code, and date of the deviation will be entered into REDCap.

### Monitoring

The MPIs and UW Senior Research Coordinator will review all study documentation (e.g., consent forms) on a biweekly basis. The MPIs will conduct data management tracking on a weekly basis.

# PARTICIPANT RIGHTS AND CONFIDENTIALITY

## Institutional Review Board (IRB) Review

The study protocol and informed consent document have been approved by the University of Washington IRB. Any subsequent modifications will be reviewed and approved by the University of Washington IRB.

## Informed Consent Forms

Prior to the baseline interview, participants will be sent a copy of the consent form. Documented consent will be obtained and recorded from each participant after confirming their study eligibility and reviewing all the study procedures, interventions, randomization, and time commitment. The consent form will describe the purpose of the study, the procedures to be followed, and the risks and benefits of participation. Signed forms will be collected electronically in REDCap or through the mail on paper copies.

## Participant Confidentiality

Participant confidentiality will be protected according to the Health Insurance Portability and Accountability Act (HIPAA) requirements. Individuals will sign HIPAA Authorization forms that allow the study team to access key data from their electronic health record. As described in section 10.1, all study participants will be assigned a unique study identification code that will populate on all data entry forms, documents, and files used for data analysis. Personal information needed for follow-up contact will be stored separately in REDCap from other data. Only the PIs and study coordinators at each site will have access to this file with personal information. REDCap Data Access groups will be utilized in REDCap to partition data access by site. All paper records (e.g., consent forms) will be kept in a locked file cabinet in the PI’s lab. Information will not be released without written permission of the participant, except as necessary for monitoring by the IRB, DSMB, and NIA.

## Study Discontinuation

The study may be discontinued at any time by the IRB, NINR, OHRP, or other government agencies as part of their duties to ensure that research participants are protected.

# PUBLICATION OF RESEARCH FINDINGS

Publication of the results of this trial will be governed by the consensus of the investigative team. Any presentation, abstract, or manuscript will be made available for review by the NINR.

# REFERENCES

1. Dahlhamer J, Lucas J, Zelaya, Carla, Nahin R, Mackey S, Debar L, Kerns R, Von Korff M, Porter L, Helmick C. Prevalence of Chronic Pain and High-Impact Chronic Pain Among Adults — United States, 2016. MMWR Morbidity and Mortality Weekly Report. 2018;67(36):1001-6. doi: 10.15585/mmwr.mm6736a2.

2. National Center for Complementary and Integrative Health. Chronic Pain: In Depth. 2018.

3. Institute of Medicine Committee on Advancing Pain Research CaE. Relieving Pain in America: A Blueprint for Transforming Prevention, Care, Education, and Research. Washington (DC): National Academies Press (US)

Copyright © 2011, National Academy of Sciences.; 2011.

4. Karmali RN, Skinner AC, Trogdon JG, Weinberger M, George SZ, Hassmiller Lich K. The association between the supply of select nonpharmacologic providers for pain and use of nonpharmacologic pain management services and initial opioid prescribing patterns for Medicare beneficiaries with persistent musculoskeletal pain. Health Serv Res. 2021;56(2):275-88. Epub 20201001. doi: 10.1111/1475-6773.13561. PubMed PMID: 33006158; PMCID: PMC7969208.

5. Prunuske JP, St. Hill CA, Hager KD, Lemieux AM, Swanoski MT, Anderson GW, Lutfiyya MN. Opioid prescribing patterns for non-malignant chronic pain for rural versus non-rural US adults: a population-based study using 2010 NAMCS data. BMC Health Services Research. 2014;14(1). doi: 10.1186/s12913-014-0563-8.

6. Hoffman PK, Meier BP, Council JR. A Comparison of Chronic Pain Between an Urban and Rural Population. Journal of Community Health Nursing. 2002;19(4):213-24. doi: 10.1207/s15327655jchn1904_02.

7. Tripp DA, Vandenkerkhof EG, Mcalister M. Prevalence and Determinants of Pain and Pain‐Related Disability in Urban and Rural Settings in Southeastern Ontario. Pain Research and Management. 2006;11(4):225-33. doi: 10.1155/2006/720895.

8. Eaton LH, Langford DJ, Meins AR, Rue T, Tauben DJ, Doorenbos AZ. Use of Self-management Interventions for Chronic Pain Management: A Comparison between Rural and Nonrural Residents. Pain Manag Nurs. 2018;19(1):8-13. Epub 20171115. doi: 10.1016/j.pmn.2017.09.004. PubMed PMID: 29153296; PMCID: PMC5807105.

9. Schneiderhan J, Clauw D, Schwenk TL. Primary Care of Patients With Chronic Pain. JAMA. 2017;317(23):2367. doi: 10.1001/jama.2017.5787.

10. Dubois MY, Follett KA. Pain Medicine: the case for an independent medical specialty and training

programs. Academic Medicine. 2014;89(6):863-8. doi: 10.1097/acm.0000000000000265.

11. Dowell D, Haegerich TM, Chou R. CDC Guideline for Prescribing Opioids for Chronic Pain—United States, 2016. JAMA. 2016;315(15):1624. doi: 10.1001/jama.2016.1464.

12. Petterson SM, Liaw WR, Phillips RL, Rabin DL, Meyers DS, Bazemore AW. Projecting US Primary Care Physician Workforce Needs: 2010-2025. The Annals of Family Medicine. 2012;10(6):503-9. doi: 10.1370/afm.1431.

13. Kastner M, Cardoso R, Lai Y, Treister V, Hamid JS, Hayden L, Wong G, Ivers NM, Liu B, Marr S, Holroyd-Leduc J, Straus SE. Effectiveness of interventions for managing multiple high-burden chronic diseases in older adults: a systematic review and meta-analysis. Canadian Medical Association Journal. 2018;190(34):E1004-E12. doi: 10.1503/cmaj.171391.

14. Schraeder C, Shelton P. Comprehensive care coordination for chronically ill adults: John Wiley &

Sons;. 2011.

15. Dickinson KC, Sharma R, Duckart JP, Corson K, Gerrity MS, Dobscha SK. VA healthcare costs of a collaborative intervention for chronic pain in primary care. Med Care. 2010;48(1):38-44. doi: 10.1097/MLR.0b013e3181bd49e2. PubMed PMID: 19952802.

16. Dobscha SK, Corson K, Perrin NA, Hanson GC, Leibowitz RQ, Doak MN, Dickinson KC, Sullivan MD, Gerrity MS. Collaborative Care for Chronic Pain in Primary Care. JAMA. 2009;301(12):1242. doi: 10.1001/jama.2009.377.

17. Benes LL, Keefe FJ, DeBar LL. Treating Persistent Pain: A Nurse Co-Led, Interdisciplinary Model for Primary Care. Pain Manag Nurs. 2022;23(6):728-36. Epub 20220731. doi: 10.1016/j.pmn.2022.07.004. PubMed PMID: 35922272; PMCID: PMC9771916.

18. Bruns D, Disorbio JM. Assessment of Biopsychosocial Risk Factors for Medical Treatment: A Collaborative Approach. Journal of Clinical Psychology in Medical Settings. 2009;16(2):127-47. doi: 10.1007/s10880-009-9148-9.

19. Ehde DM, Dillworth TM, Turner JA. Cognitive-behavioral therapy for individuals with chronic pain: efficacy, innovations, and directions for research. Am Psychol. 2014;69(2):153-66. doi: 10.1037/a0035747. PubMed PMID: 24547801.

20. Williams ACDC, Fisher E, Hearn L, Eccleston C. Psychological therapies for the management of chronic pain (excluding headache) in adults. Cochrane Database of Systematic Reviews. 2020;2021(11). doi: 10.1002/14651858.cd007407.pub4.

21. IsHak WW, Wen RY, Naghdechi L, Vanle B, Dang J, Knosp M, Dascal J, Marcia L, Gohar Y, Eskander L, Yadegar J, Hanna S, Sadek A, Aguilar-Hernandez L, Danovitch I, Louy C. Pain and Depression: A Systematic Review. Harv Rev Psychiatry. 2018;26(6):352-63. doi: 10.1097/hrp.0000000000000198. PubMed PMID: 30407234.

22. Dahan A, van Velzen M, Niesters M. Comorbidities and the complexities of chronic pain. Anesthesiology. 2014;121(4):675-7. doi: 10.1097/aln.0000000000000402. PubMed PMID: 25099749.

23. Goesling J, Lin LA, Clauw DJ. Psychiatry and Pain Management: at the Intersection of Chronic Pain and Mental Health. Current Psychiatry Reports. 2018;20(2). doi: 10.1007/s11920-018-0872-4.

24. Hooten WM. Chronic Pain and Mental Health Disorders. Mayo Clinic Proceedings. 2016;91(7):955-70. doi: 10.1016/j.mayocp.2016.04.029.

25. Bushnell MC, Čeko M, Low LA. Cognitive and emotional control of pain and its disruption in chronic pain. Nature Reviews Neuroscience. 2013;14(7):502-11. doi: 10.1038/nrn3516.

26. Glombiewski JA, Hartwich-Tersek J, Rief W. Depression in chronic back pain patients: prediction of pain intensity and pain disability in cognitive-behavioral treatment. Psychosomatics. 2010;51(2):130-6. doi: 10.1176/appi.psy.51.2.130. PubMed PMID: 20332288.

27. Ramond A, Bouton C, Richard I, Roquelaure Y, Baufreton C, Legrand E, Huez J-F. Psychosocial risk factors for chronic low back pain in primary care--a systematic review. Family Practice. 2011;28(1):12-21. doi: 10.1093/fampra/cmq072.

28. Bair MJ, Wu J, Damush TM, Sutherland JM, Kroenke K. Association of Depression and Anxiety Alone and in Combination With Chronic Musculoskeletal Pain in Primary Care Patients. Psychosomatic Medicine. 2008;70(8):890-7. doi: 10.1097/psy.0b013e318185c510.

29. Barnett K, Mercer SW, Norbury M, Watt G, Wyke S, Guthrie B. Epidemiology of multimorbidity and implications for health care, research, and medical education: a cross-sectional study. The Lancet. 2012;380(9836):37-43. doi: 10.1016/s0140-6736(12)60240-2.

30. Breivik H, Collett B, Ventafridda V, Cohen R, Gallacher D. Survey of chronic pain in Europe: Prevalence, impact on daily life, and treatment. European Journal of Pain. 2006;10(4):287-. doi: 10.1016/j.ejpain.2005.06.009.

31. Knoerl R, Lavoie Smith EM, Weisberg J. Chronic Pain and Cognitive Behavioral Therapy: An Integrative Review. West J Nurs Res. 2016;38(5):596-628. Epub 20151124. doi: 10.1177/0193945915615869. PubMed PMID: 26604219.

32. Andersen LS, Magidson JF, O’Cleirigh C, Remmert JE, Kagee A, Leaver M, Stein DJ, Safren SA, Joska J. A pilot study of a nurse-delivered cognitive behavioral therapy intervention (Ziphamandla) for adherence and depression in HIV in South Africa. Journal of Health Psychology. 2018;23(6):776-87. doi: 10.1177/1359105316643375.

33. Cajanding RJ. The Effectiveness of a Nurse-Led Cognitive-Behavioral Therapy on the Quality of Life, Self-Esteem and Mood Among Filipino Patients Living With Heart Failure: a Randomized Controlled Trial. Appl Nurs Res. 2016;31:86-93. Epub 20160120. doi: 10.1016/j.apnr.2016.01.002. PubMed PMID: 27397824.

34. Espie CA, Macmahon KMA, Kelly H-L, Broomfield NM, Douglas NJ, Engleman HM, Mckinstry B, Morin CM, Walker A, Wilson P. Randomized Clinical Effectiveness Trial of Nurse-Administered Small-Group Cognitive Behavior Therapy for Persistent Insomnia in General Practice. Sleep. 2007;30(5):574-84. doi: 10.1093/sleep/30.5.574.

35. Tanoue H, Yoshinaga N, Kato S, Naono-Nagatomo K, Ishida Y, Shiraishi Y. Nurse-led group cognitive behavioral therapy for major depressive disorder among adults in Japan: A preliminary single-group study. Int J Nurs Sci. 2018;5(3):218-22. Epub 20180627. doi: 10.1016/j.ijnss.2018.06.005. PubMed PMID: 31406828; PMCID: PMC6626260.

36. Zhang Q, Li F, Zhang H, Yu X, Cong Y. Effects of nurse-led home-based exercise & cognitive behavioral therapy on reducing cancer-related fatigue in patients with ovarian cancer during and after chemotherapy: A randomized controlled trial. Int J Nurs Stud. 2018;78:52-60. Epub 20170824. doi: 10.1016/j.ijnurstu.2017.08.010. PubMed PMID: 28939343.

37. Elliott AM, Smith BH, Penny KI, Smith WC, Chambers WA. The epidemiology of chronic pain in the community. Lancet. 1999;354(9186):1248-52. doi: 10.1016/s0140-6736(99)03057-3. PubMed PMID: 10520633.

38. Schopflocher D, Taenzer P, Jovey R. The Prevalence of Chronic Pain in Canada. Pain Research and Management. 2011;16(6):445-50. doi: 10.1155/2011/876306.

39. Bannuru RR, Osani MC, Vaysbrot EE, Arden NK, Bennell K, Bierma-Zeinstra SMA, Kraus VB, Lohmander LS, Abbott JH, Bhandari M, Blanco FJ, Espinosa R, Haugen IK, Lin J, Mandl LA, Moilanen E, Nakamura N, Snyder-Mackler L, Trojian T, Underwood M, McAlindon TE. OARSI guidelines for the non-surgical management of knee, hip, and polyarticular osteoarthritis. Osteoarthritis Cartilage. 2019;27(11):1578-89. Epub 20190703. doi: 10.1016/j.joca.2019.06.011. PubMed PMID: 31278997.

40. Kraus VB, Sprow K, Powell KE, Buchner D, Bloodgood B, Piercy K, George SM, Kraus WE. Effects of Physical Activity in Knee and Hip Osteoarthritis: A Systematic Umbrella Review. Medicine & Science in Sports & Exercise. 2019;51(6):1324-39. doi: 10.1249/mss.0000000000001944.

41. Rausch Osthoff A-K, Niedermann K, Braun J, Adams J, Brodin N, Dagfinrud H, Duruoz T, Esbensen BA, Günther K-P, Hurkmans E, Juhl CB, Kennedy N, Kiltz U, Knittle K, Nurmohamed M, Pais S, Severijns G, Swinnen TW, Pitsillidou IA, Warburton L, Yankov Z, Vliet Vlieland TPM. 2018 EULAR recommendations for physical activity in people with inflammatory arthritis and osteoarthritis. Annals of the Rheumatic Diseases. 2018;77(9):1251-60. doi: 10.1136/annrheumdis-2018-213585.

42. Kolasinski SL, Neogi T, Hochberg MC, Oatis C, Guyatt G, Block J, Callahan L, Copenhaver C, Dodge C, Felson D, Gellar K, Harvey WF, Hawker G, Herzig E, Kwoh CK, Nelson AE, Samuels J, Scanzello C, White D, Wise B, Altman RD, Direnzo D, Fontanarosa J, Giradi G, Ishimori M, Misra D, Shah AA, Shmagel AK, Thoma LM, Turgunbaev M, Turner AS, Reston J. 2019 American College of Rheumatology/Arthritis Foundation Guideline for the Management of Osteoarthritis of the Hand, Hip, and Knee. Arthritis Care & Research. 2020;72(2):149-62. doi: 10.1002/acr.24131.

43. Ambrose KR, Golightly YM. Physical exercise as non-pharmacological treatment of chronic pain: Why and when. Best Practice & Research Clinical Rheumatology. 2015;29(1):120-30. doi: 10.1016/j.berh.2015.04.022.

44. Foster NE, Anema JR, Cherkin D, Chou R, Cohen SP, Gross DP, Ferreira PH, Fritz JM, Koes BW, Peul W, Turner JA, Maher CG, Buchbinder R, Hartvigsen J, Cherkin D, Foster NE, Maher CG, Underwood M, Van Tulder M, Anema JR, Chou R, Cohen SP, Menezes Costa L, Croft P, Ferreira M, Ferreira PH, Fritz JM, Genevay S, Gross DP, Hancock MJ, Hoy D, Karppinen J, Koes BW, Kongsted A, Louw Q, Öberg B, Peul WC, Pransky G, Schoene M, Sieper J, Smeets RJ, Turner JA, Woolf A. Prevention and treatment of low back pain: evidence, challenges, and promising directions. The Lancet. 2018;391(10137):2368-83. doi: 10.1016/s0140-6736(18)30489-6.

45. Fransen M, Mcconnell S, Harmer AR, Van Der Esch M, Simic M, Bennell KL. Exercise for osteoarthritis of the knee: a Cochrane systematic review. British Journal of Sports Medicine. 2015;49(24):1554-7. doi: 10.1136/bjsports-2015-095424.

46. Fransen M, McConnell S, Hernandez-Molina G, Reichenbach S. Exercise for osteoarthritis of the hip. Cochrane Database Syst Rev. 2014;2014(4):Cd007912. Epub 20140422. doi: 10.1002/14651858.CD007912.pub2. PubMed PMID: 24756895; PMCID: PMC10898220.

47. Rausch Osthoff A-K, Juhl CB, Knittle K, Dagfinrud H, Hurkmans E, Braun J, Schoones J, Vliet Vlieland TPM, Niedermann K. Effects of exercise and physical activity promotion: meta-analysis informing the 2018 EULAR recommendations for physical activity in people with rheumatoid arthritis, spondyloarthritis and hip/knee osteoarthritis. RMD Open. 2018;4(2):e000713. doi: 10.1136/rmdopen-2018-000713.

48. O'Dwyer T, Durcan L, Wilson F. Exercise and physical activity in systemic lupus erythematosus: A systematic review with meta-analyses. Semin Arthritis Rheum. 2017;47(2):204-15. Epub 20170407. doi: 10.1016/j.semarthrit.2017.04.003. PubMed PMID: 28477898.

49. Kelley GA, Kelley KS, Hootman JM, Jones DL. Effects of community‐deliverable exercise on pain and physical function in adults with arthritis and other rheumatic diseases: A meta‐analysis. Arthritis Care & Research. 2011;63(1):79-93. doi: 10.1002/acr.20347.

50. Chou R, Deyo R, Friedly J, Skelly A, Hashimoto R, Weimer M, Fu R, Dana T, Kraegel P, Griffin J, Grusing S, Brodt ED. Nonpharmacologic Therapies for Low Back Pain: A Systematic Review for an American College of Physicians Clinical Practice Guideline. Ann Intern Med. 2017;166(7):493-505. Epub 20170214. doi: 10.7326/m16-2459. PubMed PMID: 28192793.

51. Geneen LJ, Moore RA, Clarke C, Martin D, Colvin LA, Smith BH. Physical activity and exercise for chronic pain in adults: an overview of Cochrane Reviews. Cochrane Database of Systematic Reviews. 2017. doi: 10.1002/14651858.cd011279.pub2.

52. Dunlop DD, Song J, Semanik PA, Chang RW, Sharma L, Bathon JM, Eaton CB, Hochberg MC, Jackson RD, Kwoh CK, Mysiw WJ, Nevitt MC, Hootman JM. Objective physical activity measurement in the osteoarthritis initiative: Are guidelines being met? Arthritis & Rheumatism. 2011;63(11):3372-82. doi: 10.1002/art.30562.

53. Murphy LB, Hootman JM, Boring MA, Carlson SA, Qin J, Barbour KE, Brady TJ, Helmick CG. Leisure Time Physical Activity Among U.S. Adults With Arthritis, 2008−2015. American Journal of Preventive Medicine. 2017;53(3):345-54. doi: 10.1016/j.amepre.2017.03.017.

54. Wallis JA, Webster KE, Levinger P, Taylor NF. What proportion of people with hip and knee osteoarthritis meet physical activity guidelines? A systematic review and meta-analysis. Osteoarthritis and Cartilage. 2013;21(11):1648-59. doi: 10.1016/j.joca.2013.08.003.

55. Dansie EJ, Turk DC, Martin KR, Van Domelen DR, Patel KV. Association of Chronic Widespread Pain With Objectively Measured Physical Activity in Adults: Findings From the National Health and Nutrition Examination Survey. The Journal of Pain. 2014;15(5):507-15. doi: 10.1016/j.jpain.2014.01.489.

56. Patel KV, Dansie EJ, Turk DC. Impact of Chronic Musculoskeletal Pain On Objectively Measured Daily Physical Activity: A Review of Current Findings. Pain Management. 2013;3(6):467-74. doi: 10.2217/pmt.13.46.

57. Office of the Surgeon G. Publications and Reports of the Surgeon General. Step It Up! The Surgeon General’s Call to Action to Promote Walking and Walkable Communities. Washington (DC): US Department of Health and Human Services; 2015.

58. Center for Disease Control and Prevention. Physical Activity Programs 2021.

59. Osteoarthritis Action Alliance. A National Public Health Agenda for Osteoarthritis: 2020 Update. 2020.

60. Bethancourt HJ, Rosenberg DE, Beatty T, Arterburn DE. Barriers to and Facilitators of Physical Activity Program Use Among Older Adults. Clinical Medicine & Research. 2014;12(1-2):10-20. doi: 10.3121/cmr.2013.1171.

61. Biedenweg K, Meischke H, Bohl A, Hammerback K, Williams B, Poe P, Phelan EA. Understanding Older Adults’ Motivators and Barriers to Participating in Organized Programs Supporting Exercise Behaviors. The Journal of Primary Prevention. 2014;35(1):1-11. doi: 10.1007/s10935-013-0331-2.

62. Farrance C, Tsofliou F, Clark C. Adherence to community based group exercise interventions for older people: A mixed-methods systematic review. Prev Med. 2016;87:155-66. Epub 20160224. doi: 10.1016/j.ypmed.2016.02.037. PubMed PMID: 26921655.

63. Stubbs B, Hurley M, Smith T. What are the factors that influence physical activity participation in adults with knee and hip osteoarthritis? A systematic review of physical activity correlates. Clin Rehabil. 2015;29(1):80-94. Epub 20140610. doi: 10.1177/0269215514538069. PubMed PMID: 24917590.

64. Wilcox S, Der Ananian C, Abbott J, Vrazel J, Ramsey C, Sharpe PA, Brady T. Perceived exercise barriers, enablers, and benefits among exercising and nonexercising adults with arthritis: Results from a qualitative study. Arthritis Care & Research. 2006;55(4):616-27. doi: 10.1002/art.22098.

65. Dobson F, Bennell KL, French SD, Nicolson PJ, Klaasman RN, Holden MA, Atkins L, Hinman RS. Barriers and Facilitators to Exercise Participation in People with Hip and/or Knee Osteoarthritis: Synthesis of the Literature Using Behavior Change Theory. Am J Phys Med Rehabil. 2016;95(5):372-89. doi: 10.1097/phm.0000000000000448. PubMed PMID: 26945211.

66. Doescher MP, Lee C, Berke EM, Adachi-Mejia AM, Lee C-K, Stewart O, Patterson DG, Hurvitz PM, Carlos HA, Duncan GE, Moudon AV. The built environment and utilitarian walking in small U.S. towns. Preventive Medicine. 2014;69:80-6. doi: 10.1016/j.ypmed.2014.08.027.

67. Parks SE. Differential correlates of physical activity in urban and rural adults of various socioeconomic backgrounds in the United States. Journal of Epidemiology & Community Health. 2003;57(1):29-35. doi: 10.1136/jech.57.1.29.

68. Lo B, Morgan E, Folta S, Graham M, Paul L, Nelson M, Jew N, Moffat L, Seguin R. Environmental Influences on Physical Activity among Rural Adults in Montana, United States: Views from Built Environment Audits, Resident Focus Groups, and Key Informant Interviews. International Journal of Environmental Research and Public Health. 2017;14(10):1173. doi: 10.3390/ijerph14101173.

69. Grundstein MJ, Fisher C, Titmuss M, Cioppa-Mosca J. The Role of Virtual Physical Therapy in a Post–Pandemic World: Pearls, Pitfalls, Challenges, and Adaptations. Physical Therapy. 2021;101(9). doi: 10.1093/ptj/pzab145.

70. DeBar L, Benes L, Bonifay A, Deyo RA, Elder CR, Keefe FJ, Leo MC, McMullen C, Mayhew M, Owen-Smith A, Smith DH, Trinacty CM, Vollmer WM. Interdisciplinary team-based care for patients with chronic pain on long-term opioid treatment in primary care (PPACT) - Protocol for a pragmatic cluster randomized trial. Contemp Clin Trials. 2018;67:91-9. Epub 20180306. doi: 10.1016/j.cct.2018.02.015. PubMed PMID: 29522897; PMCID: PMC5931339.

71. Department of Health and Human Services. HEAL Initiative: Prevention and Management of Chronic

Pain in Rural Populations. 2022.

72. Mayhew M, DeBar LL, Deyo RA, Kerns RD, Goulet JL, Brandt CA, Von Korff M. Development and Assessment of a Crosswalk Between ICD-9-CM and ICD-10-CM to Identify Patients with Common Pain Conditions. J Pain. 2019;20(12):1429-45. Epub 20190523. doi: 10.1016/j.jpain.2019.05.006. PubMed PMID: 31129316; PMCID: PMC6874733.

73. Initiative NH. Common Data Elements (CDEs) Program. 2022.
