# Supplementary material for: Expanding rural access to chronic pain care through nurse care management: A hybrid type I effectiveness-implementation trial protocol
Source: PLoS One. 2026 May 27;21(5):e0349526. doi: 10.1371/journal.pone.0349526 (PMC13215515; doi:10.1371/journal.pone.0349526)
Supplement: S4 File — (DOCX) [file pone.0349526.s004.docx]

**Data and Safety Monitoring Board Charter**

Adapting and Implementing a Nurse Care Management Model to Care for Rural Patients with Chronic Pain

Contact Principal Investigator: Sebastian Tong, MD

**Introduction**

The purpose of this charter is to define the responsibilities of the Data and Safety Monitoring Board (DSMB), detail membership requirements, describe the data to be reviewed, delineate the meeting process, and outline the considerations and policies of the DSMB. The DSMB will act in an expert, independent advisory capacity to monitor participant safety.

**Study Overview**

Using an individual randomized controlled trial design, the Principal Investigator proposes to adapt and implement a nurse care management model in health systems serving rural patients with chronic pain to provide care coordination, cognitive behavioral therapy, and referrals to a remotely delivered exercise program.

**DSMB RESPONSIBILITIES**

The DSMB’s responsibilities are to:

- Review the research protocol, informed consent documents, and plans for data safety and monitoring prior to initiation of study
- Conduct interim monitoring, the content of the data reports and meeting frequency as established in Table 1.
- Protect the safety of the study participants
- Review and evaluate *ad hoc* safety issues concerning the study at the request of the investigator or sponsor
- Make recommendations to the investigator or sponsor concerning continuation, termination, or other modifications of the study based on the observed beneficial or adverse effects of the study

**DSMB Membership**

The DSMB consists of 5 members who are clinicians with expertise in relevant clinical specialties for the study. The DSMB also includes at least one statistician knowledgeable about statistical methods for clinical research and analysis of research data. One DSMB member will serve as Chair.

The DSMB members are selected by the Chair and investigator. Although DSMB members are expected to serve for the duration of the study, in the unlikely event that a member is unable to continue participation, the reason will be documented and a replacement member will be selected by the DSMB Chair and investigator. The new member must have comparable expertise and qualifications to the DSMB member they are replacing.

This study’s DSMB members are outlined in the below table:

| Name / Title | Expertise |
| --- | --- |
| Frederick Chen, MD, MPH, Chief Health and Science Officer, American Medical Association | Primary Care Pain Management |
| Katie Porter, JD, MPH, Research Scientist, University of Washington **(DSMB Chair)** | Research Ethics |
| Roy Sabo, PhD, Professor of Biostatistics, Virginia Commonwealth University | Biostatistics |
| Richard Skolasky, ScD, Professor of Orthopedic Surgery, John Hopkins University | Exercise Specialist |
| Amy Starosta, PhD, Associate Professor of Rehabilitation Medicine, University of Washington | Psychology |

Conflict of Interest

Other than receiving compensation for their time spent as DSMB members, members should have no other relationship with the investigator or sponsor that could impair the members’ ability to objectively review study data as set forth below:

- DSMB members must not have any real or perceived scientific, financial, professional, personal, proprietary, or other conflict of interest related to the conduct, outcome, or impact of the study. This may include having been or being employed by the investigator or sponsor, having a fiduciary interest in the study or outcomes, conducting and/or managing the study, and/or having contact with participants during regular clinical care
- DSMB members must not be engaged in any simultaneously occurring competitive studies in any role that could pose a conflict of interest. DSMB members must also identify and disclose any concurrent service on other DSMBs of the same, related, or competing products
- DSMB members must be independent from the sponsor, IRBs, regulatory agencies, principal investigator, co-principal or sub-principal investigator, site investigator, site sub-investigator, steering committee, advisory board, Clinical Events Committee, clinical care of the study participants, or any other capacity related to study operations.

All DSMB members must disclose all possible conflict of interest in writing by signing a Conflict-of-Interest/Non-Disclosure Agreement prior to beginning service as a DSMB member.

Confidentiality

All materials, discussions, and proceedings of the DSMB are privileged and confidential. DSMB members agree to use this information exclusively to accomplish the responsibilities of the DSMB. No communication of the deliberations or recommendations of the DSMB, either written or oral, may occur except as required for the DSMB to fulfill its responsibilities. Individual DSMB members are expected to maintain confidentiality regarding the study outside the DSMB (including, but not limited to the investigators, IRBs, regulatory agencies, or sponsor) except as authorized by the DSMB.

**REVIEW OF SAFETY DATA**

The primary charge of the DSMB is to monitor the study for participant safety. The safety and related data the DSMB will review is outlined in Table 1.

Serious adverse events (SAEs) that are possibly or definitely related to study will be monitored by the DSMB Chair in real time throughout the study. SAEs must be reported by the investigator to the DSMB Chair within one working day of learning of the event. The DSMB’s process for reporting will be provided to the investigator at the beginning of the study. Unrelated SAEs should be reported in the interim data reports.

The DSMB reviews data related to study conduct and is listed in Table 1. The DSMB may issue recommendations regarding study conduct when concerns arise that aspects of study conduct may threaten participant safety or study integrity.

**Preparation of Report to DSMB**

Content of the Report to the DSMB

The data report should include information as outlined in Table 1 and the data reporting tables approved by the DSMB. Data should be presented by treatment group. The study statistician must be familiar with study details, including the design, setting, and objectives of the study, and have sufficient time and access to the data to provide insightful analyses responsive to the DSMB’s needs in the DSMB report.

Reports to the DSMB are distributed to DSMB members prior to a scheduled meeting.

**DSMB Meetings**

Projected Schedule of Meetings

An initial meeting of the DSMB will be held prior to any participant enrollment in order for the members to review the charter, form an understanding of the protocol and definitions being used, establish a meeting schedule, and finalize format and protocol-specified statistical methods to be used in reports to be considered by the DSMB. Subsequent DSMB meetings will be held to review and discuss study data according to the schedule as described in Table 1.

Table 1. Meeting frequency

| ***Timeline*** | ***Data Review by*** | ***Type of Data*** |
| --- | --- | --- |
| Prior to first enrollment | Full DSMB | Charter, protocol, data tables, consent form |
| When the first 30 participants have completed the study, then every six months | Full DSMB | Participant characteristics; recruitment, screening and enrollment; outcome and safety measures; summary of missed interventions, major protocol deviations, adverse events, non-compliance, and unanticipated problems |
| Serious Adverse Events | DSMB Chair | Event description and relevant clinical information |

*Ad Hoc* Meetings

An *ad hoc* meeting of the DSMB may be convened at any time by the DSMB Chair, investigator, or sponsor. if a significant safety concern arises during the study to review safety and any other aspect of the study. Significant safety events may include, but are not limited to, the following:

- A death or life-threatening condition sustained by a participant, regardless of causality
- An unexpected serious safety issue newly identified during the development program that could expose participants to unnecessary risks
- Any other concern regarding participant safety raised by any DSMB member, investigator, or sponsor.

Proposed study amendments that significantly alter the treatment plan and/or deal with participant safety concerns will prompt an *ad hoc* meeting of the DSMB for review prior to implementation of changes. This may require suspension of enrollment pending DSMB review.

Meeting Format

DSMB meetings will be conducted by teleconference and facilitated by the DSMB Chair or their designee, consisting of an open session and a closed session. A quorum, defined as a majority of members, is required to hold a DSMB meeting. For both open and closed sessions the NINR PO may attend at the discretion of the DSMB chair as an objective observer and will not provide additional information that may influence the recommendations of the DSMB. The Project Scientist may attend the open session, at the discretion of the DSMB chair.

Open Session

The investigator should attend the open session to present the information in the data report, and to be available to discuss the content of the report with the DSMB. The study statistician should attend, if possible. Other study team members and representatives of the sponsors may also attend the open session.

Closed Session

The closed session will be restricted to attendance to the DSMB members and the Data and Safety Administrator. Closed sessions also consist of review of the recommendations the DSMB wishes to make to the investigator and a formal vote.

Voting

DSMB recommendations will be agreed upon by formal majority vote. In event of a split vote, the DSMB Chair will cast the deciding vote.

**DSMB CONSIDERATIONS AND POLICIES**

After considering the information in the data report, the DSMB will determine whether the study should continue as planned, proceed with modifications, or be terminated. The justification to terminate the study may be due to the DSMB’s determination that there are overwhelming safety issues or operational hurdles.

Meeting Minutes

Minutes of DSMB meetings will be kept in two parts: open session and closed session. Meeting minutes should include at a minimum:

- Study title
- DSMB meeting date
- Copy of the meeting agenda
- A list of attendees, including DSMB members and any other persons present
- Copy of the presentation given by the investigator, if applicable
- Information reviewed and related discussion during the open session, including rationale for recommendations provided by voting DSMB members
- A copy of the DSMB recommendation letter

The DSM Administrator is responsible for recording and generating meeting minutes of both open and closed sessions.

*Closed Session*

Closed session meeting minutes will not be divulged beyond the DSMB until after the study is closed unless either:

- The DSMB voting members approve the release for the purpose of preserving the integrity of the study and the safety of participants, or
- The FDA or other regulatory authority requires disclosure

The investigator will receive a complete copy of the open and closed session meeting minutes at the completion of study.

DSMB Reports to the investigator

Following each meeting, the DSMB will issue a confidential letter or report separate from the minutes of the open and closed sessions that will be sent to the investigator. If no recommendations are made, the report may simply state, “The DSMB recommends that the study continue as planned.”

The DSMB Chair is available to the investigator to allow them the opportunity to ask questions and discuss any recommendations. If the investigator accepts the recommendations of the DSMB, the investigator will be responsible for implementing the actions in response. If the investigator rejects the DSMB’s recommendations, the investigator must provide the DSMB with a written explanation of their decision and supporting rationale. If the DSMB has recommended that the study be stopped but the investigator decides to continue the study, the investigator will inform all concerned regulatory authorities of its decision to continue the study despite the DSMB’s recommendation. Public disclosure of the decision to stop the study is at the discretion of the investigator or sponsor. The DSMB will not make any public announcements.

**OTHER**

Amendments to the DSMB Charter

This DSMB charter can be amended as needed during the study, as agreed upon by the DSMB.

Archiving

All DSMB documentation and records will be retained in a secured shared drive by the DSMB Program Manager. Documents will be retained at least two years after study discontinuation. Access to archived data will be controlled by the DSMB, which will release the information only as specified in this charter or as required by law.
