# Supplementary material for: Expanding rural access to chronic pain care through nurse care management: A hybrid type I effectiveness-implementation trial protocol
Source: PLoS One. 2026 May 27;21(5):e0349526. doi: 10.1371/journal.pone.0349526 (PMC13215515; doi:10.1371/journal.pone.0349526)
Supplement: Peer review history

# Editor report: Decision Letter 0

**DOI:** 10.1371/journal.pone.0349526.r001

**Authors:** Annesha Sil

30 Apr 2026

Expanding rural access to chronic pain care through nurse care management: a hybrid type I effectiveness-implementation trial protocol

PONE-D-26-18262

Dear Dr. Keiser,

We’re pleased to inform you that your manuscript has been judged scientifically suitable for publication and will be formally accepted for publication once it meets all outstanding technical requirements.

As a Peer-Reviewed Funded Protocol, your submission is eligible for expedited review by in-house editors. Based on our evaluation, we are satisfied that your manuscript meets our publication criteria for Study Protocols, and is therefore considered to be suitable for publication subject to final journal requirements.

Within one week, you’ll receive an e-mail detailing the required amendments. When these have been addressed, you’ll receive a formal acceptance letter and your manuscript will be scheduled for publication.

An invoice will be generated when your article is formally accepted. Please note, if your institution has a publishing partnership with PLOS and your article meets the relevant criteria, all or part of your publication costs will be covered. Please make sure your user information is up-to-date by logging into Editorial Manager at Editorial Manager® and clicking the ‘Update My Information' link at the top of the page. For questions related to billing, please contact billing support.

If your institution or institutions have a press office, please notify them about your upcoming paper to help maximize its impact. If they’ll be preparing press materials, please inform our press team as soon as possible -- no later than 48 hours after receiving the formal acceptance. Your manuscript will remain under strict press embargo until 2 pm Eastern Time on the date of publication. For more information, please contact onepress@plos.org.

Kind regards,

Annesha Sil, Ph.D.

Senior Editor

PLOS One

Journal requirements:

1. Thank you for stating the following financial disclosure: [This work was supported within (NIH) Pragmatic Trials Collaboratory by cooperative agreement UH3 NR020930 from the National Institute of Nursing Research (NINR). This work also received logistical and technical support from the NIH Pragmatic Trials Collaboratory Coordinating Center under award number U24 AT009676 from the National Center for Complementary and Integrative Health (NCCIH), the National Cancer Institute (NCI), the National Heart, Lung, and Blood Institute (NHLBI), the National Institute of Allergy and Infectious Diseases (NIAID), the National Institute of Arthritis and Musculoskeletal and Skin Diseases (NIAMS), the National Institute of Minority Health and Health Disparities (NIMHD), the National Institute of Neurological Disorders and Stroke (NINDS), NINR, the National Institute on Aging (NIA), the NIH Office of Behavioral and Social Sciences Research (OBSSR), and the NIH Office of Disease Prevention (ODP). The content is solely the responsibility of the authors and does not necessarily represent the official views of NINR or the NCCIH, NCI, NIA, NIAMS, NHLBI, NIAID, NIMHD, NINDS, OBSSR, or ODP, or the NIH. Infrastructural support for our research network was provided by the Institute of Translational Health Sciences, which is supported by the National Center for Advancing Translational Sciences of the National Institutes of Health through Grant Number UL1 TR002319. The contents of this manuscript are solely the responsibility of the authors and do not necessarily represent the views of the NIH. ].

Please state what role the funders took in the study.  If the funders had no role, please state: ""The funders had no role in study design, data collection and analysis, decision to publish, or preparation of the manuscript.""

If this statement is not correct you must amend it as needed.

Please respond by return e-mail so that we can amend your financial disclosure and competing interests on your behalf.

Reviewers' comments:

# Editor report: Acceptance letter

**DOI:** 10.1371/journal.pone.0349526.r002

**Authors:** Annesha Sil

PONE-D-26-18262

PLOS One

Dear Dr. Keiser,

I'm pleased to inform you that your manuscript has been deemed suitable for publication in PLOS One. Congratulations! Your manuscript is now being handed over to our production team.

At this stage, our production department will prepare your paper for publication. This includes ensuring the following:

* All references, tables, and figures are properly cited

* All relevant supporting information is included in the manuscript submission,

* There are no issues that prevent the paper from being properly typeset

You will receive further instructions from the production team, including instructions on how to review your proof when it is ready. Please keep in mind that we are working through a large volume of accepted articles, so please give us a few days to review your paper and let you know the next and final steps.

Lastly, if your institution or institutions have a press office, please let them know about your upcoming paper now to help maximize its impact. If they'll be preparing press materials, please inform our press team within the next 48 hours. Your manuscript will remain under strict press embargo until 2 pm Eastern Time on the date of publication. For more information, please contact onepress@plos.org.

You will receive an invoice from PLOS for your publication fee after your manuscript has reached the completed accept phase. If you receive an email requesting payment before acceptance or for any other service, this may be a phishing scheme. Learn how to identify phishing emails and protect your accounts at https://explore.plos.org/phishing.

If we can help with anything else, please email us at customercare@plos.org.

Thank you for submitting your work to PLOS ONE and supporting open access.

Kind regards,

PLOS ONE Editorial Office Staff

on behalf of

Dr Annesha Sil

Staff Editor

PLOS One
